# Integrative Analysis of the Ethanol Tolerance of *Saccharomyces cerevisiae*

**DOI:** 10.3390/ijms24065646

**Published:** 2023-03-15

**Authors:** Ivan Rodrigo Wolf, Lucas Farinazzo Marques, Lauana Fogaça de Almeida, Lucas Cardoso Lázari, Leonardo Nazário de Moraes, Luiz Henrique Cardoso, Camila Cristina de Oliveira Alves, Rafael Takahiro Nakajima, Amanda Piveta Schnepper, Marjorie de Assis Golim, Thais Regiani Cataldi, Jeroen G. Nijland, Camila Moreira Pinto, Matheus Naia Fioretto, Rodrigo Oliveira Almeida, Arnold J. M. Driessen, Rafael Plana Simōes, Mônica Veneziano Labate, Rejane Maria Tommasini Grotto, Carlos Alberto Labate, Ary Fernandes Junior, Luis Antonio Justulin, Rafael Luiz Buogo Coan, Érica Ramos, Fabiana Barcelos Furtado, Cesar Martins, Guilherme Targino Valente

**Affiliations:** 1 Department of Bioprocess and Biotechnology, School of Agriculture, São Paulo State University (UNESP), Botucatu 18610-034, Brazil; ivanr.wolf@gmail.com (I.R.W.);; 2 Department of Structural and Functional Biology, Institute of Biosciences, São Paulo State University (UNESP), Botucatu 18618-689, Brazil; 3 Laboratory of Applied Biotechnology, Clinical Hospital of the Medical School, São Paulo State University (UNESP), Botucatu 18618-970, Brazil; 4 Department of Parasitology, Biomedical Sciences Institute, University of São Paulo (USP), São Paulo 05508-000, Brazil; 5 Laboratório Max Feffer de Genética de Plantas, Escola Superior de Agricultura Luiz de Queiroz, Universidade de São Paulo (USP), Piracicaba 13418-900, Brazil; 6 Molecular Microbiology, Groningen Biomolecular Sciences and Biotechnology Institute, University of Groningen, Nijenborgh 7, 9747 AG Groningen, The Netherlands; 7 Zernike Institute for Advanced Materials, University of Groningen, Nijenborgh 7, 9747 AG Groningen, The Netherlands; 8 Instituto Federal de Educação, Ciência e Tecnologia do Sudeste de Minas Gerais–Campus Muriaé, Muriaé 36884-036, Brazil; 9 Laboratory of Bacteriology, Department of Chemical and Biological Sciences, Institute of Biosciences, São Paulo State University (UNESP), Botucatu 18618-689, Brazil; 10 Department of Biophysics and Pharmacology, Institute of Biosciences, São Paulo State University (UNESP), Botucatu 18618-689, Brazil; 11 Max Planck Institute for Heart and Lung Research, 61231 Bad Nauheim, Germany

**Keywords:** omics, data integration, systems biology, lncRNAs, lncRNA–protein interactions, membraneless organelles, CRISPR–Cas9

## Abstract

Ethanol (EtOH) alters many cellular processes in yeast. An integrated view of different EtOH-tolerant phenotypes and their long noncoding RNAs (lncRNAs) is not yet available. Here, large-scale data integration showed the core EtOH-responsive pathways, lncRNAs, and triggers of higher (HT) and lower (LT) EtOH-tolerant phenotypes. LncRNAs act in a strain-specific manner in the EtOH stress response. Network and omics analyses revealed that cells prepare for stress relief by favoring activation of life-essential systems. Therefore, longevity, peroxisomal, energy, lipid, and RNA/protein metabolisms are the core processes that drive EtOH tolerance. By integrating omics, network analysis, and several other experiments, we showed how the HT and LT phenotypes may arise: (1) the divergence occurs after cell signaling reaches the longevity and peroxisomal pathways, with CTA1 and ROS playing key roles; (2) signals reaching essential ribosomal and RNA pathways via SUI2 enhance the divergence; (3) specific lipid metabolism pathways also act on phenotype-specific profiles; (4) HTs take greater advantage of degradation and membraneless structures to cope with EtOH stress; and (5) our EtOH stress-buffering model suggests that diauxic shift drives EtOH buffering through an energy burst, mainly in HTs. Finally, critical genes, pathways, and the first models including lncRNAs to describe nuances of EtOH tolerance are reported here.

## 1. Introduction

Ethanol (EtOH) is the main metabolite produced from glycolysis-fermentation metabolism in *Saccharomyces cerevisiae*. EtOH flows between intracellular and extracellular environments through simple diffusion [1]. Therefore, either external or internal EtOH accumulation challenges cells, impacting cell growth and survival. EtOH primarily alters plasma membrane fluidity, causing cellular acidification, protein dysfunction, unbalanced molecule efflux, and reduced intake of glucose and other compounds. Thus, EtOH-stressed cells activate response mechanisms, modulating multiple processes [2,3,4].

Omics (transcriptomic, proteomic, metabolomic) analyses have revealed that the EtOH stress response in yeast involves several systems, including stress responses (homeostasis, heat shock proteins, and redox balance), cell structures (membrane components and vacuolar functions), signal transduction, RNA/protein synthesis, and metabolism (oxidative stress, amino acids, trehalose, energy, carbon, the TCA cycle, glycolysis and others) [5,6,7]. Network analyses revealed substantial network changes in cells under EtOH stress [5,8].

The EtOH stress response seems to be strain specific, complicating the understanding of this process. EtOH prompts diversity in network community organization, distinct metabolic adaptabilities and different stress responses through polymorphisms and chromosomal rearrangements [9]. Different phenotypes related to high tolerance to fermentation stressors rely on yeast strains, medium, intracellular accumulation, temperature and other factors [10].

Most of the *S. cerevisiae* genome, including many long noncoding RNAs (lncRNAs), is expressed under basal conditions. LncRNAs respond quickly to external stimuli, modulating gene expression, metabolism, stress response, and aging [11,12,13,14]. LncRNA–protein interactions facilitate the assembly of macromolecular complexes, inactivate target proteins, or assist either positively or negatively in protein complex organization. Therefore, lncRNAs recruit transcription factors, guide chromatin modifiers, act on histones, and engage in competitive binding to DNA-binding proteins [15].

Despite increasing interest in EtOH tolerance in yeast, information on the mechanisms by which strains respond to severe EtOH stress is scarce. For instance, systemic analysis considering massive data integration from strains with different phenotypes is not yet available. Furthermore, no studies have examined the roles of EtOH stress-responsive lncRNAs. Here, we focused on EtOH tolerance by integrating omics, cell and molecular biology, bioinformatics, modeling, network and mutant data, seeking features that differentiate between the higher EtOH tolerant (HT) and lower EtOH tolerant (LT) phenotypes.

We analyzed three HT (BMA64-1A, BY4742, and X2180-1A) and three LT (SEY6210, BY4741, and S288C) strains at their maximum EtOH tolerance levels. Only the basal pathways remained active under stress conditions to prepare cells for stress relief. In this case, pathways that regulate longevity, peroxisomal, energy/lipid, RNA and protein metabolisms are the main pathways that drive EtOH tolerance and phenotypic divergence. CTA1 directs signals toward longevity and peroxisomes, which are the first systems to exhibit phenotypic divergences in gene expression. LncRNAs act on these systems in a strain-specific manner. For instance, membraneless and storage and degradation systems containing particular lncRNAs promote EtOH tolerance in stressed cells. We also identified a relationship between decreased cell growth under stress and scarcity of sphingolipids, which was exacerbated in HT strains by sphinganine and squalene overload. Overall, we proposed an EtOH stress-buffering model: a diauxic shift promotes an energy burst under stress, and the HT strains seem to increase this burst by enhancing SDH activity. Finally, analysis of the CRISPR–Cas9 mutants generated here showed that CTA1, IXR1 and the lncRNAs transcr_20548, transcr_6448 (BMA64-1A), transcr_3536 (SEY6210), and transcr_10027 (BY4742) are critical for EtOH tolerance. The key points of our findings are summarized in this video: https://figshare.com/s/81d0676a05ae08e5a775 (accessed on 5 March 2023).

## 2. Results

### 2.1. Rationale and Overview

EtOH tolerance is the capacity of cells to survive transient and chronic exposure to EtOH. The highest EtOH tolerance level for a particular strain is the level that still allows poststress growth [3,16,17]. Here, a strain was 10% EtOH tolerant if it grew on YPD medium after 1 h of treatment with 10% EtOH but it did not grow when treated with ≥12% EtOH.

Different yeast strains can withstand distinct levels of EtOH stress [3,16,17]. Although similarities can be found, strains treated with the same EtOH concentration usually present significant differences in physiological responses [5,7,18,19,20,21,22,23,24,25]. A single EtOH concentration may be severe for one strain but not for another. Strain ‘A’ tolerates 5% EtOH stress, while strain ‘B’ tolerates 10%. When both strains are under 5% EtOH stress, only strain ‘A’ activates specific pathways. We cannot assume that strains ‘A’ and ‘B’ differ in their ability to cope with EtOH stress because 5% EtOH is harsh only for strain ‘A’. In this case, differences in physiological responses are likely to be related to stress level rather than severity. Therefore, we rationalize that to assess the core factors underlying the EtOH stress response in yeast, different EtOH tolerance phenotypes must be defined on the basis of the maximum tolerable EtOH concentration, i.e., one that still allows poststress growth. Here, we examined each strain under its maximum tolerable EtOH concentration to describe the systemic stress response in relation to EtOH severity.

**Figure 1:**
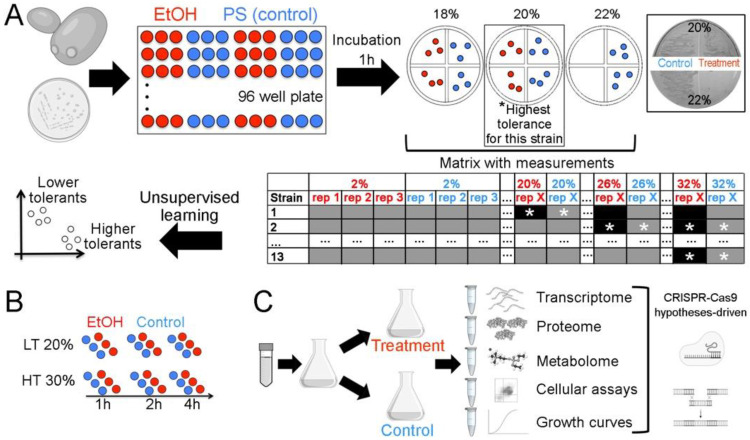
Experimental design. (**A**) Setting the phenotypes. PS: physiological solution; red and blue circles: treatment and control, respectively. The box shows examples of plates used to determine the highest EtOH tolerance. The experimental conditions that allowed or inhibited growth are represented by gray and black boxes, respectively. *: The highest EtOH level. (**B**) Time-course experiment. (**C**) Further experiments and hypothesis testing using mutants.

Here, the highest EtOH concentration tolerated by 13 different strains of *S. cerevisiae* was determined. The haploid strains were classified as HT or LT (Figure 1A): we selected only haploids for further analysis to mitigate the effect of potential allelic variations. The BMA64-1A (HT) and S288C (LT) strains were subjected to time-course experiments (2 h and 4 h) at their highest tolerated EtOH concentrations (Figure 1B). The additional experiments were scaled accordingly (Figure 1C). Strains were cultivated under aerobic conditions for all experiments.

The omics data integrated with network analyses allowed us to find EtOH stress-responsive lncRNAs and genes. Several laboratory experiments were performed to test our hypothesis or to validate gene/protein expression. The experiments included growth curve measurement, flow cytometry, colorimetric analysis, chromatography, mutant generation via CRISPR–Cas9, and Western blotting.

Finally, we argue that the definitions of the HT and LT phenotypes used here apply only to the context of this study because the number of strains analyzed is not representative of all *S. cerevisiae* strains. Therefore, we do not intend to extrapolate our conclusions regarding these phenotypes beyond the scope of this paper. However, our models may provide a basis for further studies.

### 2.2. Defining the Highest EtOH Tolerance for Each Strain

**Figure 2:**
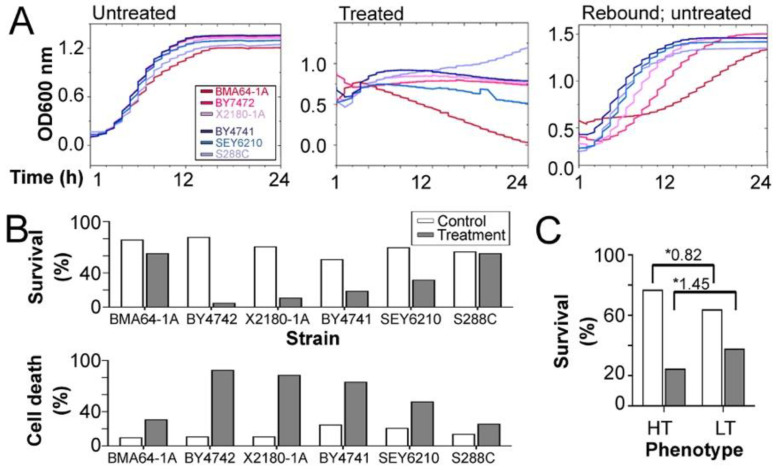
Cell growth and flow cytometry assays were used to evaluate EtOH stress severity. (**A**) Growth curve analyses of the populations of untreated cells, cells treated with the highest EtOH level previously defined, and the population rebound experiments (growth curves of the populations of cells inoculated in pure YPD medium after 1 h of treatment with the highest tolerated EtOH level). (**B**) Percentages of live and dead cells of each strain under control and treatment conditions. (**C**) Percentages of live cells of each phenotype under control and treatment conditions. The numbers above the bars are the “rate” comparing the average of the LT strains divided by the average of the HT strains. Hence, a rate > 1 indicates more live cells of the LT strains. *: Rate between LT divided by HT.

Thirteen yeast strains were independently treated with different concentrations of EtOH for 1 h (according to a classic study [17]) to define their highest tolerated EtOH concentration. Based on the highest EtOH tolerance level supported for each haploid strain (Supplementary Table S1), unsupervised learning classified BMA64-1A, BY4742 and X2180-1A into the HT phenotype, while SEY6210, BY4741 and S288C were classified into the LT phenotype (Table 1). All selected strains presented regular growth in YPD medium without any stressor (Figure 2A).

Our goal was to study cells under their highest tolerated EtOH stress level: the level that still allows for poststress growth. We performed experiments to assess the severity of EtOH stress level applied for each selected strain. The strains subjected to their maximum tolerated EtOH concentration for 24 h exhibited a quick transition to the stationary phase (Figure 2A; Supplementary Figure S1A,B). The flow cytometry assay with annexin V and propidium iodide costaining quantified the death rate of cells under severe EtOH stress. Each strain exposed to its highest EtOH stress level for 1 h showed a significantly increased number of dead cells. In this case, the HT strains exhibited a more pronounced decrease in survival rate (Supplementary Figure S2B,C; Supplementary Table S3). After 1 h of exposure to its highest EtOH stress level, each strain was immediately placed in regular YPD medium without any stressor. This experiment showed that all strains recovered population growth after stress relief (Figure 2A; Supplementary Figure S1A). Altogether, the findings confirmed that we had applied the most severe EtOH stress level that still allowed growth after stress relief. Moreover, we observed that the HT strains analyzed here were more strongly damaged by EtOH stress.

### 2.3. Gene, Protein and Metabolite Expression Analyses

We combined transcriptomic, proteomic, and metabolomic analyses to investigate EtOH stress-responsive processes. Detailed results of differential expression analysis using RNA-Seq (transcriptomic), proteomics and metabolomics are presented in the Supplementary Text (“Results”, topics “3.1”, “3.2”, and “3.3”). An interactive table with differentially expressed genes and metabolites is presented in Supplementary Datas S1 and S2, respectively.

Transcriptomic analysis revealed 1330 and 868 significant differentially expressed coding genes in the HT and LT strains, respectively. The HT and LT strains shared many differentially expressed genes and ontological functions, although phenotype specificities related to metabolism and nucleic acid processes were observed (Supplementary Figure S3; Supplementary Tables S4 and S5). KEGG pathway analysis showed enrichment of genes related to the TCA cycle, oxidative phosphorylation, RNA and ribosomal biogenesis in all the analyzed strains (Supplementary Table S6).

LC–MS/MS-based proteomics analysis identified 20 and 19 differentially expressed proteins in the HT and LT strains, respectively, including HSPs, ADH1, PDC1, elongation factors, ribosomal proteins and chaperones. Interestingly, the level of the P-body protein eIF5A was increased in LTs (Supplementary Figure S4).

GC–MS/MS-based metabolomics analysis identified >100 differentially abundant metabolites per strain (Supplementary Datas S2 and S3). EtOH phenotypically influenced the abundance of 16 and 9 metabolites in HTs and LTs, respectively (Supplementary Figure S5). We focused on previously reported metabolites or those whose abundances corresponded to the expression levels of the genes involved in their metabolism, including (1) glyceraldehyde and malate (increased in both phenotypes); (2) sphingosine (reduced in both phenotypes); (3) squalene (increased in HTs and reduced in LTs); (4) oxaloacetate, trehalose, and spermidine (reduced in HTs and increased in LTs); (5) inositol 1-phosphate (increased only in LTs); (6) fumarate (reduced only in HTs); and (7) quinolinate and sphinganine (increased only in HTs) (Supplementary Data S4).

### 2.4. LncRNA Assembly and General Functions of EtOH-Responsive lncRNAs

We identified 87–259 assembled lncRNAs (~161 per strain) for each strain under control and treatment conditions; detailed results for the assembly strategy are presented in the Supplementary Text (“Results”, topic “3.1”). As expected [14,26], lncRNAs identified here were expressed at lower levels than the coding genes, and their sequences were not conserved among strains or with yeast ncRNAs previously cataloged (Supplementary Tables S7 and S8; Supplementary Figures S6 and S7). The lack of sequence similarities shows that most of the lncRNAs identified here are strain-specific and comprise a new set of lncRNAs in yeast (https://figshare.com/s/e75fe1be9af623988be1, accessed on 5 March 2023).

As observed for their sequences, we hypothesize that the function and EtOH stress response of identified lncRNAs are strain specific. Due to the lack of sequence conservation, gene function inferences based on sequence similarities are inappropriate for testing our hypothesis. Therefore, we inferred the functions of lncRNAs based on the functions of their interacting proteins, regardless of their sequence. We tested our hypothesis that the function and stress response of lncRNAs are strain specific by comparing the expression levels and functions of their target proteins.

We computationally determined the lncRNA–protein interaction network of each strain: previous data [11,27,28] showed that lncRNAs physically interact with proteins in addition to regulating gene expression. The inferred lncRNA–protein networks (>0.95 of probability for each interaction) had an average of ~331.83 interactions per strain, ~207.16 target proteins and ~8.1 target proteins per lncRNA (Supplementary Table S9; Supplementary Data S5), consistent with yeast ncRNA–protein interactions [29,30].

Only four proteins were targeted by lncRNAs in the six strains analyzed, and the proteins targeted by lncRNAs within each phenotype (twelve for HT and fourteen for LT) were functionally diverse (Supplementary Figure S8). Thus, lncRNA functions may be strain- or phenotype-specific, regardless of strain or differential expression.

**Figure 3:**
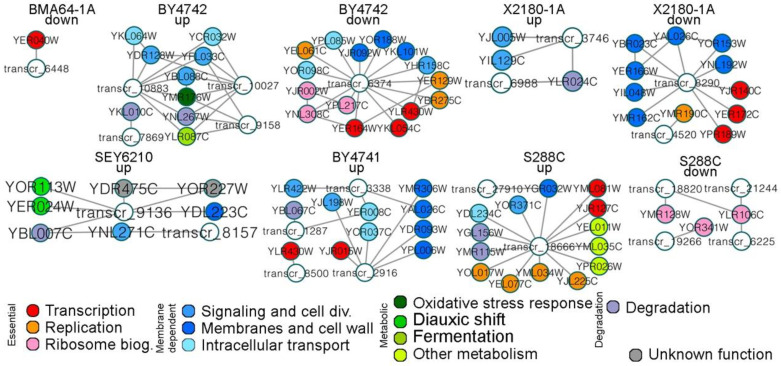
LncRNA propagation analysis of lncRNA–protein subnetworks associated with EtOH stress-responsive genes. The node colors are related to the biological functions depicted at the bottom of the picture. Only lncRNAs and proteins related to differentially expressed genes were evaluated in this analysis. The conjectures and basis for assigning the functions of lncRNAs presented here are reported in the “Supplementary Text” (“Discussion”, topic “1”).

We analyzed the function of EtOH stress-responsive lncRNAs to assess whether their response was strain specific; this analysis is hereafter referred to as lncRNA propagation analysis. We selected significant EtOH stress-responsive genes based on the differential transcriptomic expression of each strain. We selected proteins encoded by differentially expressed genes whose expression level matched that of the examined lncRNA. The systems signaling using the diffusion algorithm [31] throughout lncRNA–protein interactions revealed the relevant routes from each EtOH stress-responsive lncRNA of each strain. Next, we selected proteins surrounding the relevant routes to form subnetworks. For instance, (1) the upregulated genes “A”, “B”, “C” and “D” encoded proteins that interacted with the upregulated lncRNA “X”; (2) however, only proteins “A” and “D” surrounded the relevant route from the lncRNA “X” to “Y”; (3) therefore, the new subnetwork consists of lncRNAs “X” and “Y” and the coding genes “A” and “D”. The analysis corroborated our hypothesis that EtOH stress-responsive lncRNAs function in distinct pathways in a strain-specific manner. Interestingly, these pathways were generally associated with EtOH tolerance and response. Finally, we defined four broad functional categories for EtOH stress-responsive lncRNAs: life-essential, membrane-dependent, metabolic, and degradation process-related (Figure 3; Table 2; Supplementary Figure S26). EtOH stress-responsive molecules in those pathways are summarized in the Supplementary Text (“Results”, topics “4.3.2” and “4.3.3”).

### 2.5. Life-Essential Pathways Affected by EtOH

The aforementioned analysis of the lncRNA–protein network categorized life-essential (or essential) pathways as a broader category extensively affected by EtOH stress. Analysis also revealed that the EtOH stress response in the lncRNA–protein network is strain specific (Figure 3). We analyzed the EtOH stress response of essential pathways by examining networks that integrate multiple types of interactions and molecules. Integrative network analysis guarantees that the observed effect cannot be attributed to a specific system level. Overall, we investigated whether EtOH stress influenced essential pathways, the essential pathways that were most affected by this stress, and the mechanisms involved in triggering phenotypic divergences between HT and LT strains.

We evaluated whether EtOH stress influenced networks. We used each lncRNA–protein interaction and experimentally validated yeast protein–protein, gene regulatory and metabolic networks to create strain-specific networks. The transcriptomic expression data were used to build the control and treatment networks (see Supplementary Text, section ‘Methods’ and topic “5.6.2”). The diameter, path-length, and betweenness increased in most of the emulated treated networks, whereas the density, transitivity, number of connections and eigenvector decreased. Overall, network features were more strongly modified in most of the emulated treated networks, and the LT network under stress lost more highly connected genes than the HT network (Supplementary Tables S10 and S11; Supplementary Figure S9). Altogether, EtOH stress caused intense network rewiring in all the strains.

We sought to identify the essential pathways most affected by EtOH stress. The KEGG database contains networks of pathways with various types of interactions and molecules. Differentially expressed genes, metabolites, and strain-specific lncRNA–protein interactions were integrated into 95 KEGG pathways (https://figshare.com/s/396119809b1916c07a60, accessed on 5 March 2023). Manual curation of annotated functions and expression for genes and metabolites (Supplementary Data S6) and literature data [33,34] allowed us to identify the 12 essential systems most affected by EtOH stress (https://figshare.com/s/4b04ae15e5ab4f1b73c5, accessed on 5 March 2023). These systems included MAPK, TCA cycle, glycolysis and gluconeogenesis (Gly/gluc), peroxisome, longevity, autophagy, cell cycle, RNA degradation and transport, mRNA surveillance pathway, ribosome biogenesis and protein processing in the endoplasmic reticulum.

**Figure 4:**
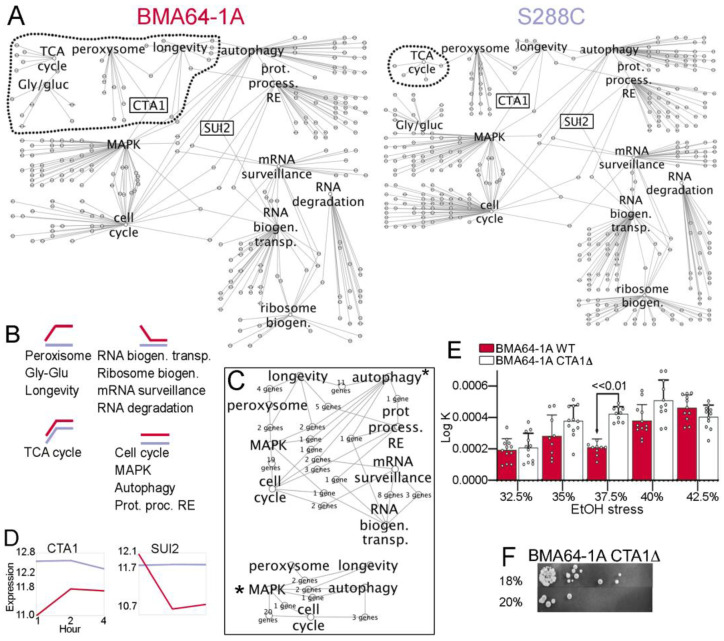
Essential subsystems showing phenotypic differences using BMA64-1A and S288C as models. (**A**) Comparison between the networks modeled based on the time-course and KEGG data. The dotted line delimits the communities with expression activation profiles. (**B**) Overview of the time-course expression profiles from the data shown in (**A**) under stress. (**C**) Analysis of information flow from pathways labeled with “*”. (**D**) Time-course landscapes of the crucial genes we suggest triggering the differences in the expression phenotype of cells under stress. (**E**) Population rebound after stress relief in WT and BMA64-1A CTA1Δ strains. (**F**) Spot test of the BMA64-1A CTA1Δ strain. The white dots (nodes) in (**A**) and (**C**) are genes/proteins.

The dynamics of gene expression and systemic flow were analyzed to identify how the system reacts to EtOH stress. Since KEGG presents pathway networks that are precisely delimited from each other, we first created a single pathway-integrated network. By modeling the selected essential pathways and their genes as nodes, the essential pathways were integrated by genes that work on these pathways (Supplementary Figure S10A). We used a clustering method [35] to merge the pathway integrated network and the time-course transcriptome data (1 h, 2 h and 4 h) of each EtOH-stressed BMA64-1A and S288C independently (https://figshare.com/s/537bb28192e48b8483c5, accessed on 5 March 2023); BMA64-1A and S288C were used as HT and LT models, respectively (see “Supplementary Text”, “Methods” topic “5.6.1”). We also observed extensive rewiring in all essential pathways induced by EtOH stress (Figure 4A). Gene expression profiles showed that peroxisome, Gly/gluc, and longevity were activated in BMA64-1A cells under EtOH stress, while the RNA and ribosome pathways were repressed. The TCA cycle was activated in both strains under stress, and the other pathways displayed stable expression in both strains (Figure 4B; Supplementary Figure S11). We also compared systems signaling from each pathway using the diffusion algorithm [31]. The data showed direct signaling from MAPK to peroxisomes, longevity, autophagy and the cell cycle. Signaling flows further from autophagy mainly to RNA biogenesis (Figure 4C). Finally, the information also flowed from longevity to peroxisomes, autophagy, and PPER, as well as from peroxisomes to the TCA cycle (Supplementary Figure S12; https://figshare.com/s/0ef043d4cd1d8b1f0bb1, accessed on 5 March 2023).

After receiving MAPK signals, the first pathways that showed significant expression differences between the HT and LT models were longevity and peroxisomes (Figure 4B,C). Therefore, we manually searched for genes surrounding these essential pathways that may have an effect on the emergence of the HT and LT phenotypes. CTA1 mediates MAPK signaling to the peroxisome and longevity. Peroxisome, longevity and CTA1 exhibited the same expression profile over time (activation in BMA64-1A and stable-like expression in S288C) (Figure 4A–D). By inactivating CTA1 in the BMA64-1A strain, we tested the hypothesis that CTA1 inactivation is associated with the EtOH phenotype. Our hypothesis was corroborated because the BMA64-1A CTA1Δ mutant matched the LT phenotype, with the tolerance to EtOH reduced to 18–20% and an improvement in population rebound after relief (Figure 4E,F).

Spermidine is a longevity agent in yeast and increases its lifespan [36]. EtOH stress affected spermidine yield (Supplementary Datas S2 and S4). Here, each strain was inoculated in YPD:EtOH:spermidine medium to examine the role of spermidine in the EtOH stress response. Spermidine overload boosted the growth of EtOH-stressed LTs and two HTs, including BMA64-1A. Only BMA64-1A downregulated SGF29 (Supplementary Data S1). As suggested, SGF29 may be related to spermidine’s positive effects [36].

Furthermore, RNA and ribosome metabolism-related genes in strain BMA64-1A (the HT model) exhibited repressed expression from 1 to 2 h of EtOH stress (Figure 4B). Therefore, we also sought to identify the genes responsible for this phenotypic variation. MAPK-autophagy signaling to RNA-related pathways is mediated by SUI2. The expression profiles of RNA pathways and SUI2 were similar over time (inactivation in BMA64-1A and stable-like in S288C) (Figure 4A–D). SUI2 causes transcriptional arrest in response to stress or nutritional deprivation [37]. The effect of SUI2 on RNA transcription was observed here since BMA64-1A cells exhibited a marked reduction in the total RNA yield after EtOH stress (Supplementary Figure S13).

### 2.6. Effect of EtOH on Degradation/Storage-Related Pathways

Degradation pathways constitute a broad category of pathways strongly affected by EtOH stress (Figure 3). Thus, we assessed which degradation/storage-related pathways, proteins and genes were affected and whether lncRNAs function in these processes. Manual curation of the transcriptomic, proteomic and metabolomic data was performed to determine the genes most affected. Western blotting, HPLC quantification, growth curve analysis and other experiments were performed to test the hypotheses and validate the expression results.

The proteomic analysis revealed that the abundance of proteins related to RNA/protein catabolism (e.g., chaperones and heat shock proteins) increased in EtOH-stressed cells. Elongation factors physically interact with P-bodies (PB) under stress [38], and their protein levels increased according to our proteomics analysis (Supplementary Figure S4).

**Figure 5:**
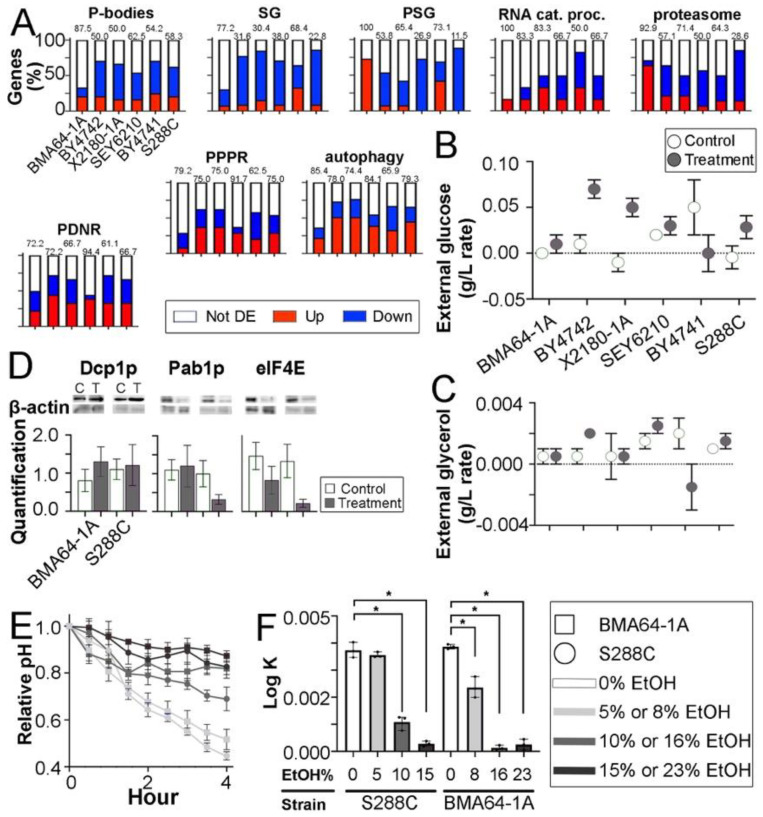
Analysis of genes related to EtOH stress-responsive storage and degradation mechanisms and external conditions of the medium for EtOH-stressed cells. (**A**) The numbers above the bars indicate the percentage of genes related to EtOH stress-responsive storage and degradation systems that were not differentially expressed (**D**,**E**) or upregulated. PB, P-bodies. SG, stress granules. PSG proteasome storage granules. RNA cat. proc., RNA catabolic process. PPPR, protein polyubiquitination and positive regulation of ubiquitination. PDNR, protein deubiquitination and negative regulation of ubiquitination. The full list of affected genes is reported in the Supplementary Text, ‘Results’, topic ‘4.3.5.’ (**B**,**C**) Glucose and glycerol levels. Each Y value represents a concentration rate between 1 and 0 h within the control and treatment groups. Therefore, higher values indicate lower glycerol yield or estimated glucose consumption after 1 h. (**D**) Western blot results. C and T over the bands indicate the control and treatment groups. (**E**) Normalized YPD pH level (see Equations (1) and (2) in the Supplementary Text, ‘Methods’, topic ‘3.1’). In the right box is depicted the colors and shapes of this graphic. (**F**) Cell growth in the pH quantification experiment (**E**). *, adjusted *p* value < 0.0001.

Based on transcriptomic data, we manually selected 191 genes related to PB, stress granules (SG), proteasome storage granules (PSG), the RNA catabolic process, proteasome and regulatory parts, protein polyubiquitination and positive regulation of ubiquitination (PPPR), protein deubiquitination and negative regulation of ubiquitination (PDNR) and autophagy. These systems and structures were selected because of their role in the degradation and storage pathways. Overall, ≤50% of genes related to RNA catabolism and PBs were downregulated, mainly in HTs (*p* value=0.0241). BMA64-1A had a large number of upregulated and nondifferentially expressed genes in the analyzed pathways. SG-disaggregase genes (HSP104 and SSE2) were usually upregulated. Decapping-related genes (DCS1 and 2 and DCP2) were usually not downregulated. Unlike other strains, in BMA64-1A, the essential genes for PB, SG, mRNA decay/surveillance and ribosome biogenesis and transport were not downregulated (Figure 5A; Supplementary Datas S1, S7 and S9). Altogether, the data suggest that EtOH stress affects degradation/storage pathways, including PB, SG, PSG, translation activity and mRNA decay.

Glucose starvation, stationary growth, oxidative stress and cytosol acidification are cytological conditions for the assembly of PB, SG and PSG [38,39,40,41,42,43]. DCP1a, PABP and eIF4E are used as molecular markers in yeast to study PB, SG and translation activity, respectively [42,44,45]. Therefore, we measured glucose, glycerol, extracellular acidification, reactive oxygen species (ROS) accumulation, DCP1a, PABP and eIF4E in EtOH-stressed strains to support the hypothesis that PB, SG and PSG may be assembled (Figure 5A).

HPLC analyses showed that most EtOH-stressed strains had lower glucose and glycerol concentrations, indicating glucose starvation and reduced glycerol efflux (Figure 5B,C). Previous findings showed that EtOH and glucose starvation acidify cells by inhibiting proton efflux. Yeast cells in the glucose-rich medium rapidly acidify the medium. Furthermore, internal and external pH are related [46]. Thus, we inferred the occurrence of cellular acidification in strains BMA64-1A (HT model) and S288C (LT model) from external pH quantification. In cells grown in medium with high and moderate EtOH concentrations, the cytosol was acidified, leading to a steady-state pH (Figure 5E,F; Supplementary Table S12). Flow cytometry showed that most stressed strains, especially HTs, increased the percentage of cells with mitochondrial and nuclear ROS (Supplementary Figure S2G; Supplementary Table S13; Supplementary Data S8). Western blot analysis showed that EtOH stress increased the Dcp1p level in BMA64-1A (HT model) and S288C (LT model) and decreased the eIF4E level. The PAB1p level increased in BMA641-1A and decreased in S288C (Figure 5D). Finally, we observed the stationary population growth phase during stress (Figure 2A). These results confirmed that EtOH induces the formation of an intracellular environment suitable for the assembly of membraneless organelles (PB, SG and PSG).

ROS may cause DNA damage [2], inducing defects in RNAs and proteins [47]. Inactivation of RAD53 and CHK1-PDS1 genes prevents DNA repair [48]: genes of DNA repair are downregulated in BMA64-1A and S288C (Supplementary Figure S29). Mitochondria are the main source of intracellular ROS [2]. We hypothesize that DNA instability caused by the observed accumulation of ROS in EtOH-stressed cells (Supplementary Figure S2G) caused the observed effects on autophagy and proteasome-related pathways (Figure 5A). Flow cytometry showed that all the stressed strains, especially LTs, exhibited increased percentages of cells with DNA damage (Supplementary Figure S2I; Supplementary Table S13; Supplementary Data S8). Only the X2180-1A and BY4742 strains under stress seemed to have inactive RAD53-PDS1 DNA repair mechanisms (Supplementary Figure S15; Supplementary Data S1). These transcriptomic data suggest that most strains might fix DNA defects through the cell cycle, although this was not observed via flow cytometry.

We examined whether lncRNAs were related to EtOH-stressed storage and degradation systems. The percentages of the 30 most relevant lncRNAs connecting synergistic pathways (within “storage” or “degradation”) were negatively related to the highest EtOH levels analyzed. Interestingly, a smaller number of lncRNAs connecting synergistic pathways improved EtOH tolerance: SEY6210 transcr_3536Δ was lethal, and BY4742 transcr_10027Δ reduced EtOH tolerance (Supplementary Figure S14; Supplementary Datas S7 and S9).

### 2.7. Overall EtOH Stress Data Integration: EtOH Stress Buffering Model

**Figure 6:**
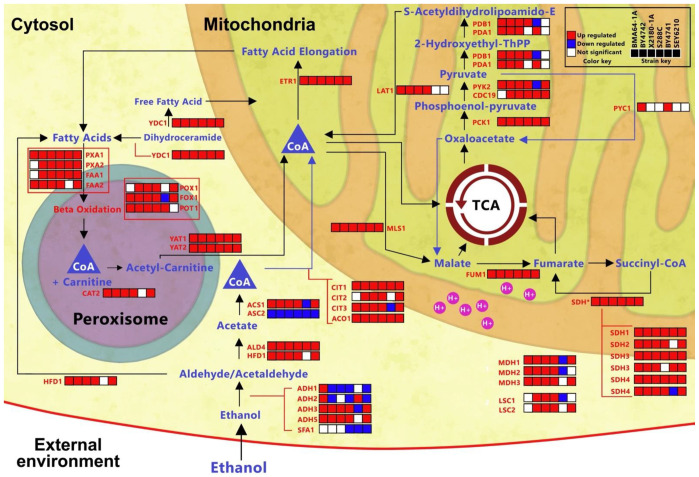
EtOH stress-buffering model based on the diauxic shift. The CoA indicates acetyl-CoA. This model was based on the integration of several datasets reported in Figure 3, Figure 4 and Figure 5, Table 2, Supplementary Tables S5, S6, S14 and S15, Supplementary Figures S2G–I, S5, S8, S10–S12 and S27.

We integrated our results to propose the “EtOH stress-buffering” model (Figure 6). The massive experiments and analyses conducted here showed that EtOH had a significant impact on energy and detoxification-related pathways. Most of the 41 genes crucial for the diauxic shift, the TCA cycle, and EtOH catabolism (Table 3) were upregulated, affecting the abundance of related metabolites (Figure 6; Supplementary Datas S1, S2, S4 and S6). Furthermore, the reduced glycerol efflux in many EtOH-challenged strains (Figure 5D) supported our model (discussed further below). This model will be scrutinized in Section 3.

The TCA cycle occurs in mitochondria synthesize ATP and produce ROS [2,49]. The TCA cycle is a core mechanism in our EtOH stress-buffering model because our transcriptome enrichment and network analysis revealed that the TCA cycle was one of the essential pathways affected by EtOH (Figure 4A). Moreover, most TCA cycle-related genes were upregulated in the analyzed strains, even after long-term exposure to EtOH (Figure 4B; Supplementary Data S6). Furthermore, oxidative stress-related genes were upregulated in the HTs (Supplementary Tables S14 and S15). Therefore, we measured succinate dehydrogenase (SDH) activity to assess mitochondrial activity in EtOH-challenged cells. The SDH activity assay revealed an increase in SDH activity in most EtOH-stressed strains (Supplementary Figure S2H; Supplementary Tables S3 and S13; Supplementary Data S8). Altogether, the data suggested higher mitochondrial activity in strains challenged with EtOH stress, consistent with the higher accumulation of ROS observed (Supplementary Figure S2G), providing better support for our model.

### 2.8. Peculiarity of the EtOH Stress-Buffering Model of the BMA64-1A Strain

**Figure 7:**
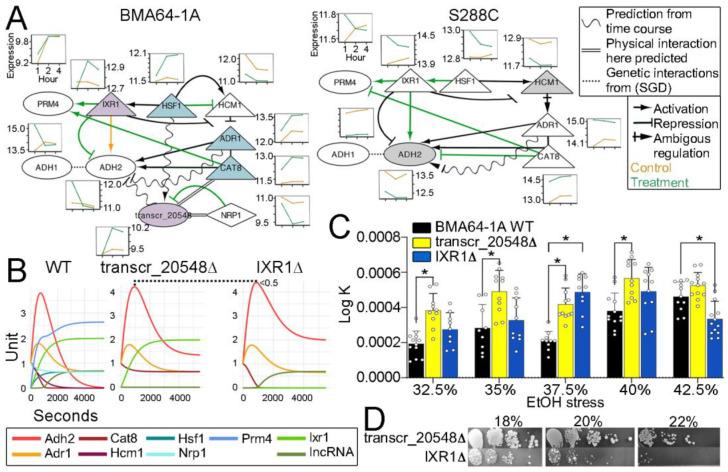
Subsystem model harboring the lncRNA transcr_20548, the diauxic shift, and EtOH stress-buffering genes. (**A**) BMA64-1A and S288C subnetworks. Small boxes represent the time-course data for each gene. Nodes with the same color have a similar time-course profile. These subnetworks were manually curated, including published data, information from the SGD database, and transcriptome time-course data from BMA64-1A and S288C. (**B**) BMA64-1A subnetwork dynamic network simulations of ordinary differential equations based on the transcriptome time-course of treatment conditions that simulate gene expression in virtual BMA64-1A WT and mutants. (**C**) Population rebound after stress relief in WT and BMA64-1A CRISPR–Cas9 mutants. * indicates a *p* value < 0.01. (**D**) EtOH tolerance spot test.

Our data showed a model peculiarity suggesting some level of strain specificity. Transcr_20548 of BMA64-1A interacts with many diauxic shift-related proteins included in our EtOH stress-buffering model (Figure 7A). We extracted this subnetwork and its counterpart from S288C to analyze the effect of transcr_20548 on our model; the S288C subnetwork did not include transcr_20548 because this lncRNA is specific to BMA64-1A. We initially inspected the transcriptome time-course data of genes in the BMA64-1A and S288C subnetworks.

In EtOH-stressed BMA64-1A, ADH2 expression decreased sharply and was correlated negatively with most genes (Figure 7A). Previous findings showed that Ixr1p, Adr1p and Cat8 repress ADH2 expression [50,51]. Here, IXR1 and transcr_20548 seemed to repress ADH2 expression in BMA64-1A under stress (Figure 7A). To verify which gene controls ADH2 repression, we simulated dynamic networks for the virtual BMA64-1A WT, transcr_20548Δ, and IXR1Δ subnetworks based on transcriptome time-course data (Supplementary Tables S16 and S17). We observed that ADH2 expression is not regulated by the joint actions of ADR1 and CAT8. ADH2 levels increased in both virtual mutants, although this increase was greater in the BMA64-1A IXR1Δ strain (inactive IXR1 and active transcr_20548) (Figure 7B). Both genes also repressed ADH2 (further discussed).

Furthermore, Adh2p is responsible for the reversible reaction of aldehyde/acetaldehyde to EtOH [52]. Thus, inactivation of transcr_20548 and IXR1 would reduce EtOH tolerance. Although mutant populations recovered faster than WT after EtOH stress relief, the transcr_20548Δ mutant outperformed IXR1Δ (Figure 7C). Both mutants exhibited reduced EtOH tolerance, although the effect was greater on IXR1Δ (inactive IXR1 and active transcr_20548) (Figure 7D). Finally, the simulations and experimental analysis of mutants corroborated our hypothesis that transcr_20548 is a stronger ADH2 repressor than IXR1. Moreover, both transcr_20548 and IXR seem to be the main ADH2 repressors.

Other relevant information in the BMA64-1A subnetwork includes the following: (1) IXR1 and HSF1 seem to repress ADH2 and HCM1 expression; (2) the NRP1-transcr_20548 interaction probably negatively affects the expression of transcr_20548; (3) HSF1 seems to positively regulate the expression of transcr_20548 (Figure 7A); and (4) the transcr_20548 *cis* elements contain HSF1-domain motifs (Supplementary Figure S28D). These data indicate substantial divergence between the BMA64-1A and S288C subnets.

### 2.9. Effect of EtOH on Lipid Metabolism

Lipid metabolism is a hot topic that has been broadly evaluated in yeast EtOH stress research. Here, we intended to verify whether lipid metabolism may be involved in the longevity of EtOH-stressed strains. Many genes related to lipid metabolism (e.g., ETR1, GPD1, MCR1, OPI3, FAA1, and GRE2) are induced under EtOH stress [5], as observed here (Supplementary Data S6). The metabolome and our KEGG mapping indicated a reduction in sphingolipid levels (mainly ceramides, KEGG sce00600) and sphingosine and IPC synthesis and increased levels of sphinganine, inositol 1-phosphate and squalene. Transcriptomic analysis revealed a reduction in the expression of AUR1 and an increase in the expression of the YDC1, PLC1 and ERG9 genes. Some of these genes and metabolites are involved in longevity (further discussed) (Supplementary Figure S16; (https://figshare.com/s/396119809b1916c07a60), accessed on 5 March 2023).

## 3. Discussion

We presented models and hypotheses for the mechanisms mediating EtOH tolerance and predicted the factors that may be responsible for establishing the two phenotypes defined here. We wish to highlight that the definitions of the HT and LT phenotypes presented here apply only to the context of this study. Thus, although the extrapolation of our models concerning HT and LT phenotypes is limited, these models may provide a basis for further studies.

The results from data integration allowed us to discuss four broader fields: (1) an overview of EtOH stress-responsive lncRNAs; (2) the essential pathways affected by EtOH stress; (3) the role of degradation/storage pathways in EtOH stress tolerance; and (4) the proposed EtOH stress-buffering model. The key points of each section are recorded in this video: https://figshare.com/s/81d0676a05ae08e5a775.

### 3.1. EtOH Stress-Responsive lncRNAs Are Functionally Diverse and Likely Involved in EtOH Tolerance

Despite interest in ncRNAs in *S. cerevisiae*, this report is the first to associate a large set of yeast lncRNAs with EtOH stress. A de novo set of lncRNAs was assembled using the transcriptome data from strains BMA64-1A, BY4742, SEY6210, BY4741, X2180-1A and S288C. According to a previous description of the characteristics of lncRNAs [14,26,29,30], our transcripts presented the essential characteristics of lncRNAs: lack of ORFs, low sequence and structural conservation, expression levels lower than those of coding genes and the expected number of interactions between ncRNAs and proteins for yeast.

As observed for most lncRNAs in this present study, yeast ncRNAs are often responsive to environmental changes [53], and stress response proteins are usually targeted by lncRNAs [54]. Our analysis of lncRNA–protein networks assigned lncRNAs to four broader functional categories: essential, membrane-dependent, metabolic and degradation processes. However, strain specificities were present. Below, we describe some directly-related EtOH stress-responsive lncRNAs evaluated in detail.

We predicted that transcr_20548 of BMA64-1A binds to IXR1. Previous findings showed that IXR1 repressed ADH2 expression in yeast undergoing glucose starvation [50,51]. However, our network simulations and mutant analysis showed that transcr_20548 is a stronger ADH2 repressor than IXR1 in EtOH-stressed and glucose-starved BMA64-1A cells. We suggest that the mechanism of ADH2 inhibition by transcr_20548 is dependent on Nrp1p. Nrp1p is a putative RNA-binding protein [55] located in SG under glucose starvation [42]. We observed that transcr_20548 interacts with Nrp1p in BMA64-1A (Figure 7A), and the two genes diverge in expression profile. Our analysis suggests that EtOH-induced SGs may not have Nrp1p that could trap most of the transcr_20548 overexpressed after 2 h of stress. Thus, transcr_20548 may be free to inhibit ADH2 expression.

We previously showed that lnc9136 (transcr_9136) in SEY6210 induces a skip in mitotic arrest, while lnc10883 (transcr_10883) in BY4742 avoids DNA and spindle damage checkpoints in EtOH-stressed cells [32].

### 3.2. EtOH Causes Extensive Rewiring of Life-Essential Pathways: Longevity, Peroxisome, CTA1 and SUI2 Are Master Key Regulators of EtOH Tolerance Phenotypes

Although several articles have shown that life-essential pathways are affected by EtOH tolerance/stress in yeast [5,7,18,19,20,21,22,23,24,25], here, we propose systems mechanisms that trigger the HT and LT EtOH tolerance phenotypes defined here, mainly analyzing networks and the BMA64-1A strain.

According to graph theory [56,57,58], the network changes observed here redirect system signals, causing delays in reaching specialized pathways and favoring basal system/process activation: the theoretical basis for this conclusion is described in the Supplementary Text (“Results”, topic “3.4”). Therefore, we suggest that cells exposed to EtOH stress favor the activation of basal processes to preserve and prepare cells for stress relief. We ranked MAPK, longevity, autophagy, peroxisome, TCA cycle, glycolysis and gluconeogenesis (Gly/gluc), protein processes in the endoplasmic reticulum, and RNA- and ribosomal-related organelles, processes and pathways as the basal processes extensively affected by EtOH.

Signal transduction is essential for the EtOH response [21]. Peroxisomes influence longevity [59], and longevity increases the lifespan of stressed cells [60]. The CTA1 gene acts on EtOH tolerance and seems to degrade ROS [61,62]. We found that the CTA1 gene transmits MAPK signals to the longevity and peroxisomal pathways. BMA64-1A activates CTA1 and exhibits the highest ROS accumulation during stress. The relevance of CTA1 is evidenced by the reduced tolerance to EtOH in BMA64-1A CTA1Δ. Altogether, our findings suggest that MAPK signaling-mediated CTA1 induces EtOH tolerance-related phenotypes soon afterward. Furthermore, the balance between ROS and CTA1 accumulation seems to be involved in this process.

We observed that SUI2 transmits signals from autophagy to ribosomal and RNA-related pathways. SUI2 participates in the arrest of mRNA transcription in response to stress [37]. The SUI2 gene presented a repressive expression profile in BMA64-1A, which could be related to the significant reduction in RNA yield observed in this strain under stress. Although our analyses are still insufficient, to the best of our knowledge, these are the first reported results concerning the relationships among RNA yield, SUI2 and EtOH stress.

Spermidine and lipids also seem to affect the longevity of EtOH-stressed strains. We suggest that the joint action of spermidine and SGF29 expression triggers the positive effect of spermidine on the growth of BMA64-1A. Furthermore, only HT strains accumulate squalene and sphinganine, which could be responsible for exacerbating the negative effects on cell viability in this phenotype. For a detailed discussion, see the first paragraphs of “Supplementary Text”, section “Discussion”, topic “2 and 5”.

### 3.3. Membraneless Organelles, Storage, and Degradation Systems Are Related to EtOH Stress: lncRNAs Act on These Systems

Previous findings revealed that severe EtOH stress in yeast induces the formation of PB and SG [40]. All cell attributes that allow the formation of PB and SG [38,39,40,41,42,43] were observed in our experiments, including the early stationary growth phase, fast population rebound, cytosol acidification, glucose starvation and ROS accumulation. In summary, we suggest that the storage and degradation systems (PB, SG, PSG, RNA and protein catabolism) act as surveillance strategies in EtOH-stressed cells until stress relief occurs, as these structures were active in most of the strains analyzed. ROS may cause DNA damage [2]. Previous findings showed that DNA damage induces defects in RNAs and proteins [47] and that EtOH stress induces protein unfolding [63]. Our experimental analysis of ROS and DNA damage accumulation showed that EtOH-stressed strains accumulated DNA damage, likely via ROS accumulation. However, several analyses performed here (omics, network, growth curve and Western blot analyses) evidenced the presence of membraneless structures and pathways responsible for RNA and protein catabolism. Therefore, we suggest that defective RNAs and proteins produced under high EtOH stress may be degraded by different mechanisms activated in our experiments (e.g., PB, SG (via translation stalling), PSG, autophagy and proteasomal pathways).

In fact, several mechanisms act to degrade irregular molecules, eventually promoting lifespan under stress, stationary phase growth or glucose starvation: (1) PB degrades abnormal mRNAs and controls translation [38,41,43,64,65,66,67,68,69]; (2) autophagy degrades damaged proteins [34]; (3) irregular proteins may be degraded by the ubiquitin–proteasome pathway [70]; (4) SG improves cell viability during stress by inhibiting translation-related proteins; and (5) SG sequesters capped and poly(A) mRNAs, stalling translation and avoiding their degradation by PB [38,70,71].

LncRNAs can scaffold RNAs and proteins in membraneless structures [38,72]. We identified several lncRNAs that likely bind to storage-related (SG, PSG and PDNR) and degradation-related (PB, proteasome and PPPR) proteins to assess whether lncRNAs were involved in these processes (Supplementary Figure S14A). The statistical analysis of the percentage of lncRNAs in each strain that bound to these proteins suggested a smaller number of lncRNAs connecting to synergic pathways (within storage or degradation) promoting a higher EtOH tolerance. However, the results for the CRISPR–Cas9 mutants refuted our hypothesis since neither mutant improved EtOH tolerance. Thus, the theoretical relationship between EtOH stress-responsive lncRNAs and linked synergistic pathways was not clarified here.

However, our findings suggest that strains under the maximum EtOH stress level analyzed here underwent DNA damage triggered by ROS accumulation, culminating in defects in RNAs and proteins. However, cells cope with these molecules through different mechanisms, mainly by using membraneless organelles. The processes mentioned above may be an additional cell surveillance strategy to cope with this stress in addition to inhibiting specialized subsystems, as already discussed above. Based on the study of BMA64-1A, HTs seem to take advantage of this ‘detoxification’ process. Finally, although we are presenting a general model, the presence of lncRNAs involved in the processes mentioned suggests some level of strain specificity in the strategy used to cope with damaged molecules produced under EtOH stress.

### 3.4. EtOH Stress-Buffering Model

*S. cerevisiae* can use accumulated EtOH as a substrate for aerobic respiration [3], consistent with the general idea of our “EtOH stress-buffering” model (Figure 6); all strains were cultivated here under aerobic conditions. The core of our EtOH stress-buffering model is the diauxic shift and TCA cycle. Overall, our model suggests that chronic EtOH stress induces a diauxic shift by consuming EtOH and glycerol. This process intensifies acetyl-CoA, fatty acid, TCA cycle and peroxisome activities, resulting in increased energy production to buffer intense stress. Below, we present results from the literature and their correlation with our findings to support and verify our model. We only included the details of the reactions and functions of genes and metabolites in the context of our EtOH stress-buffering model (Table 3; Figure 6) because most other steps are common TCA cycle reactions. Therefore, details of the TCA cycle are provided in the Supplementary Text (“Discussion”, topic “4”).

EtOH is a relevant carbon source in yeast cells. For instance, the diauxic shift restores aerobic growth in glucose-depleted cells by using EtOH as the main carbon source. Moreover, the TCA cycle uses carbon from external EtOH [1].

Alcohol dehydrogenases convert absorbed EtOH to acetaldehyde, providing the majority of the acetyl yield in yeast [1,73]. Here, we observed glucose starvation in EtOH-stressed strains and the upregulation of diauxic shift-responsive genes, genes essential for growth with nonfermentable carbon sources, and alcohol dehydrogenases. These data indicate the activation of the diauxic shift and conversion from EtOH to acetaldehyde/aldehyde in the stressed strains. The alcohol dehydrogenase SFA1 is downregulated only in LT strains, indicating that HT takes advantage of EtOH catabolism. Acetaldehyde or aldehyde is then converted to acetyl-CoA in the cytosol and peroxisomes: genes involved in this metabolism were upregulated in almost all the strains analyzed here.

Acetyl-CoA and peroxisomal acetyl-carnitine are transferred to mitochondria and converted to fatty acids [74,75]. Fatty acids are transferred to peroxisomes [76] and are converted back to acetyl-CoA (Figure 6). Most mitochondrial acetyl-CoA is derived from the cytosol, and most of the carbon in fatty acids is derived from external EtOH [1]. Here, the ETR1, PXA1 and PXA2 genes (encoding enzymes relevant to the mentioned pathway) were upregulated in all the strains analyzed, and carnitine metabolism was enriched in the HT transcriptome. Therefore, in our EtOH stress-buffering model, we suggest that mitochondrial acetyl-CoA seems to pass through a positive feedback loop for synthesis regardless of the phenotype. Remarkably, strain specificities may be present in this part of the model, since lncRNAs work with specific enzymes: although Yat2p putatively binds to lncRNAs in BY4742, SEY6210 and BY4741, the effects of these interactions are unknown.

The TCA cycle uses acetyl-CoA to generate energy [49]. Our data suggested higher TCA cycle activation in all EtOH-stressed strains: (1) genes in this pathway were upregulated and enriched in almost all strains (Supplementary Data S6; Supplementary Table S6); (2) this pathway was the only one to present an activation transcriptome time-course profile in both BMA64-1A and S288C strains (Figure 4B); (3) SDH activity was enhanced in almost all analyzed strains (Supplementary Figure S2H); and (4) the level of the malate metabolite (essential for the TCA cycle) was increased in both phenotypes (Supplementary Data S4).

A higher energy yield from the TCA cycle helps cells endure EtOH stress [18]. In the TCA cycle, acetyl-CoA condenses with oxaloacetate to form citrate [49]. Oxaloacetate is a potent SDH inhibitor [77]. The SDH enzyme converts succinate to fumarate and pumps H^+^ to the mitochondrial intermembrane space, promoting ATP synthesis. Here, the observed reduction in oxaloacetate in only EtOH-stressed HTs (Supplementary Data S4) suggests higher SDH activity in cells of this phenotype, as observed in Supplementary Table S13. Furthermore, the observed reduction in fumarate in HTs (Supplementary Data S4) suggests the occurrence of increased H^+^ pumping instead of increased TCA cycle activity. The likely increase in ATP synthesis in HTs may promote the increase in their capacity to cope with EtOH stress; in this case, HTs present higher EtOH buffering.

EtOH is the predominant source of NADH and NADPH when glycolysis is impaired by oxidative stress [1], supporting our model showing that the higher EtOH level to which HT strains are exposed may elevate H^+^ pumping, energy production and EtOH buffering; HT strains also present higher ROS accumulation than LT strains under EtOH stress (Supplementary Table S13).

## 4. Materials and Methods

The Supplementary Text (section “Methods”) contains substantial details regarding experiments, data collection, analyses, equipment, chemicals, equations, schemes and rationales. The material mentioned above includes all pertinent information required to ensure the reproducibility of the data. Thus, in the following, we present the essential points regarding the materials and methods used here.

### 4.1. Defining the Highest EtOH Tolerance

Each strain (Supplementary Table S1) in late log-phase (~12 h of cultivation) was harvested, resuspended in different concentrations of EtOH or physiological solution (treatment and control conditions, respectively) and incubated (30 °C, 1 h, 120 RPM). The samples were streaked on YPD plates, and a visual inspection of colony formation allowed us to establish the highest EtOH tolerance level supported (Figure 1A).

Clustering of the EtOH tolerance data matrix classified strains into either the HT or LT phenotype. Three LT strains (SEY6210, BY4741 and S288C) and three HT strains (BMA64-1A, BY4742 and X2180-1A) were selected for further analysis (Supplementary Table S1; Figure 1A) by comparing the control vs. treatment (the highest EtOH level tolerated for each strain). Specific comparisons or other EtOH levels tested are indicated and detailed in the Supplementary Text (section “Methods”).

### 4.2. Cell Biology Analysis

The 1st and 2nd growth experiments assessed the population growth under the best conditions and the population rebound after stress relief (the growth recovery rate after severe stress), respectively. The 3rd experiment was performed in YPD medium supplemented with the highest tolerated level of EtOH for each strain to assess stress severity. The 4th experiment assessed whether the spermidine level affected growth by cultivating the strains under their highest tolerated EtOH stress level with and without spermidine supplementation. The 5th experiment was similar to the 3rd experiment for comparative assessments with the results from the 4th experiment. The 6th experiment was performed to determine the association between proton efflux and growth under EtOH stress. The goal of the 7th experiment was to analyze the population rebound after stress relief of mutants.

Flow cytometry was used to estimate the effect of EtOH on the number of cells with DNA damage, ROS levels, and cell viability. Mitochondrial activity was assessed by estimating succinate dehydrogenase (SDH) activity. RNA yield was quantified by microscopy after staining cells with acridine orange and DAPI. High-performance liquid chromatography (HPLC) was used to determine the D-glucose and glycerol levels in the medium [78]: the difference between 1 h and 0 h was calculated for the control and treatment conditions. We performed Western blotting using specific antibodies to investigate the effect of EtOH stress on mRNA surveillance, the formation of PB and SG, and translation stalling by SG.

### 4.3. Acquisition of Omics Data

Transcriptomic, proteomic and metabolomic data were obtained for all 6 selected strains, and time-course (2 h and 4 h) transcriptomic data were obtained for BMA64-1A and S288C cells under control and treatment conditions. The genome of strain BMA64-1A was also sequenced. Illumina sequencing (NCBI BioProject number PRJNA727478), LC–MS/MS and GC–MS/MS were used to acquire transcriptomic/genomic, proteomic and metabolomic data, respectively. The complete set of procedures for genomic DNA, total RNA, protein and metabolite extraction and additional procedures are detailed in Sections “4.1. Raw omics data obtainment” and “4.2. Biomolecule extraction” in the Supplementary Text.

### 4.4. Bioinformatics

#### 4.4.1. Omics Analysis

The detailed bioinformatics analyses applied to our omics data, including software, parameters, strategies, filtering process, statistics and other procedures, are described in detail in Section “5. Bioinformatics” in the Supplementary Text.

Overall, the genome assembly of the BMA64-1A strain was performed using 5 different assemblers with varying parameters and assembly strategies. The best assembly was chosen using statistics and a tool that compares assembly metrics (Supplementary Data S10). Genome annotation was performed using two different protocols, one of which was performed by the genome annotation team of the Saccharomyces Genome Database (SGD).

The in-house pipeline to find the lncRNAs used many bioinformatics tools, algorithms and approaches (Supplementary Figures S17–S20; Supplementary Table S20; Supplementary Datas S11–S13). Overall, quality-filtered RNA-Seq reads of noncoding sequences were identified and assembled for each strain using the “Single Assembler Multiple Parameters” strategy [79]. The lncRNAs were selected by excluding spurious and coding assembled transcripts. LncRNA-encoded micropeptides were searched against our proteomics data using the Trans-Proteomics pipeline. The identified lncRNAs were compared using BLASTn to 3898 ncRNAs (including CUTs, SUTs and XUTs) of yeast previously reported in more than 20 papers (https://figshare.com/s/9689d0046c824d3e1f74, accessed on 5 March 2023).

Repetitive DNA and tRNAs were searched and annotated for all strains using RepeatMasker and tRNAscan-SE (Supplementary Figure S21; Supplementary Data S14). Transposable element annotations were manually curated. Finally, the updated genomic coordinates of each strain (https://figshare.com/s/e75fe1be9af623988be1, accessed on 5 March 2023) were used for differential expression (DE) analysis (Supplementary Figures S22 and S23; Supplementary Table S21).

Quality-filtered RNA-Seq reads from each strain were mapped onto their respective genomes before identifying the differentially expressed genes (DEGs) by comparing the treatment and control groups. After the mass/charge conversion to peptides, spectral counting was used to quantify the proteins. The differentially abundant proteins (DAPs) were assessed by comparing the treatment and control conditions. Baseline correction, peak detection, retention time alignment and library matching were performed for the metabolome. The selected metabolites were matched against the GMD database. The differentially abundant metabolites (DAMs) of each strain were determined by comparing the treatment vs. control (Supplementary Figure S24). We defined phenotype-specific DAMs by selecting those present in at least two HT strains or two LT strains. Enriched gene ontology terms for DEGs shared between phenotypes or phenotype-exclusive DEGs were independently analyzed. Similarly, we also analyzed the enriched KEGG pathways for both DEGs and DAMs.

#### 4.4.2. Analysis of the lncRNAs and Networks

The lncRNA–protein interaction networks (referred to as LNCPI) were computationally predicted for each strain and filtered by interaction probability (≥0.95). Then, significant DEGs and DAMs from each strain and strain-specific lncRNA–protein interactions were mapped to KEGG pathways.

Structural conservation among lncRNAs was assessed based on the hypothesis that lncRNAs that bind to the same proteins show either intra- or interstrain conservation. Proteins targeted by many lncRNAs in BMA64-1A (HT model) and S288C (LT model) cells were selected (Supplementary Table S18). Intrastrain sequence similarities among the lncRNAs that likely bind to those proteins were sought. The secondary structures of the two most similar lncRNAs per protein were predicted and compared within strains. We compared the structures of the lncRNA orthologs to transcr_22854, transcr_24032 and transcr_20180 of S288C for the interstrain comparison.

The guilt-by-association approach was used to assign lncRNA functions summarizing the gene ontology terms for their target proteins. Thus, three independent assignments were performed: based on all lncRNA target proteins, using proteins from DEGs shared between phenotypes, or considering proteins from phenotype-exclusive DEGs.

The diffusion algorithm [31] using network information flow starting from each DE lncRNA (lncRNA propagation analysis) generated subgraphs of each strain: the up- and downregulated sequences were independently analyzed. These data showed key lncRNAs, how lncRNAs affect EtOH tolerance, and whether EtOH triggers similar pathways, regardless of strain-specific lncRNAs and interactions.

Our omics data revealed systems strongly affected by EtOH stress: autophagy, PB, RNA catabolic processes, SG, PSG, proteasome and regulatory parts, protein polyubiquitination and positive regulation of ubiquitination (PPPR), and protein deubiquitination and negative regulation of ubiquitination (discussed further below). The expression profiles of these system-related genes and lncRNAs were evaluated. The number of lncRNAs connecting the synergistic systems (structures or pathways that had similar outcomes, e.g., the proteasome and PPPR, which act on protein degradation) was calculated. The data showed the relationship between the number of lncRNAs in those systems, EtOH tolerance and population rebound.

The lncRNA transcr_20548 in BMA64-1A is an EtOH stress-responsive lncRNA that directly influences EtOH tolerance (discussed further below). A subnetwork harboring this lncRNA was extracted and refined from the BMA64-1A LNCPI to better understand the role of transcr_20548. Systems dynamic modeling was performed to assess how the genes in this subsystem affect the transcription of the ADH2 gene.

Basal systems that likely trigger divergences between strains S288C and BMA64-1A were sought. We generated a pathway integrated network (PINET) by integrating the glycolysis and gluconeogenesis (Gly/gluc), TCA cycle, peroxisome, cell cycle, autophagy, MAPK, longevity, protein process in endoplasmic reticulum (PPER), RNA transport, ribosome biogenesis, mRNA surveillance and RNA degradation pathways. Then, a single network per phenotype (represented by BMA64-1A and S288C as models) was created using TiCoNE [35] employing the time-course data. The largest network cluster of each phenotype-PINET was compared. The time-course expression profile within each phenotype-PINET allowed us to set each pathway as activation (“up and stable”), deactivation (“down and stable”), or “stable” profiles. Finally, we modeled the DNA damage pathway using information from the literature and time-course data to determine the possible mechanism of DNA damage during EtOH treatment.

Protein–protein interactions, gene regulatory networks, and metabolic networks available for yeast were filtered and unified. The integrated network was merged with each LNCPI, providing 6 strain-specific networks. Two additional networks were created using the DE data to represent the control and treatment from each mentioned network. Finally, statistics were used to compare topological features among the 16 graphs (Supplementary Figure S25).

#### 4.4.3. Mutant Generation and Analysis

Mutants with inactivation of lncRNAs (transcr_20548 in BMA64-1A, transcr_10027 in BY4742, and transcr_3536 in SEY6210) and coding genes (CTA1 and IXR1 in BMA64-1A) were generated using CRISPR–Cas9. The genomic target regions were inserted into the pMEL16 plasmid (Addgene 107922) via PCR. Competent yeast cells were transformed with the modified pMEL16 plasmid, p414-TEF1p-Cas9-CYC1t plasmid (Addgene 43802), and the repair DNA. Recovered cells were placed on drop-out His- plus G418 medium, followed by incubation. Mutants were confirmed by colony PCR and sequencing. The population rebound after EtOH stress relief was assessed for mutants and wild-type strains, and spot tests were performed to assess the highest EtOH tolerance level of each mutant (Supplementary Table S19).

## 5. Conclusions

The largest data integration study of EtOH-tolerant yeast phenotypes is shown here. Overall, the massive data integration allowed us to develop models to explain EtOH tolerance, including the lncRNAs in this context. These models include modification of life-essential pathways, degradation and storage pathways, and our EtOH stress-buffering model. Moreover, although the EtOH tolerance phenotype relies on the activation of many strain-specific mechanisms, general patterns were identified to discriminate the HT and LT phenotypes analyzed here.

The key features of our findings are as follows: (1) EtOH stress-responsive lncRNAs can act in a strain-specific manner to overcome EtOH stress; (2) EtOH-stressed cells retain mainly the life-essential pathways to preserve and prepare themselves for stress relief. Longevity, peroxisome, and CTA1 are the first triggers of EtOH phenotypes; (3) the degradation and storage pathways help cells withstand the harm caused by EtOH stress, and HTs takes advantage of these processes; and (4) the diauxic shift drives an EtOH buffering mechanism, prompting an energy burst to hinder stress (higher boosting, higher tolerance) (https://figshare.com/s/81d0676a05ae08e5a775, accessed on 5 March 2023).

In addition to the basic knowledge concerning the systemic response to EtOH, an intrinsic cell stressor, this work may also be helpful for biotechnology purposes.

## Figures and Tables

**Table 1:** Strain description, cell viability, and SDH assays. 1: Strain subjected to genome sequencing (Supplementary Tables S1 and S2). 2: Strains subjected to the time-course experiment. Although BMA64-1B (tolerates 26% EtOH) and CEN.PK2-1C (tolerates 20% EtOH) were also used in phenotype classification, they were excluded from further analysis to minimize repeated supported EtOH concentrations.

Strain or Group	EtOH Tol. (%)	Phenotype	Genotype
BMA64-1A ^1,2^	30	HT	MATa; his3-11_15; leu2-3_112; ura3-1; trp1Δ2; ade2-1; can1-100
BY4742	26	HT	MATα; his3Δ1; leu2Δ0; lys2Δ0; ura3Δ0
X2180-1A	24	HT	MATa SUC2 mel gal2 CUP1
BY4741	22	LT	MATa; his3Δ1; leu2Δ0; lys2Δ0; ura3Δ0
SEY6210	20	LT	MATα suc2-Δ9 ura3-52 leu2-3112 his3-Δ200 trp1-Δ901 lys2-801
S288C ^2^	20	LT	MATα SUC2 mal mel gal2CUP1

**Table 2:** General functions of selected EtOH stress-responsive lncRNAs. *, functions assigned by additional analysis or experiments further described. **, lncRNAs that function in the cell cycle [32].

Strain	LncRNAs	Putative Main Functions
BMA64-1A	transcr_6448, transcr_20548 *	Branched-chain alcohol tolerance metabolism, regulation of the response to EtOH, and stress granules
BY4742	transcr_10883 **, transcr_10027 *, transcr_9158 *, transcr_7869 *, transcr_63478	Degradation, metabolic pathways, cell signaling, division, cell wall, transport, transcription, replication, ribosome biogenesis, and storage/degradation pathways
X2180-1A	transcr_3746, transcr_6988, transcr_8290	Degradation, membrane-dependent process, cell wall, cell surveillance, longevity, growth, and transcription
BY4741	transcr_3338, transcr_2916	Membrane-dependent processes
SEY6210	transcr_8157, transcr_3536 *, transcr_9136 **	Membrane-dependent processes, diauxic shift, cell cycle, storage/degradation pathways
S288C	transcr_18666, transcr_18820, transcr_21244, transcr_19266, transcr_6225	Degradation, trehalose metabolism, and ribosomal biogenesis

**Table 3:** Genes related to the EtOH stress-buffering model. *: Citations and description of genes and metabolites are available in the “Supplementary Text” (“Discussion”, topic “4.”).

Analyzed Genes and Metabolites	Relevant Action in the EtOH Stress-Buffering Model	Source
ADR1, CAT8, GUT1, GUT2, INO4, NQM1 and RSF1	Essential for growth on nonfermentable carbon sources and diauxic shift-responsive genes	*
ALD4, ACS1, FAA1, FAA2, FOX2, HFD1, PXA1, PXA2, POX1, and POT1	Essential to metabolize acetyl-CoA in the cytosol and peroxisomes	*
CAT2, YAT1, and YAT2	Carnitine acetyltransferase genes	SGD database
CIT2, FUM1, GDH2, GDH3, KGD1, KGD2, LAT1, LSC1, LSC2, MDH1, MDH2, MDH3, MLS1, PCK1, PDA1, PDC1, and PYC1	TCA cycle-related genes	*; SGD database; KEGG sce00010
ADH3, ADH5, and SFA1	Alcohol dehydrogenase may catabolize EtOH to create acetaldehyde/acetate	*; KEGG sce00010; YeastPathways EC Number 1.1.1.1; [1]
ETR1	Acetyl-CoA catabolism in the fatty acid elongation metabolism	*
Oxaloacetate, fumarate, and malate	TCA cycle-related metabolites	*

## Data Availability

Supplementary Data are available at BioProject number PRJNA727478, https://figshare.com/s/e524aabebcf5dfe0a106, accessed on 5 March 2023, https://figshare.com/s/e75fe1be9af623988be1, accessed on 5 March 2023, https://figshare.com/s/396119809b1916c07a60, accessed on 5 March 2023, https://figshare.com/s/4b04ae15e5ab4f1b73c5, accessed on 5 March 2023, https://figshare.com/s/9689d0046c824d3e1f74, accessed on 5 March 2023, https://figshare.com/s/537bb28192e48b8483c5, accessed on 5 March 2023, https://figshare.com/s/0ef043d4cd1d8b1f0bb1, accessed on 5 March 2023, and https://figshare.com/s/81d0676a05ae08e5a775, accessed on 5 March 2023.

## Supplementary Materials

The following supporting information can be downloaded at: https://www.mdpi.com/article/10.3390/ijms24065646/s1.
