# Supplementary material for: Integrative Analysis of the Ethanol Tolerance of Saccharomyces cerevisiae
Source: Int J Mol Sci. 2023 Mar 15;24(6):5646. doi: 10.3390/ijms24065646 (PMC10051466; doi:10.3390/ijms24065646)
Supplement: Supplementary file 1 [file ijms-24-05646-s001.zip › Supplementary_Text_2.pdf]

Supplementary Text

Here we present detailed material and methods, additional results, and additional discussion. Supplementary figures and short tables are also presented. The large tables were inserted into tabs in the companion Excel file called **Supplementary Data**.

Contents

SUPPLEMENTARY TEXT 1

LIST OF ABBREVIATIONS 3

LIST OF FIGURES 5

LIST OF TABLES 6

SUPPLEMENTARY FIGURES 7

SUPPLEMENTARY TABLES 30

METHODS 41

1. Experimental designing and analysis overview 41
2. The ethanol tolerance experiments 41
3. Cell biology analysis 42
   3.1. Growth curves *42*
   3.2. Succinate dehydrogenase assay, flow cytometry analysis, RNA and glycerol yield measurements, glucose influx, and western blot *44*
      3.2.1. Succinate dehydrogenase (SDH) activity assay 44
      3.2.2. Flow cytometry to measure DNA damage, ROS, and cellular viability 45
      3.2.3. RNA yield evaluation 46
      3.2.4. Glucose uptake and glycerol yield 47
      3.2.5. Western blot 47
4. OMICs 48
   4.1. Raw OMICs data obtainment *48*
   4.2. Biomolecule extraction *49*
5. Bioinformatics 50
   5.1. Genome assembling and annotation of BMA64-1A *50*
   5.2. Proteomics analysis *51*
   5.3. Metabolomics analysis *52*
   5.4. Re-editing the annotations before differential gene expression analysis *53*
      5.4.1. Transposable elements and tRNA identification 53
      5.4.2. The in house pipeline to identify the lncRNAs and comparative analysis 54
   5.5. Differential gene expression, GO enrichment and KEGG pathways mapping *58*
   5.6. Networks analysis *59*
      5.6.1. Modeling the KEGG pathway-based networks based on time-course data 59
      5.6.2. Integrating lncRNAs into networks, statistical analysis of networks, and network dynamic modeling 60
6. Mutants generation 63

RESULTS 66

1. Defining phenotypes, and cell growth analysis 66
2. Genome and lncRNA assembly, and annotation update 66
3. Quantitative analysis of transcriptome, proteome, metabolome and networks 67
   3.1. Differential expression: transcriptome *67*
   3.2. Differential abundances: Proteomics *68*
   3.3. Differential abundances: Metabolomics *68*
   3.4. Topological metrics of integrated networks *68*
4. Qualitative analysis (functional description) of transcriptome, proteome, metabolome and networks 69
   4.1. DEGs previously reported as responsive to other stressors *69*
   4.2. Putative function of lncRNAs and structural analysis *70*
   4.3. Narrowing down the pathways affected by the EtOH stress *71*
      4.3.1. Overview of GO enrichment analysis 71

4.3.2. General metabolic pathways affected by the EtOH 71
4.3.3. Basal pathways affected by EtOH 72
4.3.4. Diauxic shift mechanism mediates the ethanol buffering 73
4.3.5. Acidification assay, and membraneless structures affected by the EtOH stress 74
5. Cell biology analysis 75
5.1. Cell viability, ROS, SDH, DNA damage assays, RNA yield, glucose influx, and western blot........ 75

DISCUSSION........ 76

1. EtOH stress responsive lncRNAs are functionally diverse and likely involved in EtOH tolerance 78
2. EtOH causes extensive rewiring of life-essential pathways: longevity, peroxisome, and CTA1 are master key regulators of EtOH tolerance phenotypes........ 83
3. Membraneless organelles, storage, and degradation systems are related to EtOH stress: lncRNAs also act on these systems........ 85
4. The EtOH stress-buffering model 85
5. Other interesting genes and mechanisms likely affected by EtOH stress........ 87

SUPPLEMENTARY REFERENCES 88

List of abbreviations

Anaphase Promoting Complex = APC
BC = Burt Constraint
CAFI = Condensed-to-Atoms Fukui Indexes
CAT = Carnitine Acetyltransferases
CDS = Coding Sequence
CUT = Cryptic Unstable Transcript
DAM = Differentially Abundant Metabolite
DAP = Differentially Abundant Proteins
DE = Differentially Expressed
DEG = Differentially Expressed Gene
DFT = Density Functional Theory
DGE = Differential Gene Expression analysis
ER = Endoplasmic Reticulum
FC = Fold-Change
FDR = False Discovery Rate
FFA = Free Fatty Acid
GPI = Glycosylphosphatidylinositol-Anchored Proteins
GO = Gene Ontology
HPS = Heat Shock Proteins
HDR = Homologous Directed Repair
HT = Higher Tolerant
IPC = Inositol PhosphorylCeramide
LL = viable cells (AnnexinV-/PI-)
LNCPI = lncRNA-protein interaction network
LR = cells under initial apoptosis (AnnexinV+/PI-)
LT = Lower Tolerant
LTR = Long Terminal Repeat
L2FC = Log2 Fold-Change
nt = nucleotides
OD_{600} = Optical density measured at a wavelength of 600 nm
ODE = Ordinary Differential Equation
PB = P-body
PCA = Principal Component Analysis
PDNR = Protein Deubiquitination and Negative Regulation of Ubiquitination
PE = Phospho-Ethanolamine
PINET = Pathway Integrated NETwork
PLS-DA = Partial Least Squares Discriminant Analysis
PPER = Protein Process in Endoplasmic Reticulum
PPPR = Protein Polyubiquitination and Positive Regulation of Ubiquitination
PSG = Proteasome Storage Granules
RNAPI = RNA polymerase I
RNAPII = RNA polymerase II
RNAPIII = RNA polymerase III
ROS = Reactive Oxygen Species
SDH = Succinate Dehydrogenase
SG = Stress Granules
SGD = Saccharomyces Genome Database
snoRNP = small nucleolar ribonucleoprotein
TCA = Tricarboxylic Acid Cycle
TE = Transposable Elements

UL = cells in advanced death (Annexin-/PI+)
UR = cells in advanced death (Annexin+/PI+)
SUT = Stable Annotated Transcript
YMDB = Yeast Metabolome Database

List of Figures

SUPPLEMENTARY FIGURE 1: THE GROWTH CURVE ANALYSIS FOR THE 6 SELECTED STRAINS. 7
SUPPLEMENTARY FIGURE 2: CELL VIABILITY, SDH, ROS AND DNA DAMAGE QUANTIFICATION. 8
SUPPLEMENTARY FIGURE 3: SET ANALYSIS OF DIFFERENTIALLY EXPRESSED CODING GENES. 9
SUPPLEMENTARY FIGURE 4: DIFFERENTIALLY ABUNDANT PROTEINS (DAPS) BETWEEN PHENOTYPES. 10
SUPPLEMENTARY FIGURE 5: DIFFERENTIALLY ABUNDANT METABOLITES (DAMS) COMPARING STRAINS AND PHENOTYPES. 10
SUPPLEMENTARY FIGURE 6: OVERVIEW OF LNCRNA EXPRESSION AND SECONDARY STRUCTURE COMPARISONS OF LNCRNAS. 11
SUPPLEMENTARY FIGURE 7: LENGTH OF LNCRNAS AND ORTHOLOGUES MATCHING. 12
SUPPLEMENTARY FIGURE 8: GO ENRICHMENT ANALYSIS OF LNCRNAS USING G:CONVERT. 13
SUPPLEMENTARY FIGURE 9: THE NETWORK DEGREE DISTRIBUTION. 14
SUPPLEMENTARY FIGURE 10: COMMUNITIES OF PATHWAY INTEGRATED NETWORK (PINET) AND THE REWIRING AFTER ETOH STRESS INTEGRATING THE TIME-COURSE. 15
SUPPLEMENTARY FIGURE 11: EXPRESSION PROFILE OF GENES LINKED TO PATHWAYS (*E.G.*, "CELL CYCLE"). 16
SUPPLEMENTARY FIGURE 12: NETWORK PROPAGATION (SYSTEMS SIGNALING ANALYSIS) FROM EACH PATHWAY BASED ON THE PATHWAY INTEGRATED NETWORK (PINET). 17
SUPPLEMENTARY FIGURE 13: RNA YIELD QUANTIFICATION BY ACRIDINE ORANGE ASSAY. 17
SUPPLEMENTARY FIGURE 14: ANALYSIS OF GENES AND LNCRNAS RELATED TO MEMBRANELESS STRUCTURES OR DEGRADATION/STORAGE PATHWAYS RESPONSIVE TO THE ETOH STRESS. 18
SUPPLEMENTARY FIGURE 15: MODEL OF RAD53 AND PDS1 MECHANISMS OF DNA CHECKPOINT REGULATION. 19
SUPPLEMENTARY FIGURE 16: SOME KEGG METABOLIC PATHWAYS ANALYZED. 20
SUPPLEMENTARY FIGURE 17: PIPELINE TO ASSEMBLY THE LNCRNAS. 21
SUPPLEMENTARY FIGURE 18: PIPELINE TO FILTER OUT UNDESIRABLE SEQUENCES. 22
SUPPLEMENTARY FIGURE 19: PIPELINE TO SELECT THE PUTATIVE NON-CODING MOLECULES. 23
SUPPLEMENTARY FIGURE 20: THE LNCRNA CLASSIFICATION. 23
SUPPLEMENTARY FIGURE 21: REPEAT LANDSCAPE. 24
SUPPLEMENTARY FIGURE 22: PCA PLOT OF CONTROL AND TREATMENT SAMPLES. 24
SUPPLEMENTARY FIGURE 23: AVERAGE OF TE DES. 25
SUPPLEMENTARY FIGURE 24: OVERVIEW OF METABOLOMICS. 26
SUPPLEMENTARY FIGURE 25: THE DEGREE-DEGREE CORRELATION ANALYSIS FOR CONTROL (C) AND TREATMENT (T) NETWORKS USING THE NORMALIZED DDC FUNCTION. 27
SUPPLEMENTARY FIGURE 26: 28
SUPPLEMENTARY FIGURE 27: CLUSTERING OF DEGS OF ETOH BUFFERING MODEL. 28
SUPPLEMENTARY FIGURE 28: SUBNETWORKS OF BMA64-1A AND S288C WITH GENES FROM DIAUXIC SHIFT PATHWAY, ETOH BUFFERING MODEL, AND THE LNCRNA TRANSCR_20548 OF BMA64-1A. 29
SUPPLEMENTARY FIGURE 29: TIME-COURSE EXPRESSION PROFILE OF GENES RELATED TO THE DNA REPAIR. 29

List of Tables

Supplementary Table 1: Strains description and results of EtOH tolerance. 30
Supplementary Table 2: Metrics of BMA64-1A genome considering all assembling. 30
Supplementary Table 3: Statistical analysis of cell viability and SDH assays comparing control *vs* treatment. 30
Supplementary Table 4: The number of genes significantly differentially expressed. 31
Supplementary Table 5: Summarization of GO terms of DEGs. 31
Supplementary Table 6: Significant KEGG pathways. 32
Supplementary Table 7: Number of lncRNAs per biotype and strain after filtering. 33
Supplementary Table 8: Number of significant differentially expressed lncRNAs. 33
Supplementary Table 9: Summary of lncRNA-protein interactions (LNCPI). 33
Supplementary Table 10: Topological features of all integrated networks. 33
Supplementary Table 11: Statistical differences of topological features of network's nodes comparing treatment vs control. 34
Supplementary Table 12: Pearson correlations between relative pH and K under different EtOH stress conditions. 34
Supplementary Table 13: Fold-change values from the ROS, SDH, and DNA damage assays. 34
Supplementary Table 14: Overview of DAPs. 35
Supplementary Table 15: List of DEGs in our data responsive to many stressors. 36
Supplementary Table 16:Ordinary differential equations (ODEs) used to simulate the dynamic network of BMA64-1A. 36
Supplementary Table 17: Parameters used in the ordinary differential (ODE) equations. 37
Supplementary Table 18: Target-proteins selected for the secondary structure comparison. 37
Supplementary Table 19: Primers used in this paper. 39
Supplementary Table 20: Overview of *de novo* re-assembling of S288C's lncRNAs by using CAP3. 39
Supplementary Table 21: Number of TEs here annotated. 40

Supplementary Figures

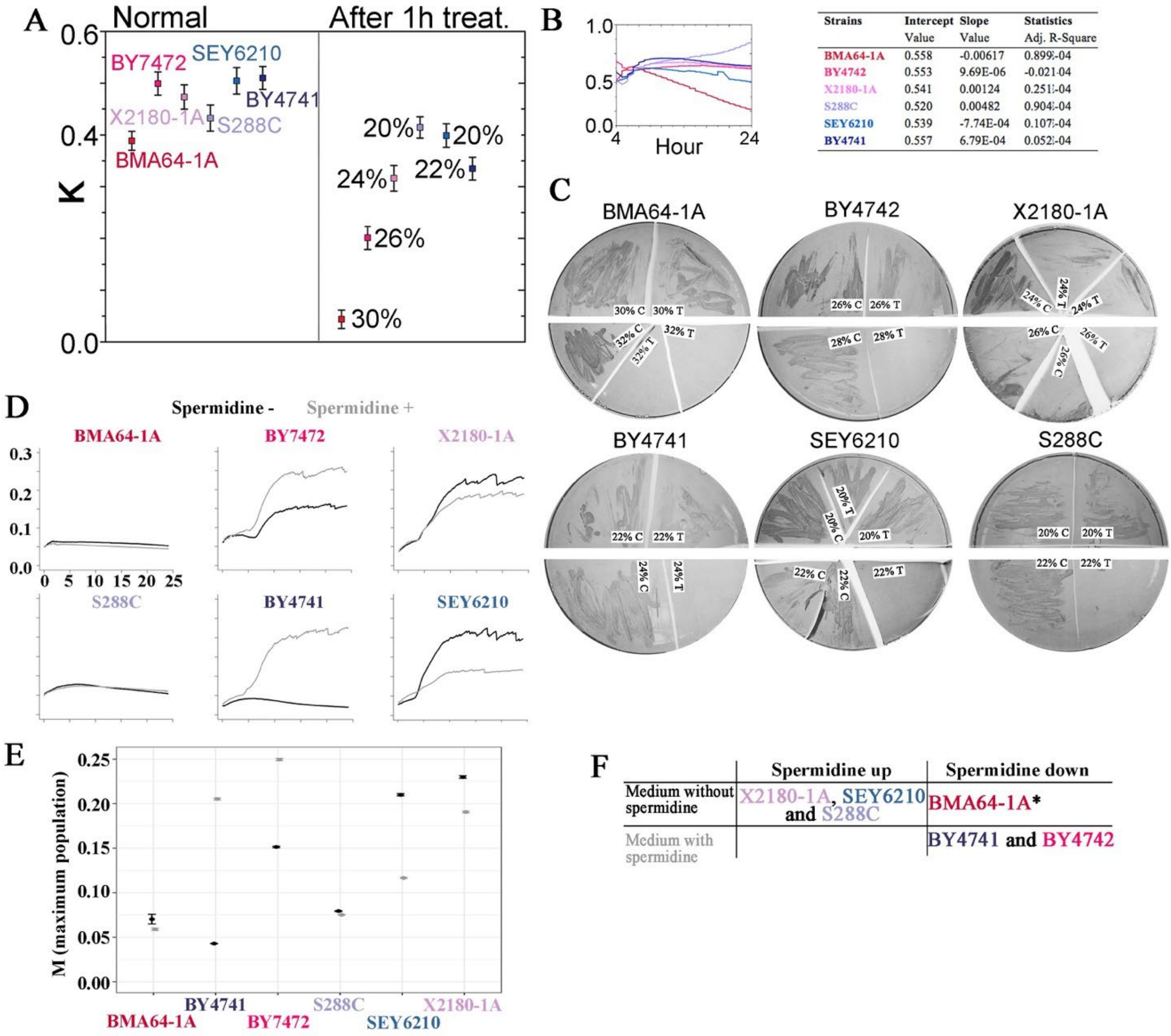

Supplementary Figure 1: The growth curve analysis for the 6 selected strains.
A: the left plot reports the population growth in the normal growth condition, and the right plot presents the normal conditions after the treatment under the highest EtOH level for 1h (stress relief). The colors follow the definitions in B; B: growth curve under the highest EtOH level. The values in time 0 intercept Y-axis at ~0.5, showing similar initial population for all strains. Interpolations are reported in the right-side table. Slope ~1 means growth; C: plates with the maximum tolerated EtOH stress per strain. C is control and T is treatment; D: growth curves in spermidine rich medium after the treatment under the highest EtOH level; E: plot of maximum population from the experiments described in “D”. The colors follow the description in “D”; F: strains lie in a confusion matrix according to their best growth-behavior of experiment described in “D”. Then, we could compare to what was expected based on metabolome data (depicted in the first line of this matrix) *: label the only strain that did not fit the expected growth-behavior under spermidine rich medium.

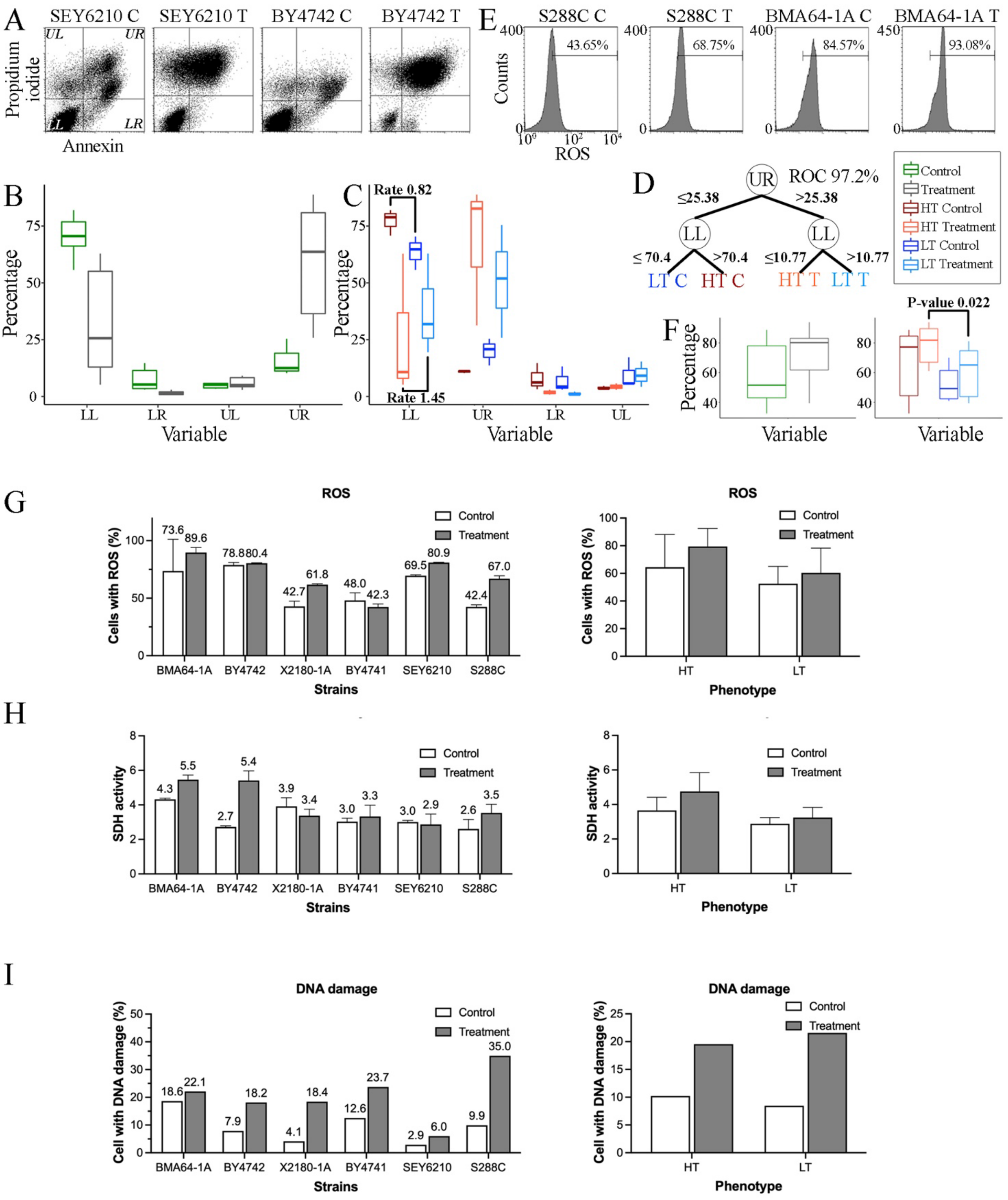

Supplementary Figure 2: Cell viability, SDH, ROS and DNA damage quantification.
A: Representative flow cytometry plots to assess the cell viability using Annexin V and propidium iodide costaining. The quadrants of the plot indicate viable (live) cells (LL), cells undergoing initial apoptosis (LR), and cells with advanced death (UL and UR). The set with LR, UL and UR includes all dead cells; **B:** cellular viability of all strains; **C:** comparing cellular viability within HTs and LTs. The "rates" are the averages of LT divided by HT (rate >1 indicates more LTs, whereas rate <1 indicates the other way around); **D:** decision tree to assess the relationship between LL and UR variables. The edges' numbers are the percentage of cells under the condition branched to the adjacent node; **E:** examples of flow cytometry to assess the number of cells accumulating ROS; **G:** ROS analysis of all strains (left boxplot), and within HTs and LTs (right boxplot) using CellROX Green; this kit allows to determine the percentage of cells with ROS primarily in the mitochondria and nucleus. P-values was calculated by T-test; **H:** bar graph depicting the quantification of absorbance of chemical

staining with Nitro Blue Tetrazolium to assess the SDH activity; **I:** DNA damage analysis of all strains (left boxplot), and within HTs and LTs (right boxplot) using fluorescent anti-H2AX antibody (biomarker for nuclear DNA damage).

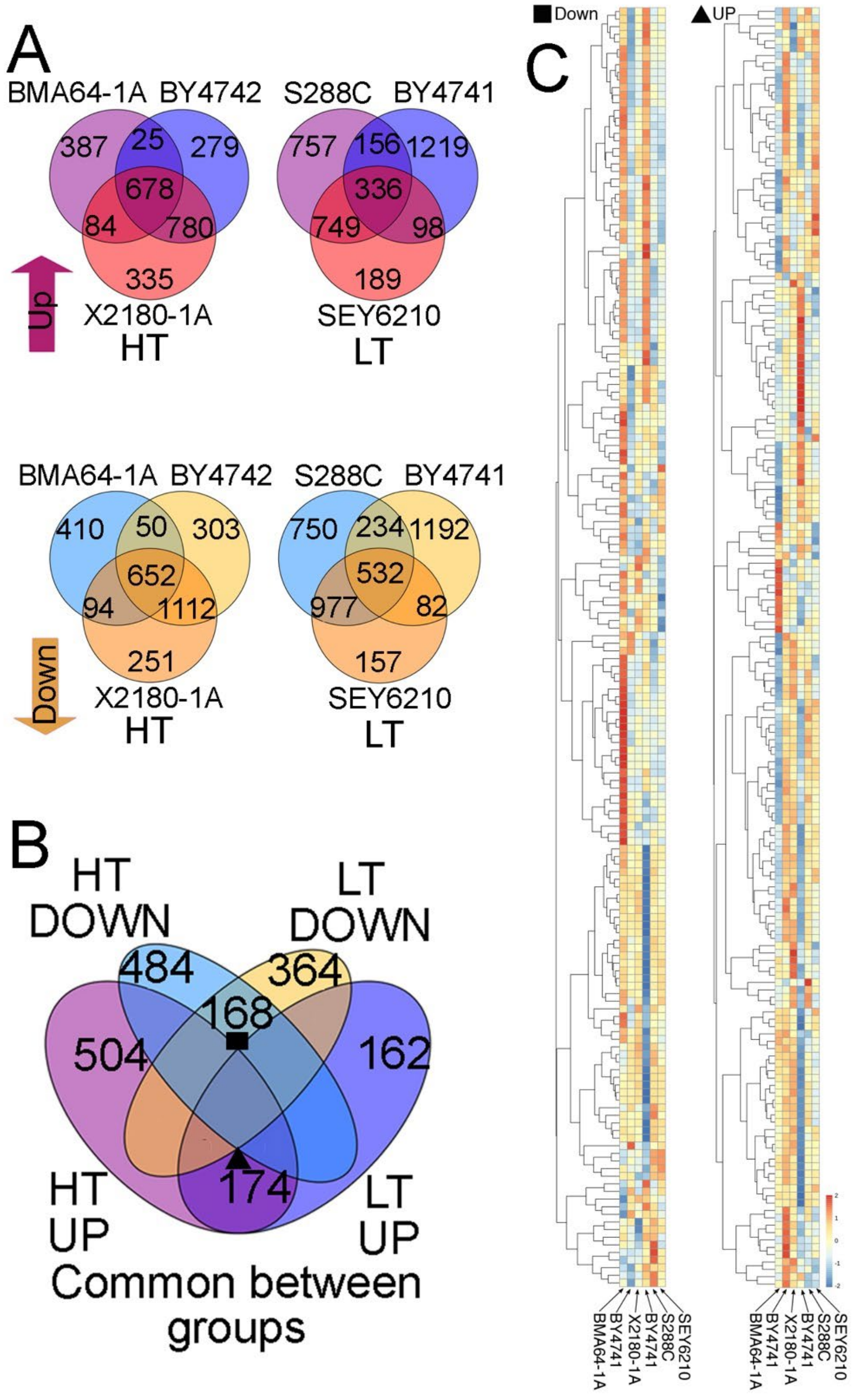

Supplementary Figure 3: Set analysis of differentially expressed coding genes.
A-B: DEGs among strains and phenotypes. Panel B depicts sets between the up- and downregulated DEGs from the core of the Venn diagrams in A; **C:** The triangle and square in the heatmap data indicates the same sets from Panel B; **Up:** up-regulated; **Down:** down-regulated.

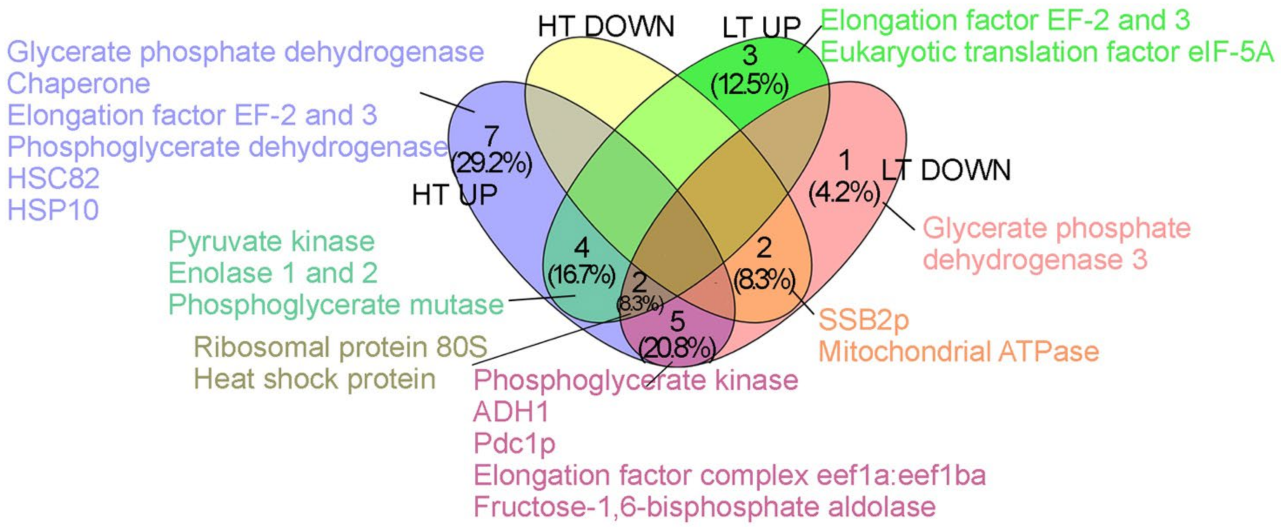

Supplementary Figure 4: Differentially abundant proteins (DAPs) between phenotypes.

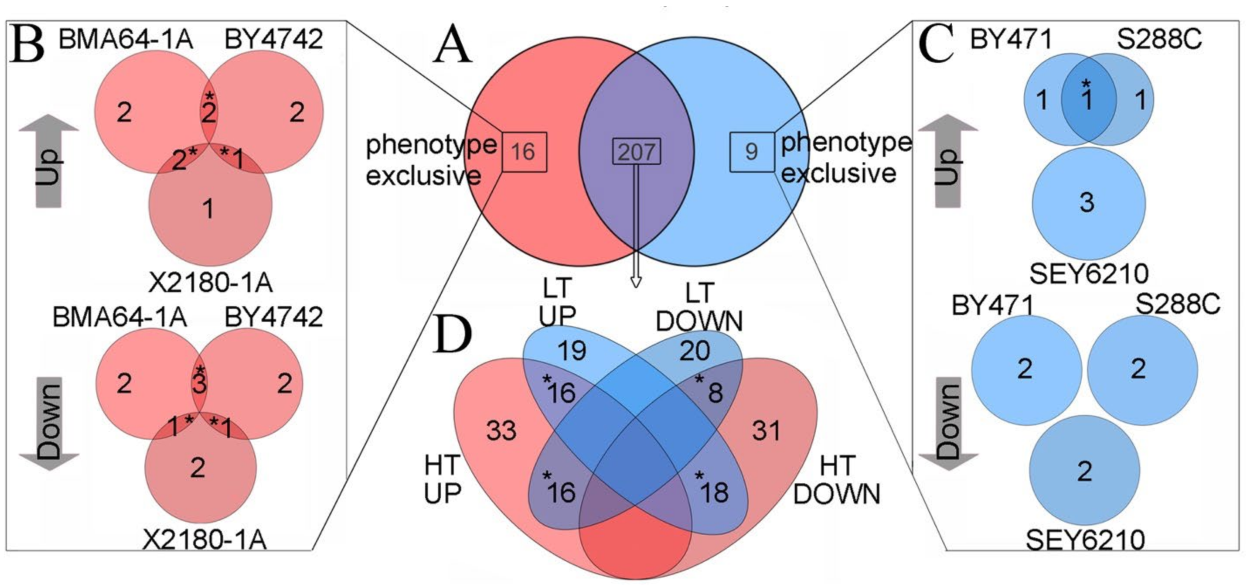

Supplementary Figure 5: Differentially abundant metabolites (DAMs) comparing strains and phenotypes.

A and D: DAMs with phenotype-specific EtOH stress-response. Metabolites with significantly increased abundance in at least two strains of a phenotype under stress were considered to have phenotype-specific induced synthesis. Conversely, metabolites with reduced abundance were considered metabolites with suppressed synthesis; B and C: the number of DAMs modified in each strain (B and C). * indicates DAMs mapped onto KEGG pathways; "Up" and "Down" are induced and repressed metabolites, respectively.

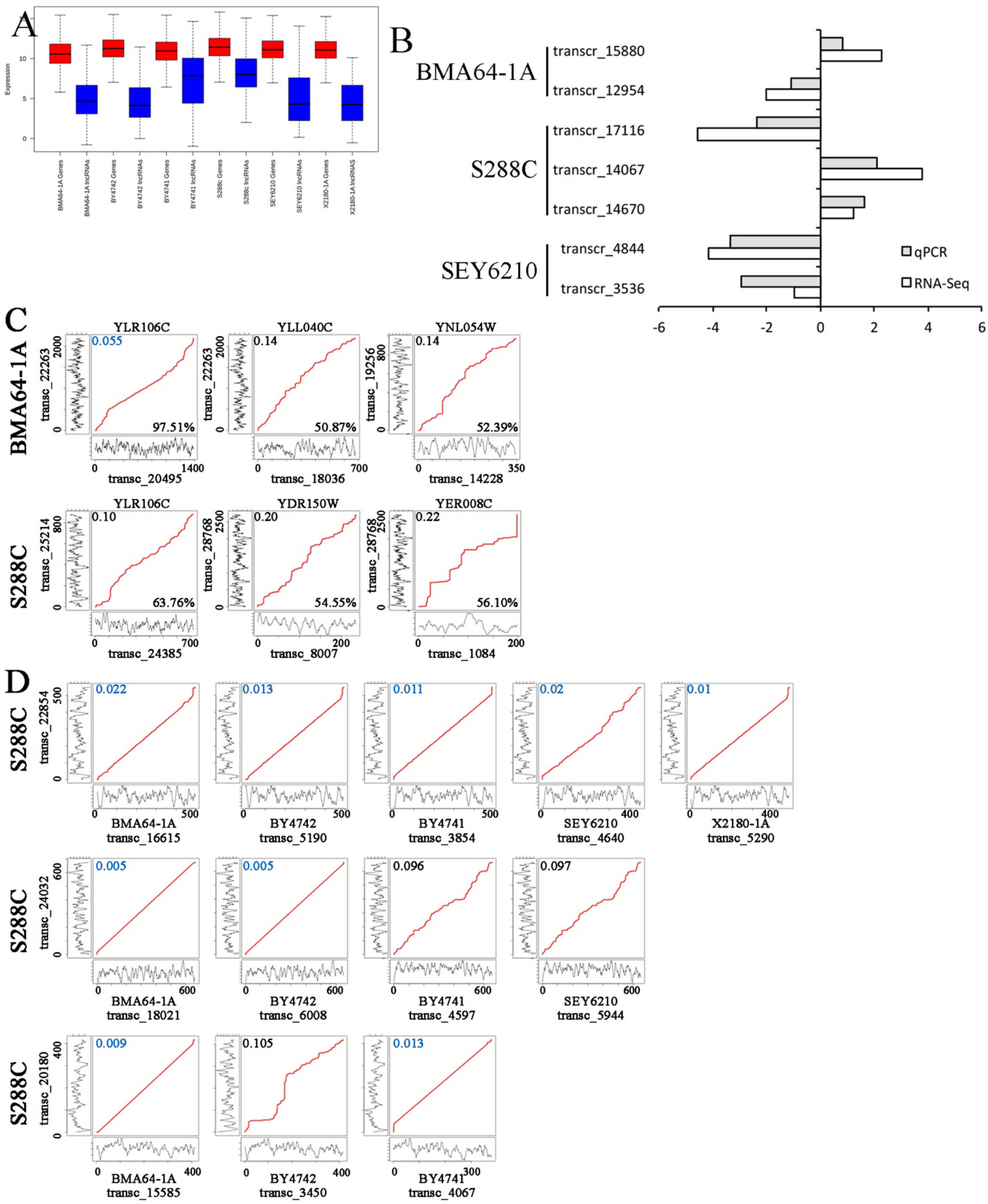

Supplementary Figure 6: Overview of lncRNA expression and Secondary structure comparisons of lncRNAs.

A: comparison between the lncRNAs and coding gene expression; **B:** qPCR. X axis is the L2FC. **C:** intra-strain comparisons of lncRNAs which target the same proteins (described on the top of boxes). The percentages are the sequence similarity between lncRNAs; **D:** inter-strain comparisons between lncRNA of S288C against orthologues. The structural distance score (SDS) is at the left top, which blue (<0.095) indicates similar secondary structures between lncRNAs (p-value <0.05). The axes are the structural profile provided by the CROSS Global Score.

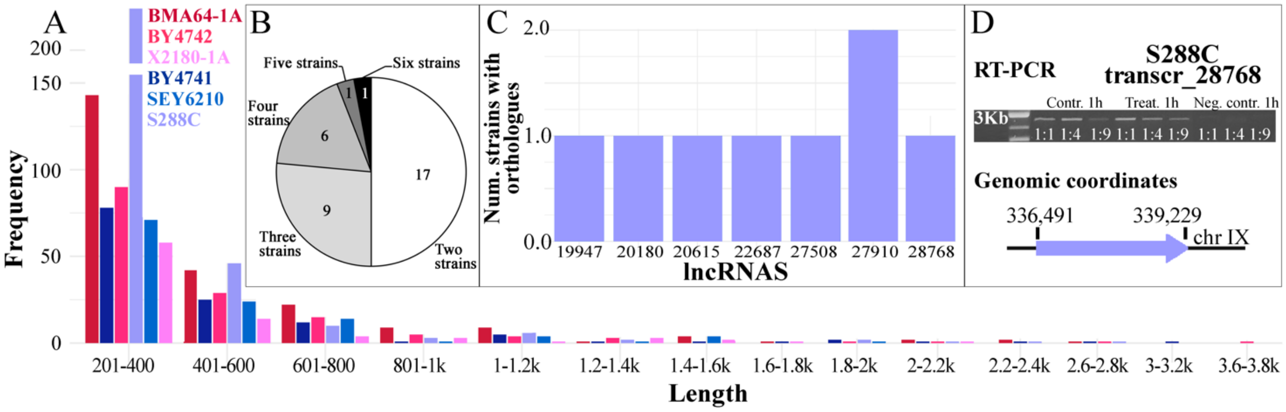

Supplementary Figure 7: Length of lncRNAs and orthologues matching.
A: the distribution of lncRNA lengths; **B and C:** the number of S288C's lncRNAs which present orthologues in other strains with a similarity level ≥80% ("B") and ≥90% ("C"), respectively. The pie chart numbers refer to the number of S288C's lncRNAs with orthologues; **D:** RT-PCR to check the expression of the longest lncRNA found and its genomic coordinates. Modified from.

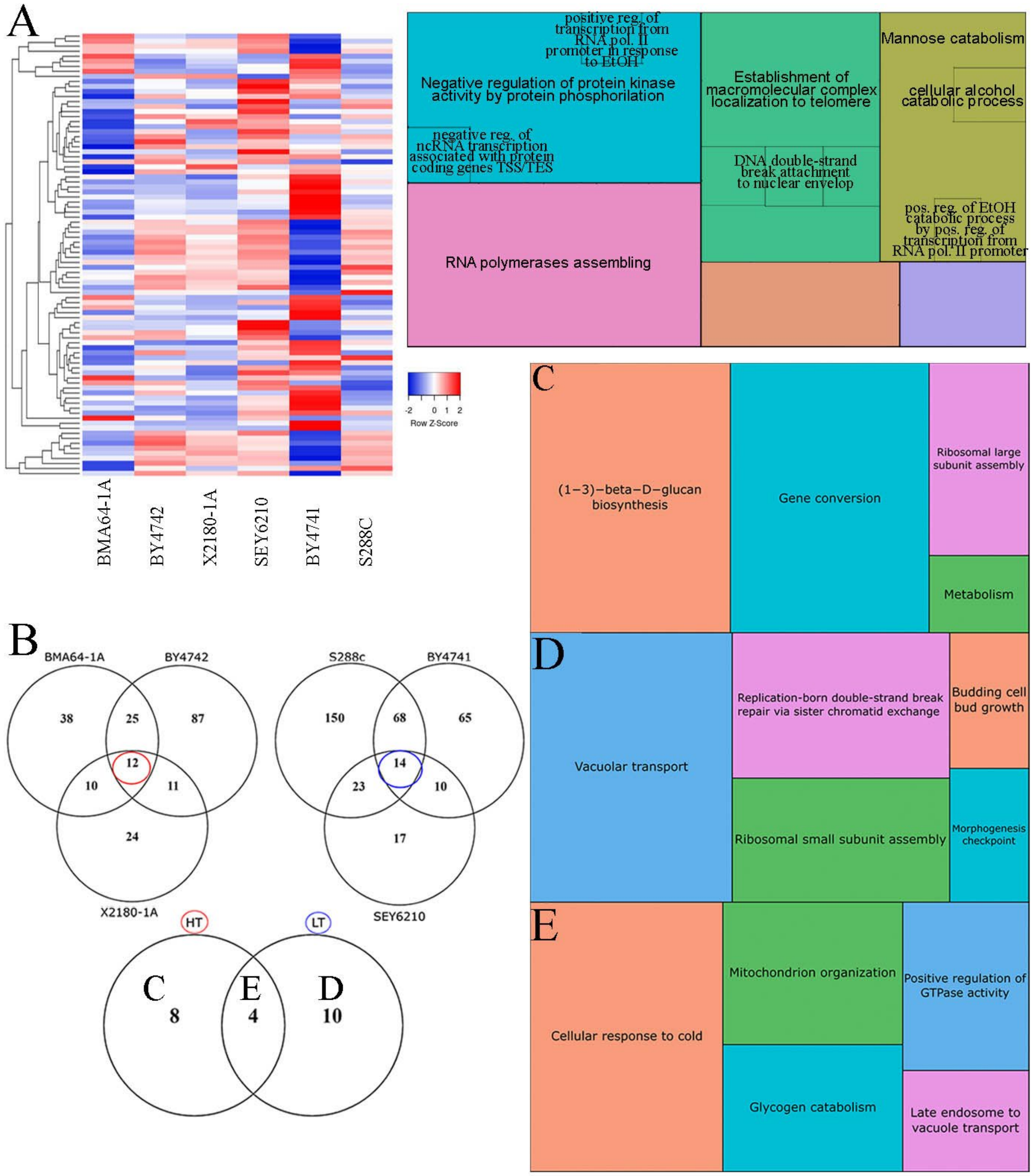

Supplementary Figure 8: GO enrichment analysis of lncRNAs using g:Convert.

A: terms from all target-proteins without consider neither strains nor DE, and without exclude redundant proteins. Heatmap is related to 88 genes working on “transcription by RNA polymerase II” (from “RNA polymerase assembling” in the right box); **B-E:** analysis considering DE target-proteins; **B:** intersections among target-proteins; **C-E:** terms of target-proteins from the Venn diagram at the bottom of figure.

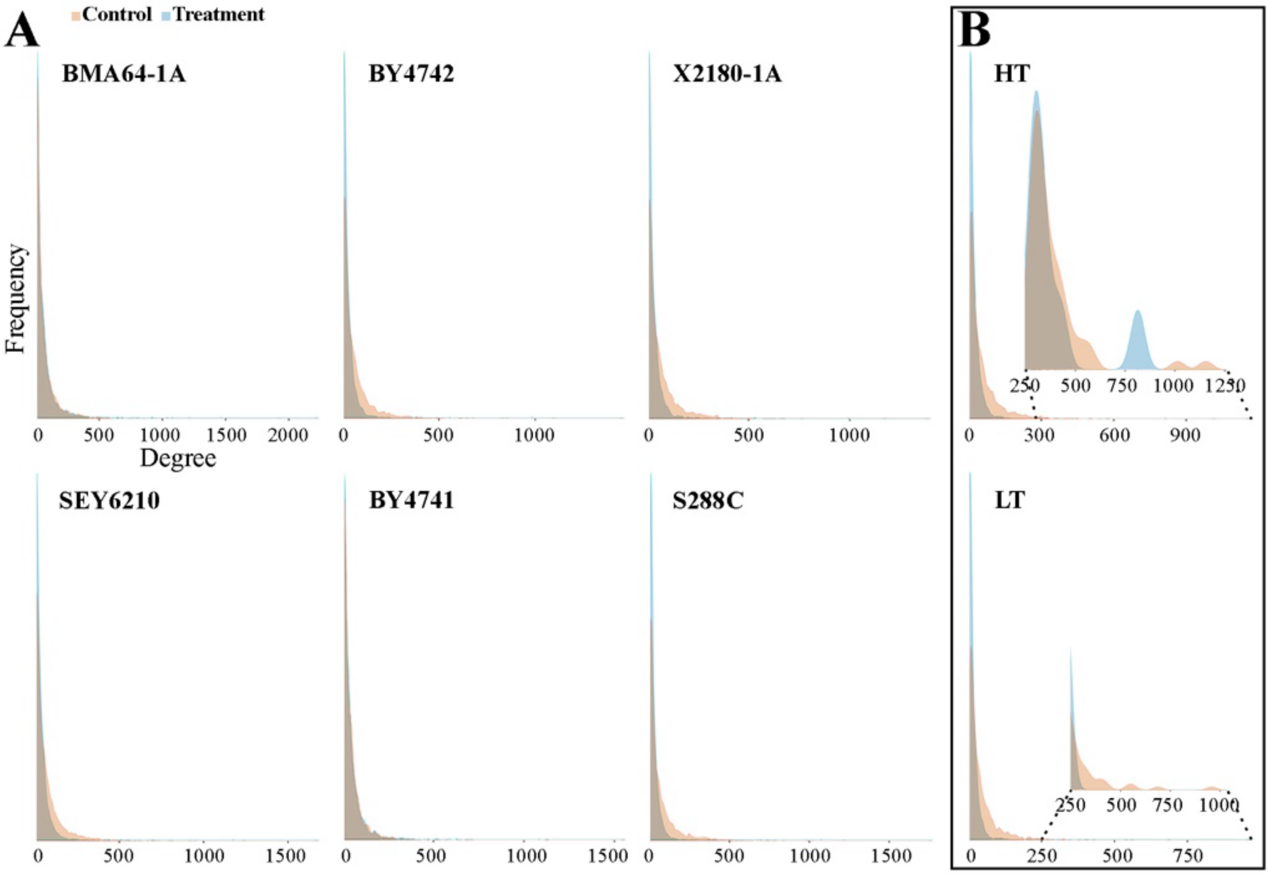

Supplementary Figure 9: The network degree distribution.
A: histogram per strain; **B:** histogram per phenotype highlighting the tail distribution.

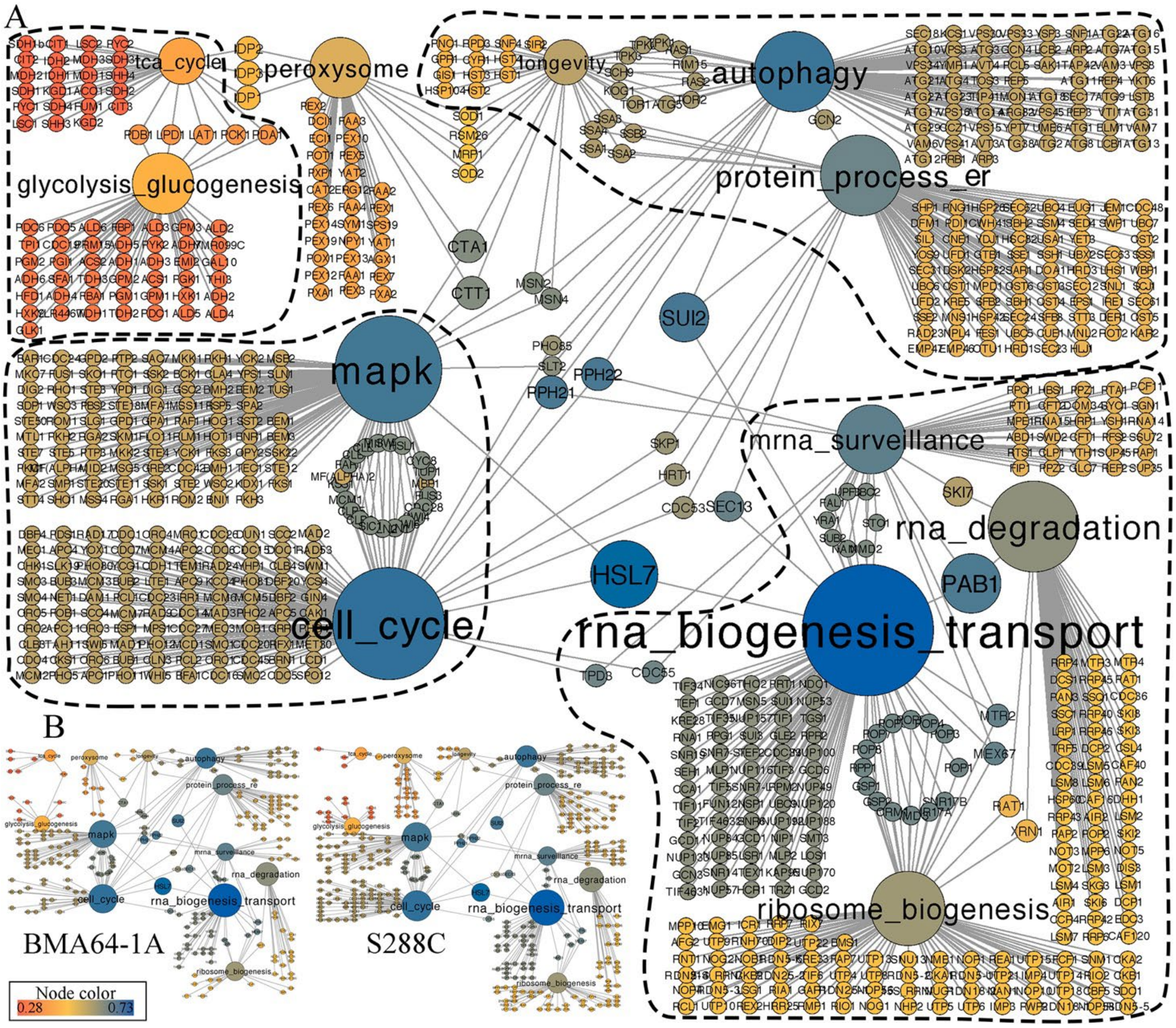

Supplementary Figure 10: Communities of Pathway Integrated Network (PINET) and the rewiring after EtOH stress integrating the time-course.

A: raw PINET. The dashed lines circumvent the main communities; **B:** HT and LT networks created after the clustering using the time-course data. The node size and color are based on betweenness centrality and radiality, respectively (larger and blue nodes are the most important ones). The https://figshare.com/s/0ef043d4cd1d8b1f0bb1 presents these networks in the Cytoscape file format to allow an interactive data visualization.

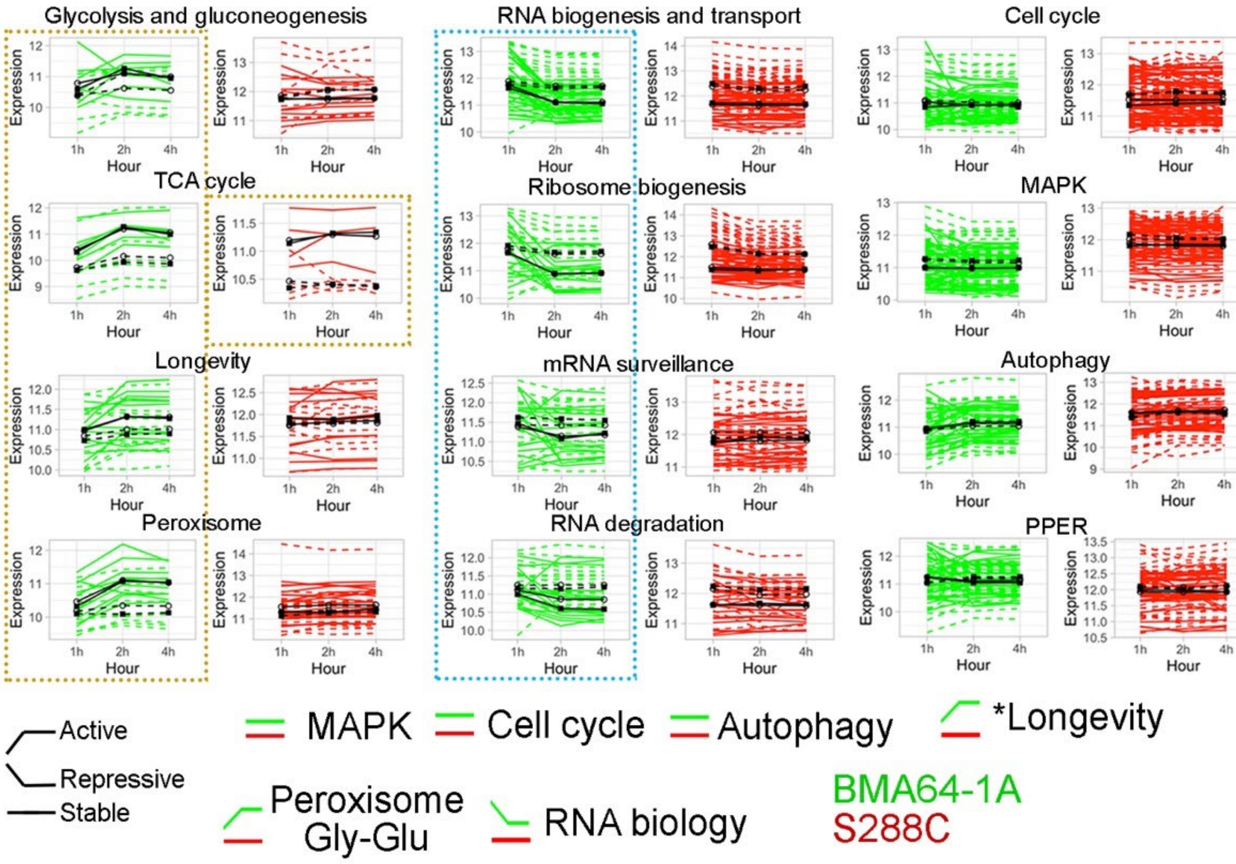

Supplementary Figure 11: Expression profile of genes linked to pathways (*e.g.,* "cell cycle").
The expression profiles of cells under EtOH stress were determined for each pathway by averaging the expression of genes in each time-point, independently. Dashed and full lines indicate the control and treatment conditions, respectively. Black lines with squares and circles are the median and average, respectively.

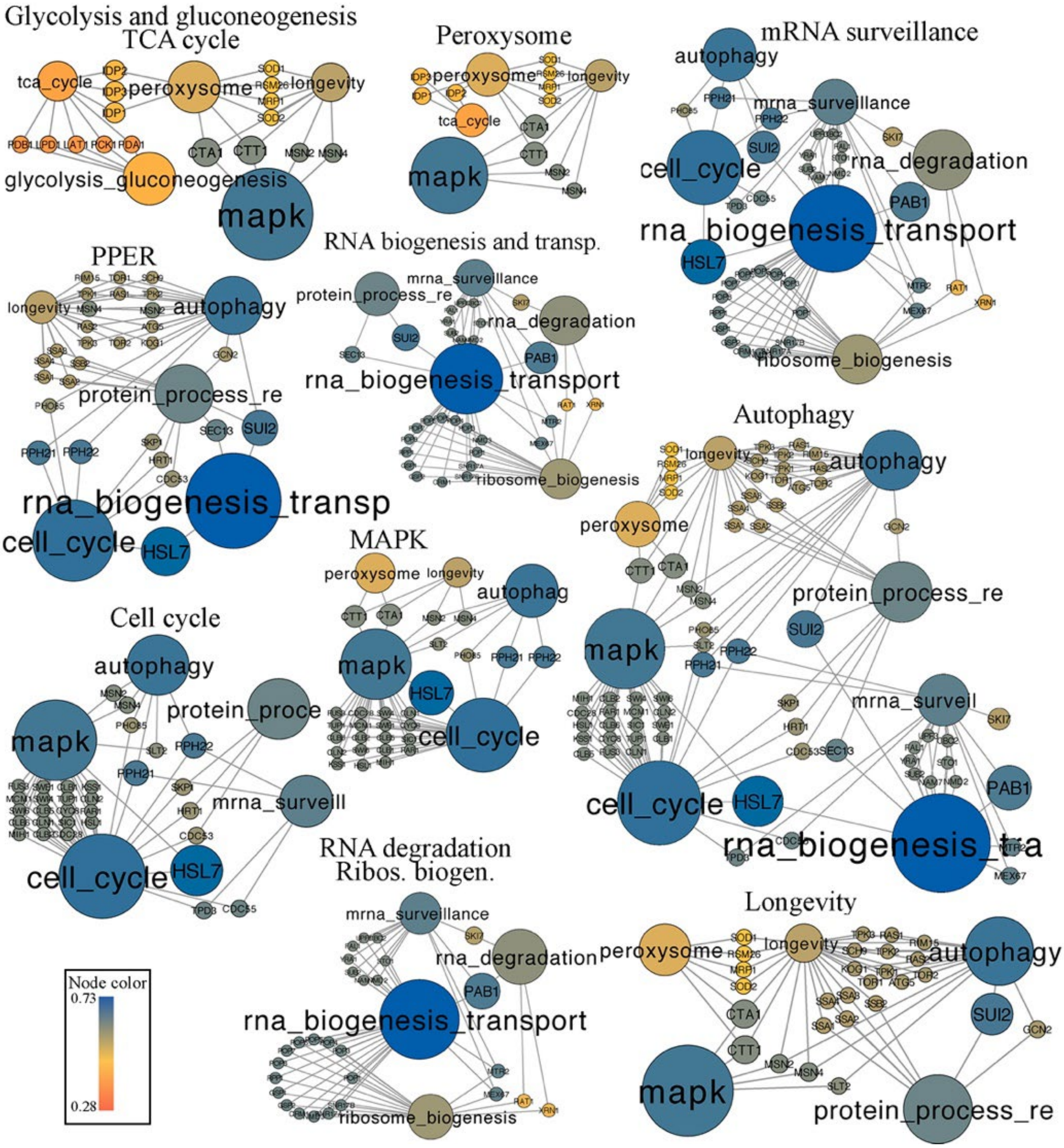

Supplementary Figure 12: Network propagation (systems signaling analysis) from each pathway based on the pathway integrated network (PINET).
The https://figshare.com/s/0ef043d4cd1d8b1f0bb1 presents these networks in the Cytoscape file format to allow an interactive data visualization of systems signaling analyzes from each pathway.

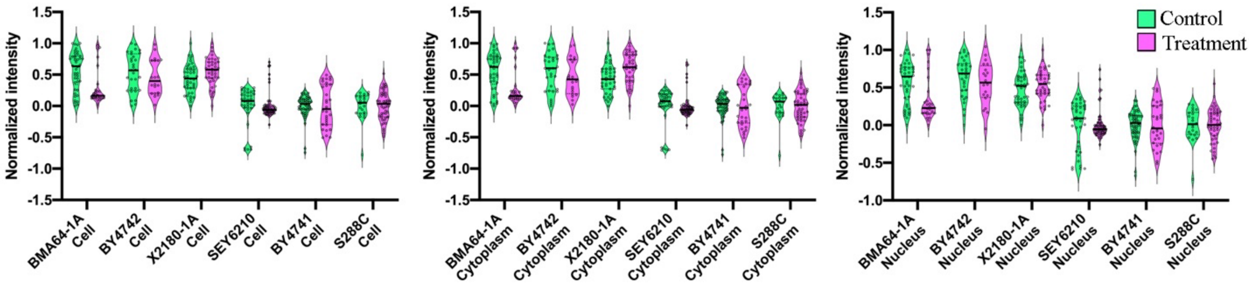

Supplementary Figure 13: RNA yield quantification by acridine orange assay.

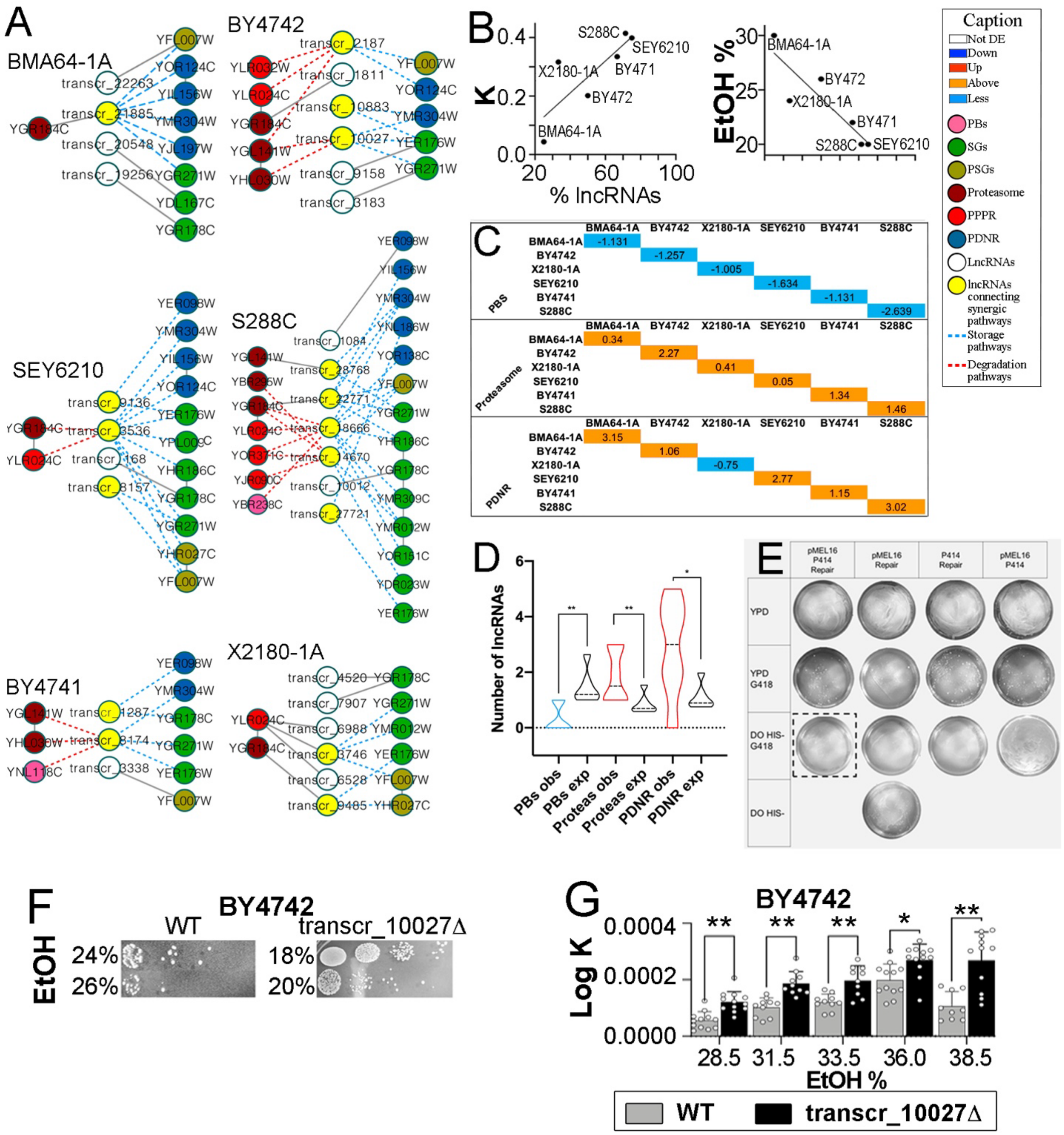

Supplementary Figure 14: Analysis of genes and lncRNAs related to membraneless structures or degradation/storage pathways responsive to the EtOH stress.

A: Initial analysis ranked 70 lncRNAs linked to 191 proteins of storage and degradation pathways. However, the systems signaling analysis using the diffusion algorithm (Carlin et al. 2017) from each EtOH stress-responsive lncRNA ranked these 30 most significant lncRNA-protein interactions for each strain related to degradation and storage systems (see Section 2.4 of the main text). The genes that lie on left and right sides of lncRNAs are related to degradation, and storage pathways, respectively; **B:** regression comparing the percentages of lncRNAs connecting synergistic pathways *vs*. the growth rate (K) after EtOH stress relief (R^2=0.66, p-value=0.04) or the highest supported EtOH level analyzed (R^2=0.79, p-value=0.01); **C:** structures/pathways with significant higher or lower number (T-test) number of lncRNAs compared to expected. The numbers are the rate between observed *vs* expected values; **D:** the number of lncRNAs observed and expected reported in "B". * is p-value <0.05 and ** is p-values <0.01; **E:** lethality tests of SEY6210 transcr_3536Δ mutant. The first line reports the combination of vectors and repair DNA tested, while the first columns are the mediums. The YPD plates evidenced that all experiments allowed cell surveillance in a rich medium. The YPD + G418 with P414 showed that this vector is expressed. The same can be concluded for the pMEL16 plated, or not, in drop-out (DO) His- medium. Based on the expected profiles observed, and the one harboring the two vectors + repair DNA on DO His- + G418 (the box dashed figure) plate, we conclude

that both vectors are properly working (expressing Cas9 and His marker) and that the presence of repair DNA is responsible to induce the lethality by conducting the deletion properly; **F:** EtOH tolerance test of BY4742 WT and BY4742 transcr_10027Δ mutant; **G:** Population rebound of BY4742 WT and the BY4742 transcr_10027Δ mutant. * and ** represent p-values <0.05 and <0.01, respectively.

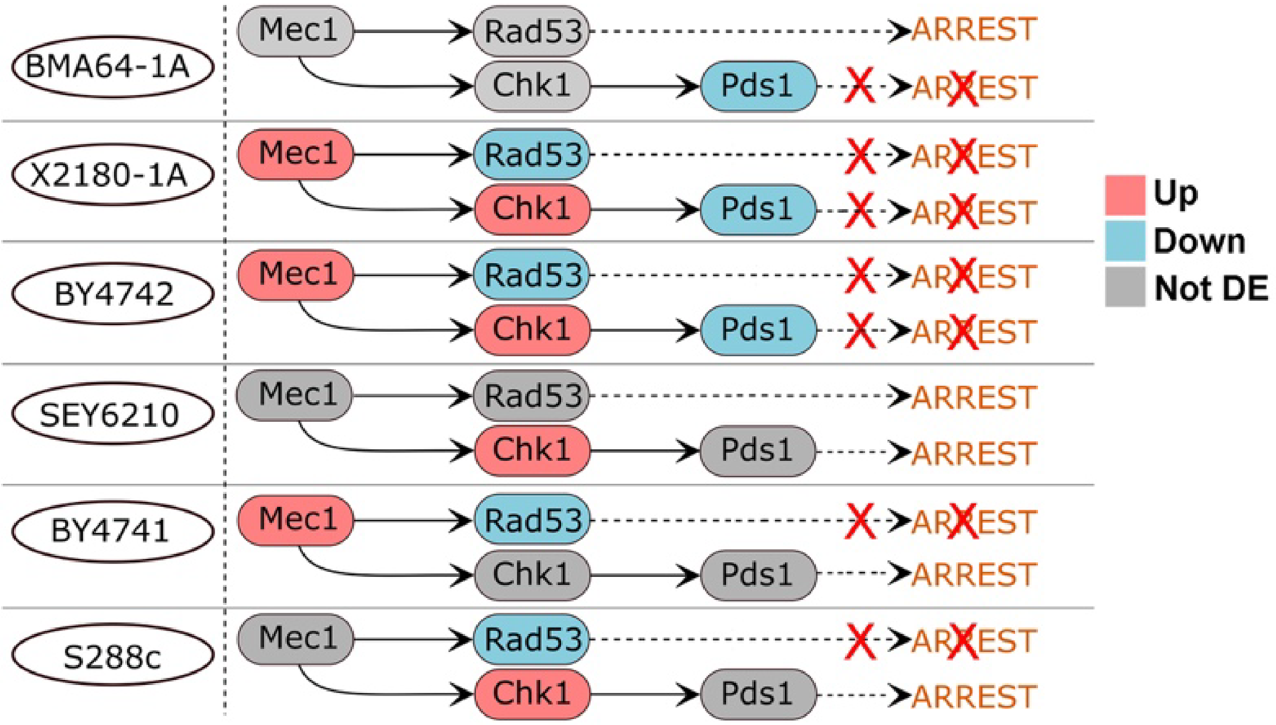

Supplementary Figure 15: Model of RAD53 and PDS1 mechanisms of DNA checkpoint regulation.
***:** putative cell cycle arrest of DNA repair. See the expression of genes in the **Supplementary Data 1**.

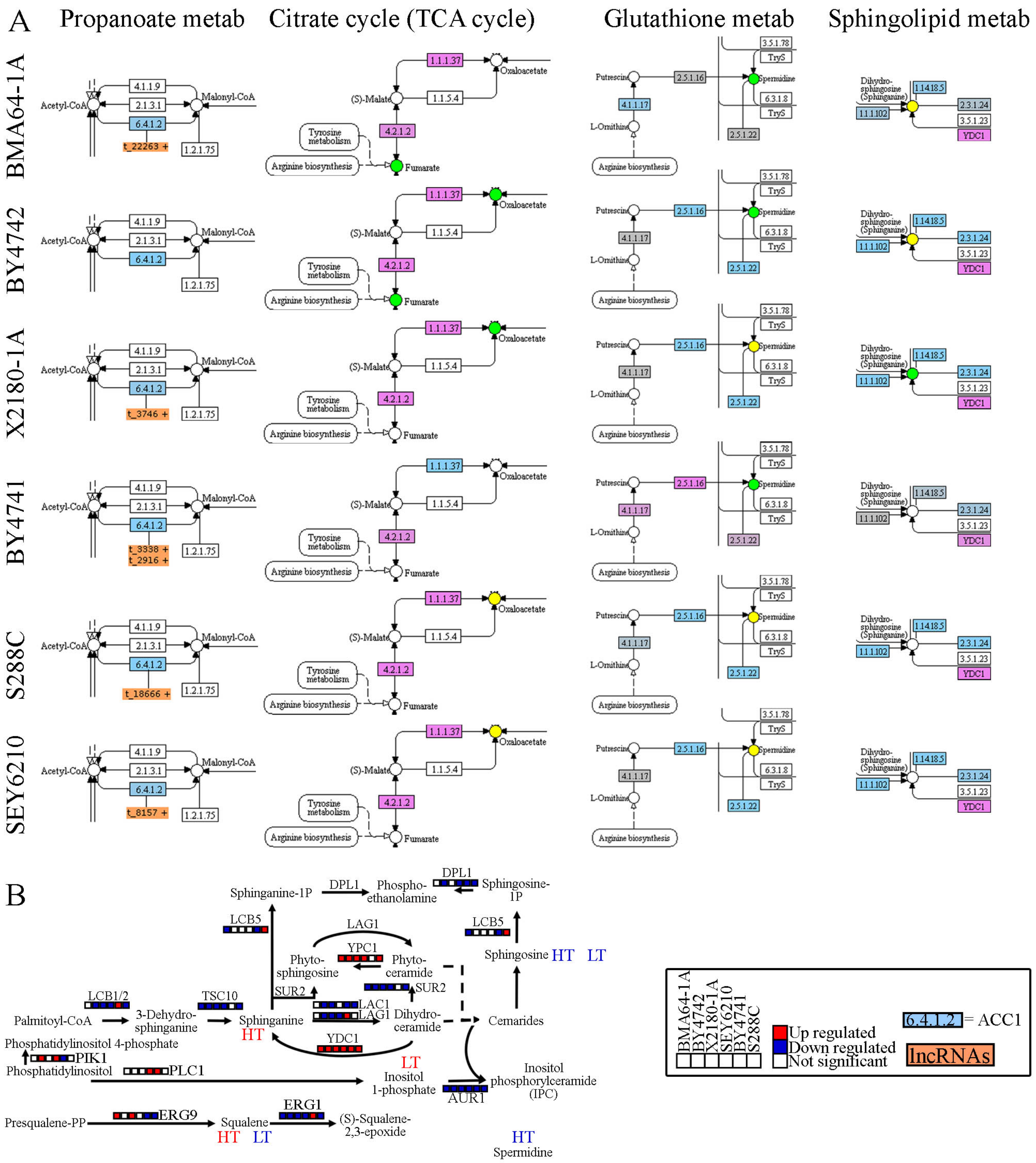

Supplementary Figure 16: Some KEGG metabolic pathways analyzed.
A: metabolic pathways with the DAMs also harboring lncRNAs; **B:** part of sphingosine and steroid metabolic pathways focus on sphinganine, squalene, and sphingosine metabolites. The caption is in the box. The sphingosine is the metabolite number YMBDB00190 in the YMDB database.

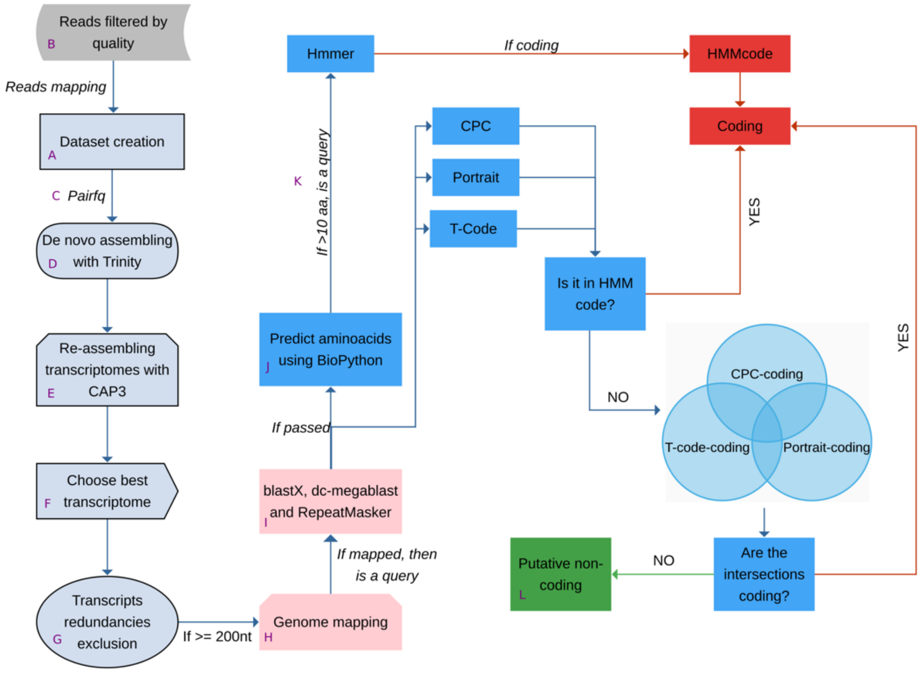

Supplementary Figure 17: Pipeline to assembly the lncRNAs.

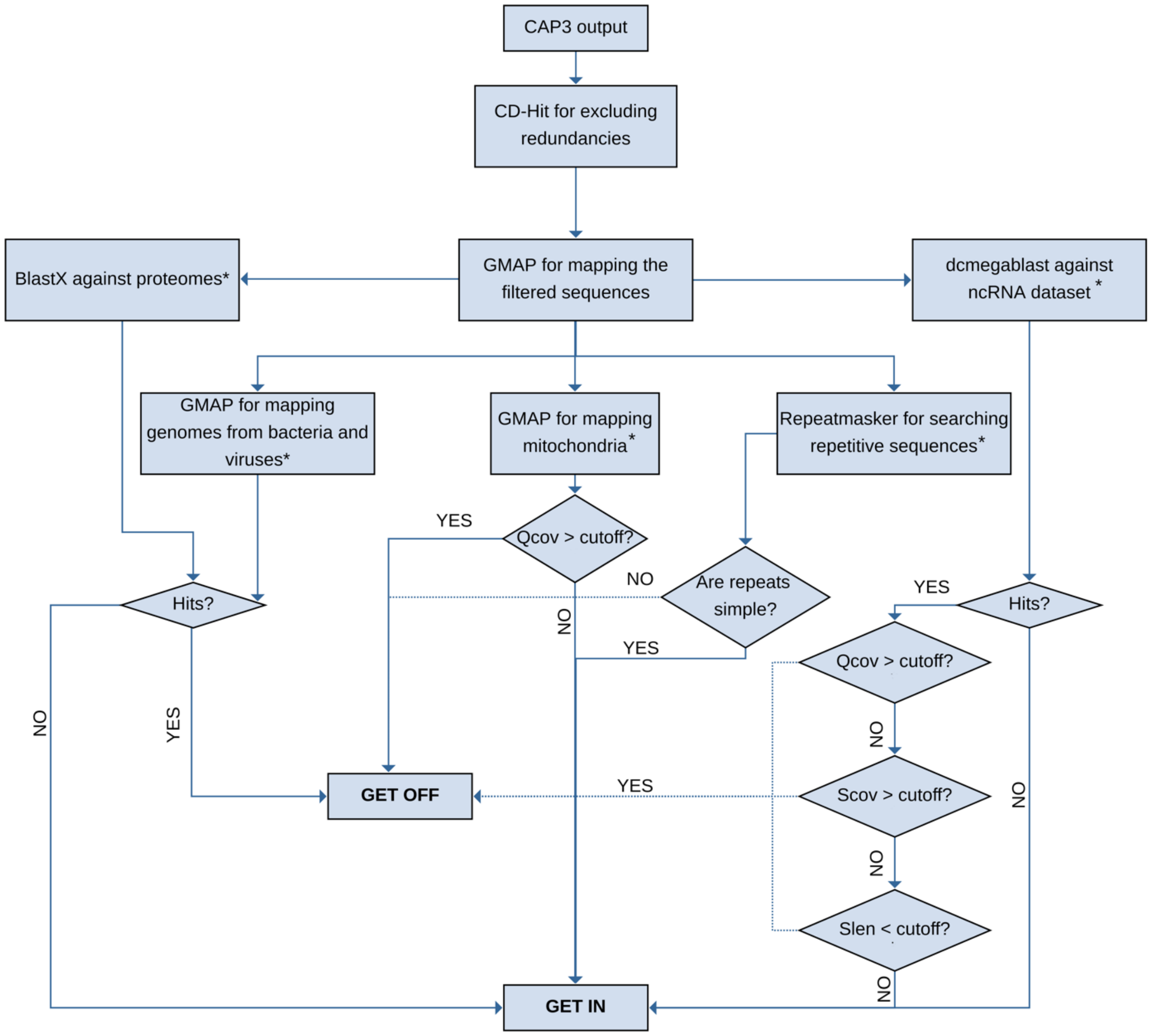

Supplementary Figure 18: Pipeline to filter out undesirable sequences.
***:** data distribution was evaluated to set-up the cutoffs (**Supplementary Data 13**).

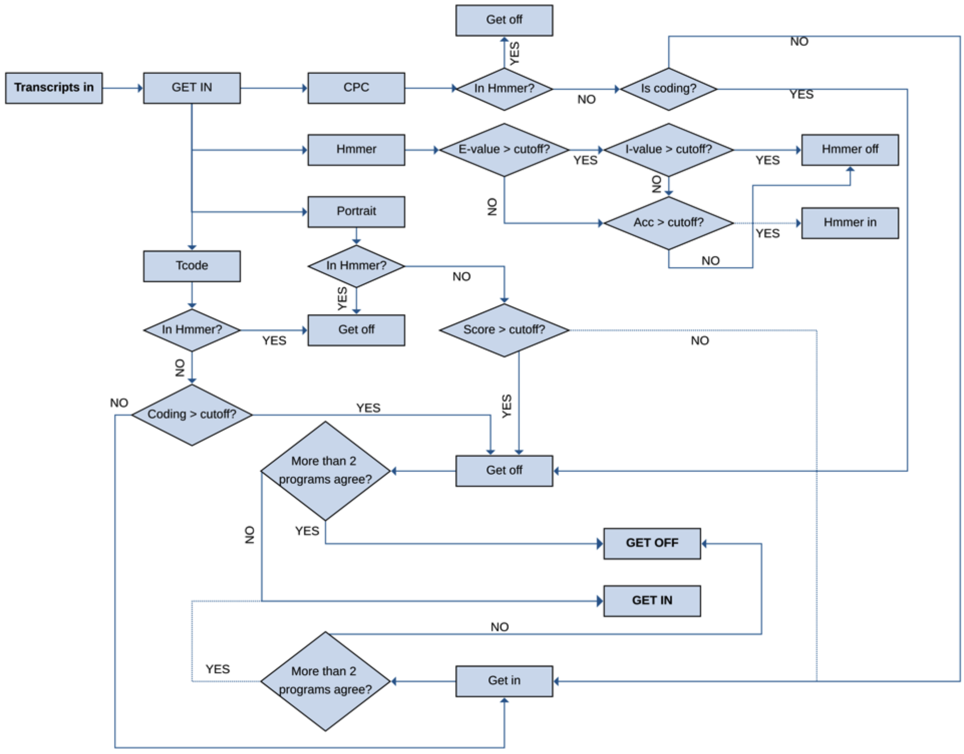

Supplementary Figure 19: Pipeline to select the putative non-coding molecules.
Transcripts evaluated as “Hmmer in” were automatically considered coding, whereas “Hmmer off” include transcripts that where compared to the output from other programs. **Get in:** sequences considered lncRNAs; **Get off:** coding sequences. Cutoffs are presented at **Supplementary Data 13**.

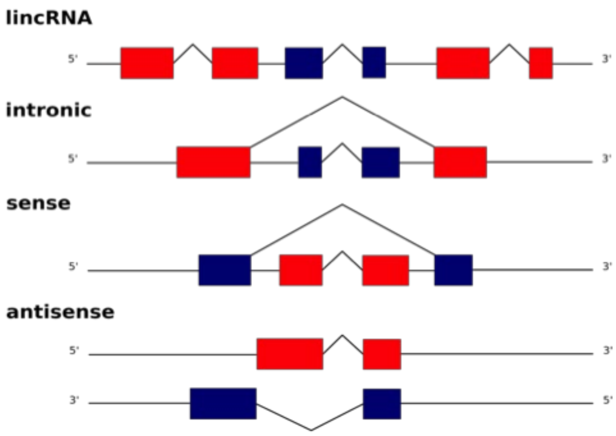

Supplementary Figure 20: The lncRNA classification.
Red box: exon of protein-coding genes; **Blue box:** lncRNA.

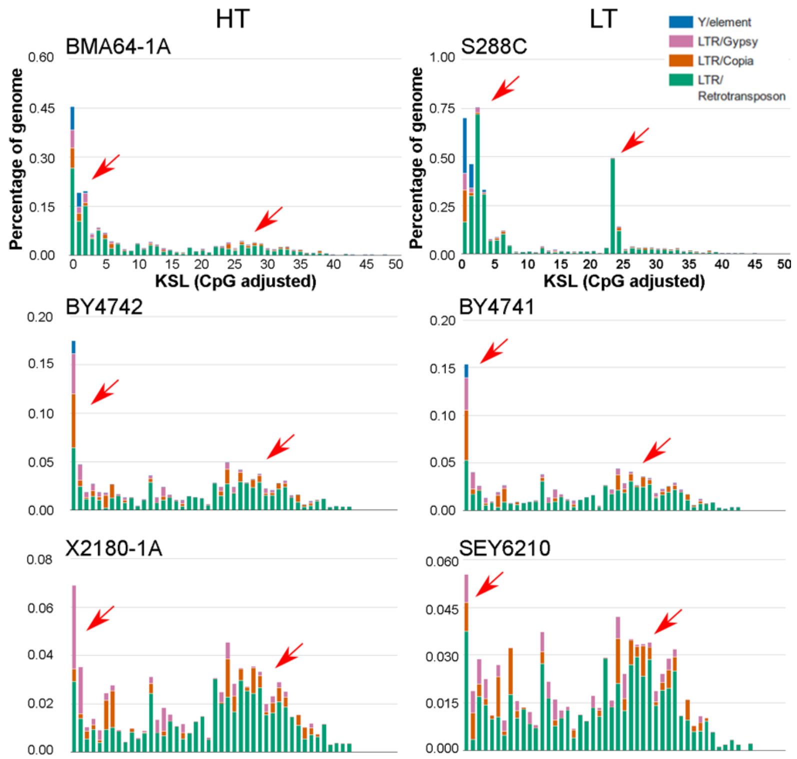

Supplementary Figure 21: Repeat landscape.
Red arrow: indicates peaks of insertion waves, being the left ones more recent (higher similarity to the consensus) and the right ones older; **KSL:** Kimura substitution level.

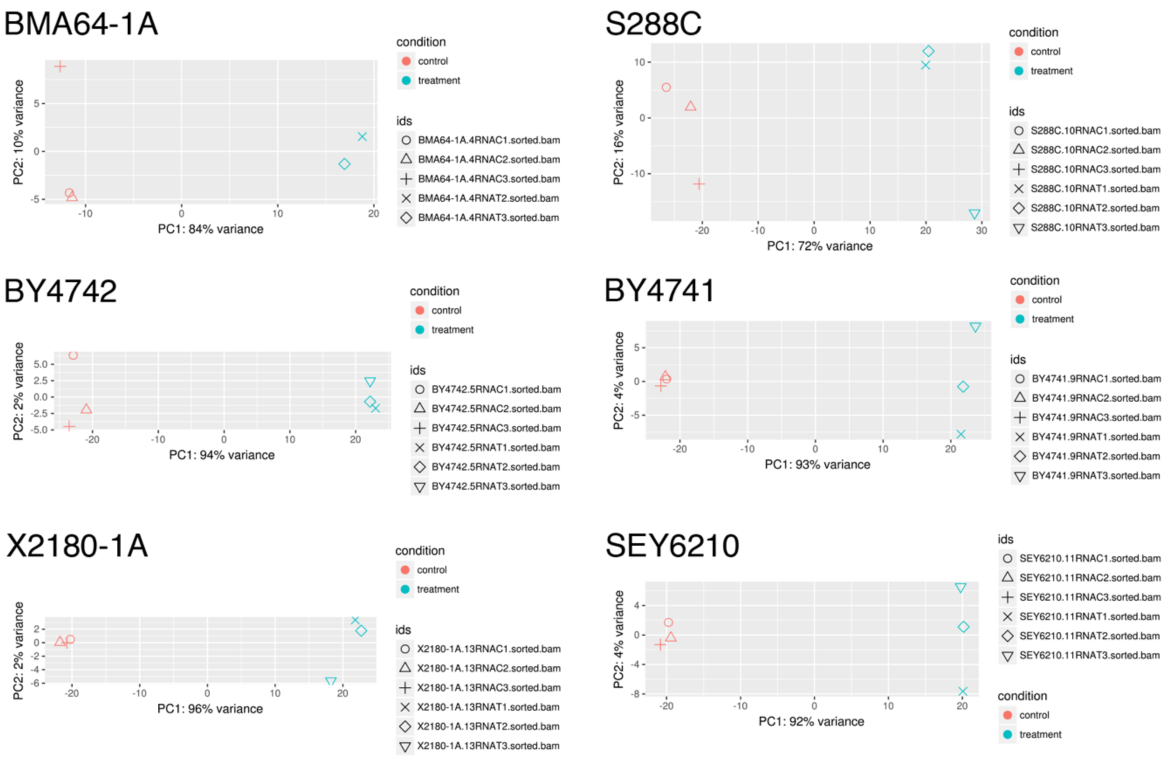

Supplementary Figure 22: PCA plot of control and treatment samples.

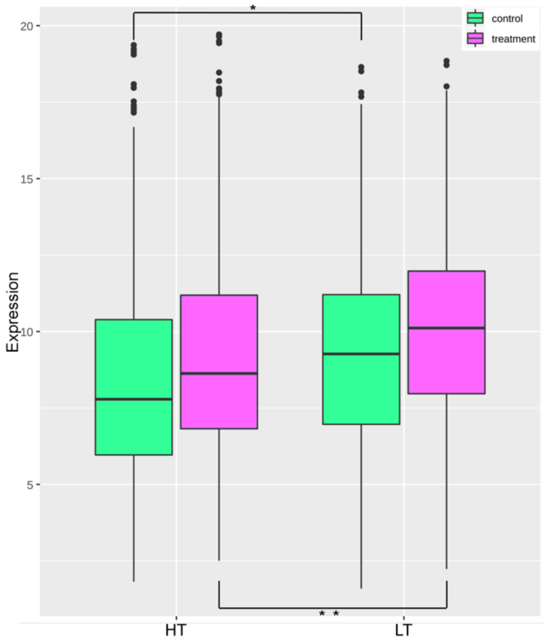

Supplementary Figure 23: Average of TE DEs.
:** p-value <0.05; *:** p-value <0.01.

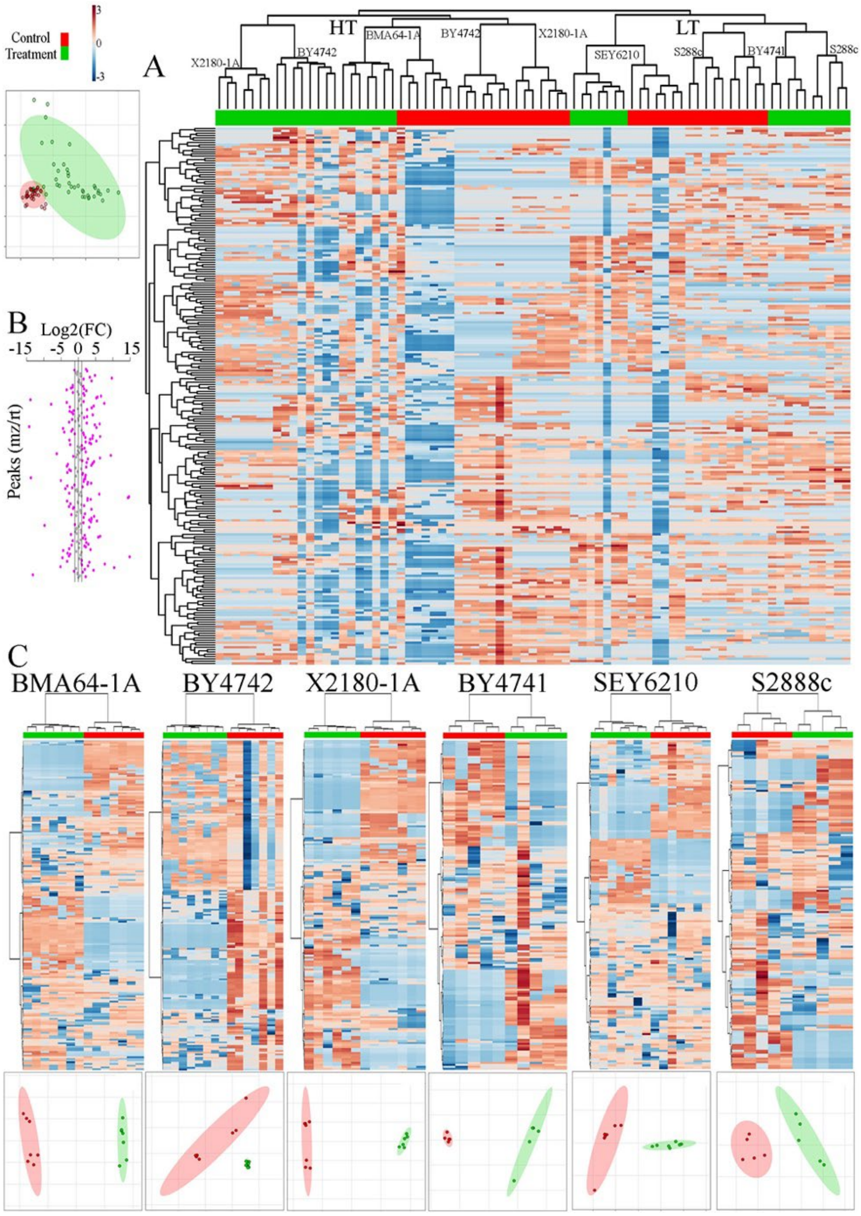

Supplementary Figure 24: Overview of metabolomics.
A: heatmap, and PLS-DA comparing all samples; **B:** histogram of DAM considering all samples; **C:** heatmaps and PCAs comparing samples within each strain. Heatmaps were performed using Pearson correlation. The principal components 1 and 2 are presented in PCA and PLS-DA.

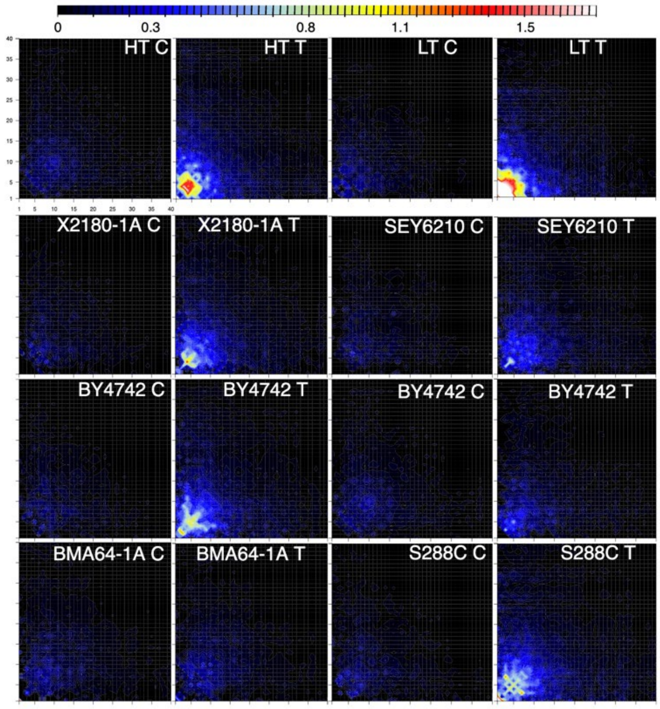

Supplementary Figure 25: The degree-degree correlation analysis for control (C) and treatment (T) networks using the normalized DDC function.

Scale shows the likelihood of two genes with degrees *k* and *k'* interact. Darker colors means reduced interaction while white colors means propensity for gene interaction.

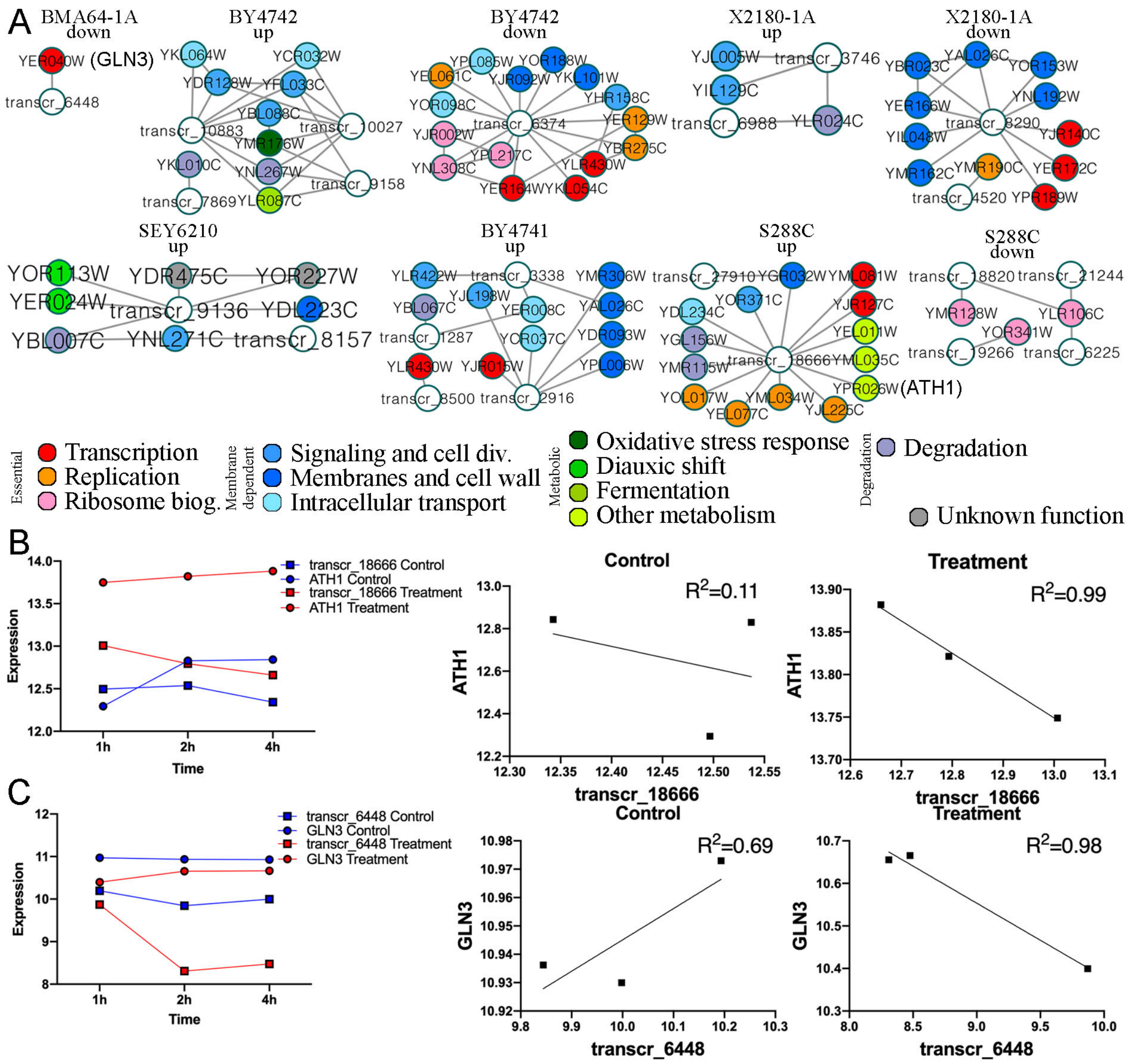

Supplementary Figure 26:
Analysis of subsystems from LNCPI selected by lncRNA-propagation analysis. A: selected subsystems. The color nodes are related to biological functions depicted under the graphs; **B-C:** expression profile and regression analysis of two lncRNAs and their target-proteins. These genes are within parenthesis on “A”.

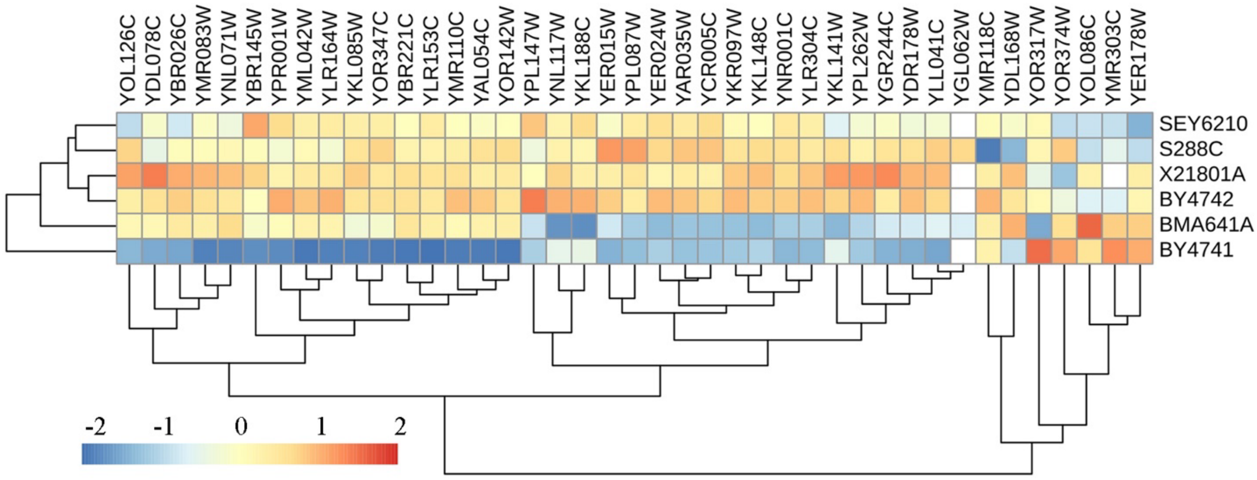

Supplementary Figure 27: Clustering of DEGs of EtOH buffering model.

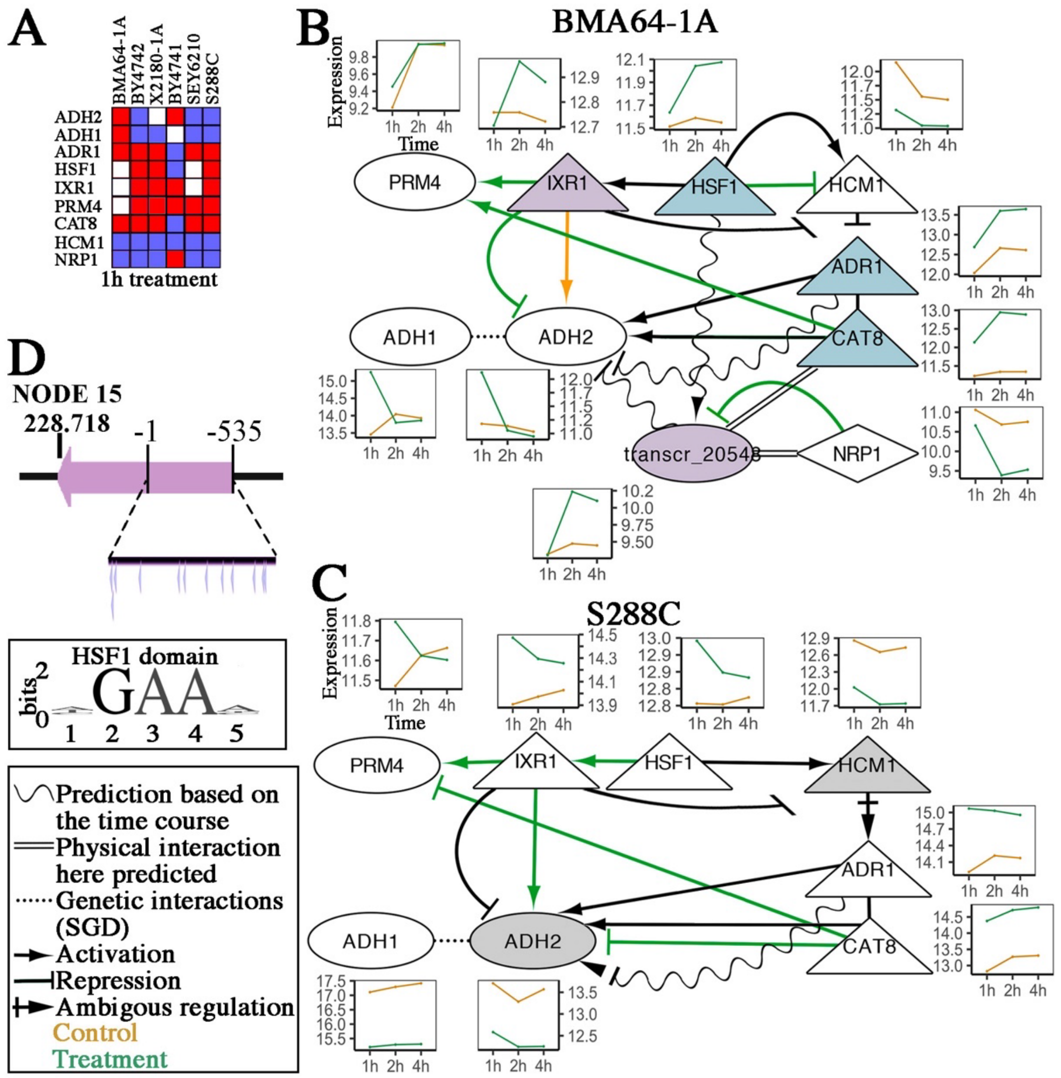

Supplementary Figure 28: Subnetworks of BMA64-1A and S288C with genes from diauxic shift pathway, EtOH buffering model, and the lncRNA transcr_20548 of BMA64-1A.
A: expression of genes including in this graph. Blue and red squares mean down-regulation and up-regulation, respectively; **B-C:** subnetwork of BMA64-1A and S288C; **D:** the locus of lncRNA transcr_20548 and the identification of HSF1 motifs (tiny purple bars); The box in the bottom left indicates the edges types and sources. The edges colors correspond to inferences based on the time-course data. The nodes sharing the same color have similar transcriptional profiles in the time-course.

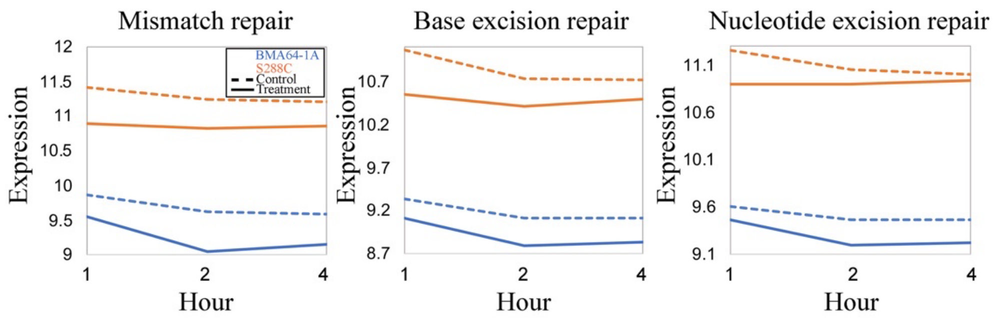

Supplementary Figure 29: Time-course expression profile of genes related to the DNA repair.

Supplementary Tables

Strain	EtOH tol. (%)	Aces. num.	MAT locus	Phenotype
BMA64	30	Euroscarf/20000D	a/alpha	-
*,1,2BMA64-1A	30	Euroscarf/20000A	a	HT
BMA64-1B	26	Euroscarf/20000B	alpha	HT
*BY4741	22	Euroscarf/Y00000 SGD/BY4741	a	LT
*BY4742	26	Euroscarf/Y10000 SGD/BY4742	alpha	HT
BY4743	26	Euroscarf/Y20000	a/alpha	-
CEN.PK2	18	Euroscarf/30000D	a/alpha	-
CEN.PK2-1C	20	Euroscarf/30000A	a	LT
FY1679	22	Euroscarf/10000D SGD/FY1679	a/alpha	-
*,2S288C	20	NBRP/BY20118 SGD/S288c	alpha	LT
*SEY6210	20	NBRP/BY3553 SGD/SEY6210	alpha	LT
W303	22	NBRP/BY4502 SGD/W303	a/alpha	-
*X2180-1A	24	NBRP/BY21559 SGD/X2180-1A	a	HT

Supplementary Table 1: Strains description and results of EtOH tolerance.
*: strains selected for the further analysis; 1: strain with the genome sequenced in this paper. 2: strains subjected to the time course experiment.

Value	N50	Contigs/scaffolds	GC%	Genome size	Genes	AS
Minimum	1,437	17	37.93	126,580	1,744	0.054
Maximum	924,585	27,639	38.34	12,162,499	5,872	0.63
*Selected	202,259	683	38.06	11,880,801	5,668	0.29

Supplementary Table 2: Metrics of BMA64-1A genome considering all assembling.
: metrics of selected assembling; AS: assembling score. Details about the metrics of each 201 assemblies performed is described in the **Supplementary Data 10.

Strain or group	SDH P-value	Cell viability P-value
BMA64-1A (HT)	0.002*	3.9E-13*
BY4742 (HT)	0.001*	2.2E-16*
X2180-1A (HT)	0.205	2.2E-16*
BY4741 (LT)	0.496	0.8554
S288c (LT)	0.097	0.0015*
SEY6210 (LT)	0.718	2.6E-07*
All HTs	0.025*	2.2e-16*
All LTs	0.135	0.0195*

Supplementary Table 3: Statistical analysis of cell viability and SDH assays comparing control *vs* treatment.
*: statistically significant differences (p-value <0.05). The SDH and flow cytometry data are in the **Supplementary Data 8**.

Strain	Group	Up-reg (*%)	Down-reg (*%)	Total
BMA64-1A	HT	1319 (51.54)	1240 (48.45)	2559
BY4742	HT	2050 (49.19)	2117 (50.80)	4167
X2180-1A	HT	2144 (50.21)	2126 (49.78)	4270
S288C	LT	2277 (47.15)	2552 (52.84)	4829
BY4741	LT	2116 (48.39)	2256 (51.6)	4372
SEY6210	LT	1640 (47.74)	1795 (52.25)	3435

Supplementary Table 4: The number of genes significantly differentially expressed.
*: percentages considering only the sum of DEGs.

Up-regulated	Down-regulated
**Enriched terms within HTs**	
**eisosome assembly	**cell wall macromolecule metabolism
coenzyme metabolism	
catabolism	
hydrogen peroxide metabolism	
generation of precursor metabolites and energy	
cofactor metabolism	
carbohydrate metabolism	
***Enriched terms within LTs**	
**import to cell	**membrane glycosylation
**cell communication	**protein folding
**pyridine-containing compound metabolism	**methylation
**response to stimulus	
**autophagy	
catabolism	
hydrogen peroxide metabolism	
generation of precursor metabolites and energy	
carbohydrate metabolism	
cofactor metabolism	
coenzyme metabolism	
*****HT and LT Intersection**	
energy derivation by oxidation of organic compounds	U4 snRNA 3prime−end processing
cofactor metabolism	ribonucleoprotein complex biogenesis
carbohydrate metabolism	
generation of precursor metabolites and energy	
cellular carbohydrate metabolism	
mitochondrion organization	
response to oxygen−containing compound	
*****Exclusive HTs**	
energy derivation by oxidation of organic compounds	double−strand break repair
response to toxic substance	DNA conformation change
oxidoreduction coenzyme metabolism	chromosome segregation
pyridine−containing compound metabolism	biological regulation
protein refolding	cellular component biological regulation organization or biogenesis
*****Exclusive LTs**	
transmembrane transport	tRNA metabolism
cell growth	RNA transport

Supplementary Table 5: Summarization of GO terms of DEGs.

:** BY4741 was not considered; *:** terms not shared between phenotypes; *****:** terms summarized by REVIGO of genes present in the **Supplementary Figure 3**.

Strain	FDR	KEGG number	Pathway
BMA64-1A	6E-05	sce03050	Proteasome
BMA64-1A	1E-04	sce00020	*Citrate cycle (TCA cycle)
BMA64-1A	1E-04	sce00190	*Oxidative phosphorylation
BMA64-1A	1E-09	sce04111	Cell cycle - yeast
BY4742	9E-08	sce00020	*Citrate cycle (TCA cycle)
BY4742	2.E-05	sce00190	*Oxidative phosphorylation
BY4742	3E-32	sce03010	Ribosome
BY4742	7E-14	sce03008	Ribosome biogenesis in eukaryotes
BY4742	4E-08	sce03013	RNA transport
BY4742	3E-07	sce00240	Pyrimidine metabolism
BY4742	9E-07	sce00230	Purine metabolism
BY4742	1E-06	sce03020	RNA polymerase
BY4742	1E-05	sce04111	Cell cycle - yeast
BY4742	3E-04	sce00100	Steroid biosynthesis
BY4742	8E-04	sce00970	Aminoacyl-tRNA biosynthesis
BY4742	13-03	sce03030	DNA replication
X2180-1A	6E-08	sce00020	*Citrate cycle (TCA cycle)
X2180-1A	1E-05	sce00190	*Oxidative phosphorylation
X2180-1A	1E-30	sce03010	Ribosome
X2180-1A	7E-14	sce03008	Ribosome biogenesis in eukaryotes
X2180-1A	2E-08	sce03013	RNA transport
X2180-1A	3E-06	sce03020	RNA polymerase
X2180-1A	2E-05	sce00240	Pyrimidine metabolism
X2180-1A	5E-05	sce00230	Purine metabolism
X2180-1A	1E-04	sce04111	Cell cycle - yeast
S288C	5E-07	sce00190	*Oxidative phosphorylation
S288C	2E-06	sce00020	*Citrate cycle (TCA cycle)
S288C	9E-69	sce03010	Ribosome
S288C	2E-12	sce03008	Ribosome biogenesis in eukaryotes
S288C	2E-07	sce03013	RNA transport
S288C	4E-07	sce03020	RNA polymerase
S288C	2E-06	sce00230	Purine metabolism
S288C	2E-06	sce00240	Pyrimidine metabolism
S288C	6E-04	sce00100	Steroid biosynthesis
SEY6210	9E-06	sce00020	*Citrate cycle (TCA cycle)
SEY6210	6E-43	sce03010	Ribosome
SEY6210	6E-12	sce03008	Ribosome biogenesis in eukaryotes
SEY6210	1E-06	sce00240	Pyrimidine metabolism
SEY6210	1E-06	sce00230	Purine metabolism
SEY6210	1E-06	sce03013	RNA transport
SEY6210	5E-06	sce03020	RNA polymerase
SEY6210	8E-04	sce00513	Various types of N-glycan biosynthesis
**	**	sce00330	Arginine and proline metabolism
**	**	sce00480	Glutathione metabolism
**	**	sce00410	beta-Alanine metabolism
**	**	sce02010	ABC transporters

Supplementary Table 6: Significant KEGG pathways.
FDR: false discovery rate adjusted p-value; ***:** pathway shared among all strains; ****:** maps where spermidine is present.

Biotype	BMA64-1A	BY4742	BY4741	S288C	SEY6210	X2180-1A
Sense	0.00%	0%	0%	0.77%	0%	0%
Antisense	12.77%	3.4%	5.43%	18.92%	6.67%	3.45%
Intronic	0%	0%	0%	0%	0%	0%
lincRNA	0.44%	0%	0.77%	0.39%	0%	0%
Other	86.78%	96.6%	93.8%	79.92%	93.33%	96.55%
**Total**	**227**	**147**	**129**	**259**	**120**	**87**

Supplementary Table 7: Number of lncRNAs per biotype and strain after filtering.
We highlight that there was no “intronic” biotype, as expected (Novačić et al. 2020). The Blastn results comparing our lncRNAs against thousands of other ncRNAs is in the **Supplementary Data 13**.

DE/Strain	BMA64-1A	BY4742	BY4741	S288C	SEY6210	X2180-1A
**Down**	10	7	13	27	14	9
**Up**	19	26	51	47	21	17

Supplementary Table 8: Number of significant differentially expressed lncRNAs.

Strain	*Num. lncRNAs	Num. target proteins	Protein per lncRNA	Num. edges (connections)
BMA64-1A	36	159	4.4	244
BY4742	25	200	8	369
X2180-1A	17	86	5	153
BY4741	17	230	13.5	286
SEY6210	20	174	8.7	233
S288C	44	394	8.9	706
**AVERAGE**	**26.5**	**207.16**	**8.1**	**331.83**

Supplementary Table 9: Summary of lncRNA-protein interactions (LNCPI).
***:** number of lncRNAs with at least one interaction. **Supplementary Data 5.**

Group	Num. edges	Num. nodes	Density	Transitivity	Diameter	Avg path lengh	Ac
BMA64-1A control	150,306	4,808	0.013	0.189	5	2.421	-0.087
BMA64-1A treatment	129,636	4,695	0.011	0.156	6	2.407	-0.109
X2180-1A control	107,316	3,415	0.018	0.198	5	2.360	-0.086
X2180-1A treatment	40,816	3,116	0.008	0.115	6	2.711	-0.097
BY4742 control	104,525	3,498	0.017	0.186	5	2.374	-0.092
BY4742 treatment	38,775	3,067	0.008	0.119	6	2.759	-0.082
S288C control	143,186	4,319	0.015	0.211	5	2.435	-0.079
S288C treatment	44,446	3,672	0.006	0.102	7	2.792	-0.100
SEY6210 control	130,385	3,926	0.016	0.188	5	2.351	-0.087
SEY6210 treatment	56,823	3,501	0.009	0.114	5	2.619	-0.096
BY4741 control	78,877	3,432	0.013	0.133	5	2.362	-0.125
BY4741 treatment	65,086	3,192	0.012	0.154	6	2.515	-0.102
HT control	79,133	2,943	0.018	0.196	5	2.392	-0.080
HT treatment	24,713	2,448	0.008	0.123	7	2.826	-0.080
LT control	45,609	2,209	0.018	0.176	5	2.395	-0.114
LT treatment	11,583	1,682	0.008	0.133	7	3.145	-0.091

Supplementary Table 10: Topological features of all integrated networks.

Ac: assortativity coefficient. **Green:** increased values under treated condition; **Red:** reduced values under treated condition.

Strain	ECC	K	EIG	BET	CLOS	PR	S	HS	C	SC	BC	T	K_{nn}
LT	+	-	-	+	-	+	-	-	-	-	+	-	-
HT	+	-	-	+	-	+	-	-	-	-	+	-	-
BMA64-1A	+	-	-	-	+	NS	-	-	-	-	+	+	NS
X2180-1A	+	-	-	+	-	+	-	-	-	-	+	-	-
BY4742	+	-	-	+	-	+	-	-	-	-	+	-	-
S288C	+	-	-	+	-	+	-	-	-	-	+	-	-
BY4741	+	-	NS	+	+	+	-	NS	-	-	+	-	-
SEY6210	+	-	-	+	-	+	-	-	-	-	+	-	-

Supplementary Table 11: Statistical differences of topological features of network's nodes comparing treatment vs control.
ECC: eccentricity; **K:** number of connections (degree); **EIG:** eigenvectors; **BET:** betweenness; **CLOS:** closeness; **PR:** page rank; **S:** strength; **HS:** hub score; **C:** coreness; **SC:** subgraph centrality; **BC:** burtconstraint; **T:** transitivity; **K_{nn}:** Average nearest neighbor degree; **+:** significant increasing; **-:** significant decreasing**;** **NS:** not significant difference.

Strain	Low EtOH level	Mid EtOH level	High EtOH level
BMA64-1A	-0.961 (<0.001)	-0.873 (<0.0001)	-0.799 (<0.01)
S288C	-0.968 (<0.0001)	-0.988 (<0.005)	-0.810 (<0.01)

Supplementary Table 12: Pearson correlations between relative pH and K under different EtOH stress conditions.

The value of ρ is outside the parenthesis (p-value). The low, mid and high EtOH levels were 5%, 10% and 15% for S288C and 8%, 16% and 23% for BMA64-1A.

Assay	SDH			ROS			DNA damage		
Group	AVG C	AVG T	Rate T/C	AVG C	AVG T	Rate T/C	AVG C	AVG T	Rate T/C
HT	3.65	4.75	1.3	64.45	79.43	1.23	10.02	19.55	1.95
LT	2.88	3.25	1.13	52.52	60.39	1.15	8.46	21.57	2.55
Rate HT/LT	1.27	1.46	-	1.23	1.32*	-	1.18	0.91	-

Supplementary Table 13: Fold-change values from the ROS, SDH, and DNA damage assays.
AVG: average of replicates; **C:** control; **T:** treatment; ***:** Statistically significant differences (p-value <0.05). T/C >1 indicates that the treatment value is higher than the control. HT/LT >1 indicates that HTs have a higher percentage of cells under that particular condition. Orange cells are comparisons with largest values within groups (T vs C or HT vs LT).

Up-abundants		Down-abundants	
**Protein**	**Fold-change**	**Protein**	**Fold-change**
		**HT**	
Pyruvate kinase	1.4	Cytoplasmic ATPase ribosome associated (SSB2p)	0.8
Glycerate phosphate dehydrogenase	1.2	Mitochondrial ATPase	0.8
Enolase 2	2.6		
Phosphoglycerate kinase	1.7		
Alcohol dehydrogenase 1 (ADH1)	1.8		
Phosphoenzyme Pdc1p	1.9		
Chaperone	1.4		
Phosphoglycerate mutase	1.2		
Elongation factor complex eef1a:eef1ba	1.5		
Heat shock protein	1.4		
Elongation factor EF-2	1.1		
Phosphoglycerate dehydrogenase	1		
Enolase 1	1.4		
ATP-dependent molecular chaperone HSC82	5		
Ribosomal protein 80S	1		
Fructose-1,6-bisphosphate aldolase (fructose-bisphosphate aldolase)	2		
Elongation factor EF-3	1		
Heat shock protein 104	1		
		**LT**	
Pyruvate kinase	1.2	Glycerate phosphate dehydrogenase 3	0.9
Enolase 1	2.4	Mitochondrial ATPase	0.7
Heat shock protein	2.4	Heat shock protein	0.8
Phosphoglycerate mutase	1.6	Fructose-1,6-bisphosphate aldolase (fructose-bisphosphate aldolase)	0.4
Elongation factor 2	2.1	Ribosomal protein 80S	0.7
Elongation factor 3	1.6	Phosphoenzyme Pdc1p	0.8
Ribosomal protein 80S	1.3	Elongation factor complex eef1a:eef1ba	0.9
Enolase 2	1.6	Cytoplasmic ATPase ribosome associated (SSB2p)	0.9
Eukaryotic translation factor eIF-5A	2.3	Phosphoglycerate kinase	0.8
		Alcohol dehydrogenase 1 (ADH1)	0.9

Supplementary Table 14: Overview of DAPs.
The down-abundant DAPs had FC >0 and <1, whereas DAPs with FC >1 were considered the up-abundant ones.

Stressor	HT up	HT down	LT up	LT down	HT and LT up	HT and LT down
**osmotic**	GPD1 (YDL022W)	PBS2 (YJL128C), MSB2 (YGR014W), GPP2 (YER062C)	-	SSK22 (YCR073C), GPD2 (YOL059W)	-	STE20 (YHL007C)
**heat**	SSE2 (YBR169C), KAR2 (YJL034W), ECM10 (YEL030W), CDC37 (YDR168W), AHA1 (YDR214W), CPR6 (YLR216C)	-	-	SSE1 (YPL106C)	SSC1 (YJR045C), STI1 (YOR027W)	-
**oxidative**	SOD1/2 (YJR104C/sod1p, YHR008C/sod2p), CTA1 (YDR256C), GPX1 (YKL026C), AHP1 (YLR109W), PRX1 (YBL064C), TRX2/3 (YGR209C, YCR083W), GSH1 (YJL101C)	-	-	-	NTH1 (YDR001C), CTT1 (YGR088W), TSA2 (YDR453C), TRR2 (YHR106W)	-
**ethanol**	HSP12/26/30/82/104 (YFL014W, YBR072W, YCR021C, YPL240C, YLL026W), SSA3/4 (YBL075C, YER103W), TPS1/2 (YBR126C, YDR074W)	MSN2 (YMR037C), TRP2/5 (YER090W, YGL026C), TAT2 (YOL020W)	-	-	-*	HSP78 (YDR258C), TRP3 (YKL211C)

Supplementary Table 15: List of DEGs in our data responsive to many stressors.

Gene	Equation
Hcm1	$\frac{d[Hcm1]}{dt} = ks_{hcm1} - kd_{hcm1} \times [Hcm1] - ki_{hsf1} \times [Hsf1]$
Cat8	$\frac{d[Cat8]}{dt} = ks_{cat8} - kd_{cat8} \times [Cat8] - kd_{lnc} \times [LncRNA] \times [Cat8]$
Ixr1	$\frac{d[Ixr1]}{dt} = ks_{ixr1} + kss_{hsf1} \times [Hsf1] - kd_{ixr1} \times [Ixr1]$
Adr1	$\frac{d[Adr1]}{dt} = ks_{adr1} \times [Adr1] + kss_{hcm1} \times [Hcm1] - kd_{adr1} \times [Adr1]$
Adh2	$\frac{d[Adh2]}{dt} = ks_{adh2} + kss_{cat8} \times [Cat8] + kss_{adr1} \times [Adr1] - ki_{ixr1} \times [Ixr1] - kd_{adh2} \times [Adh2]$
Lnc	$\frac{d[Lnc]}{dt} = ks_{lnc} + kss_{hsf1} \times [Hsf1] - ka_{lnc} \times [Cat8] \times [Lnc] - ki_{nrp1} \times [Nrp1] - kd_{lnc}$
Hsf1	$\frac{d[Hsf1]}{dt} = ks_{hsf1} - kd_{hsf1} \times [Hsf1]$
Nrp1	$\frac{d[Nrp1]}{dt} = ks_{nrp1} - kd_{nrp1} \times [Nrp1]$
Prm4	$\frac{d[Prm4]}{dt} = ks_{prm4} + kss_{cat8} \times [Cat8] + kss_{ixr1} \times [Ixr1] + kd_{prm4} \times [Prm4]$

Supplementary Table 16:Ordinary differential equations (ODEs) used to simulate the dynamic network of BMA64-1A.

Lnc information is referent to the lncRNA transcr_20548 of BMA64-1A. The equations were designed based on the transcriptome time-course data.

Parameter	Gene								
	Hcm1	Cat8	Adr1	Adh2	Ixr1	Nrp1	Lnc	Prm4	Hsf1
ks	0.0013	0.0013	0.0013	0.0013	0.0013	0.0013	0.0013	0.0013	0.0013
kss	0.0041	0.0041	0.0041	-	0.0041	-	-	-	0.0041
ka	-	-	-	-	-	-	0.66	-	-
ki	-	-	-	-	0.002	0.002	-	-	0.002
kd	0.002	0.002	0.002	0.002	0.002	0.002	0.002	0.002	0.002

Supplementary Table 17: Parameters used in the ordinary differential (ODE) equations.
ks: the basal synthesis rate; **kss:** the synthesis rate by transcription factors; **ki:** the inhibition rate; **kd:** the degradation rates; **ka:** the association rate to form complexes. Lnc information is referent to the lncRNA transcr_20548 of BMA64-1A.

Target-protein	Num. of lncRNAs linking the selected proteins	
	BMA64-1A	S288C
YLR106C	18	28
YLL040C	7	-
YNL054W	5	-
YER008C	-	11
YDR150W	-	9

Supplementary Table 18: Target-proteins selected for the secondary structure comparison.

Strain/Plasmid	Locus	Primer	Amplicon size bp
BMA64-1A	transcr_15880	F 5’ GTAACGAGCTGTCAAAACGGA 3’<br>R 5’ TCCAAGATTCCGCGCTTTCA 3’	109
BMA64-1A	transcr_12954	F 5’ ACGGTGCCCTTTGATCCTTC 3’<br>R 5’ AGCGTTGGAACCTCTGTTAAGAT 3’	93
S288C	transcr_17116	F 5’ TACAAGGCAACATAGCAGCG 3’<br>R 5’ AGACCCTGATAGTAATCGTTTCG 3’	72
S288C	transcr_14067	F 5’ CTCCGCTCTCGTATTTCCCT 3’<br>R 5’ ACAGTGGTGAGAATTAGCAAGG 3’	70
S288C	transcr_14670	F 5’ CGGGCCCCGGAACTTTATAA 3’<br>R 5’ TACTCCTTTGGGGTGGGTCC 3’	78
SEY6210	transcr_4844	F 5’ GCCAGGTGTTTCCGTTAGGTA 3’<br>R 5’ TTGCTTGCGTGTCTATGCTG 3’	118
SEY6210	transcr_3536	F 5’ CAATACGGCCATGTTGAGCG 3’<br>R 5’ GTGCTGGCCTCGATAGGTAC 3’	96
S288C	transcr_28768	*F 5’ GCGAGGCATGATATGGACCA 3’<br>*R 5’ TGGTTTTGCAACTTCGCTCG 3’	2,114
-	YFL039C	F 5’ AATCACCGCTTTGGCTCCAT 3’<br>R 5’ AGAACCACCAATCCAGACGG 3’	85
All strains	YOL086C	*F 5’ CATTGCCAGTTAAGCTACCATTAGTC 3’<br>*R 5’ TACCTTCACCACCGTCAATACC 3’	453
pMEL16	pMEL16 oliB; gDNA pMEL16	‡R 5’ GATCATTTATCTTTCACTGC 3’	-
SEY6210	transcr_3536; gDNA pMEL16	‡F 5’ TCTGGTCAACGCGTTGCAGAGTTTTAGAGCTAGAAATAGC 3’	-
SEY6210	transcr_3536; Repair DNA for CRISPR-Cas9	F 5’<br>AATATCCCAACCAGCCATTATTCCAATTATATATTGTTAGTTATAATGATGTTTCTAG<br>AACCTGCATAATACATATGCAAAACTCATGTGTTTCCTAATGCCTGTCTTGTATCA<br>TCAAGG 3’<br>R 5’<br>CCTTGATGATACAAGACAGGCATTAGGAAACACATGAGTTTTGCATATGTATTATG<br>CAGGTTCTAGAAACATCATTATAACTAACAATATATAATTGGAATAATGGCTGGTTG<br>GGATATT 3’	-
BMA64-1A	transcr_20548; gDNA pMEL16	‡F 5’ TTTTCGAGTTCAACAGCGTGGTTTTAGAGCTAGAAATAGC 3’	-
BMA64-1A	**transcr_20548	F 5’<br>CGTGATATACAGTGACAGCCTCACAAATTTTTTTTTCTGAAAAGTCAGAAAGAAAT<br>ATGTAAAAAAAAAAGCAGGAGGCACCTAATTCTGACAACACCGATACCTTTAATTG<br>TGCCAGCTTT 3’<br>R 5’<br>AAAGCTGGCACAATTAAAGGTATCGGTGTTGTCAGAATTAGGTGCCTCCTGCTTTT<br>TTTTTTACATATTTCTTTCTGACTTTTCAGAAAAAAAAATTTGTGAGGCTGTCACTG<br>TATATCACG 3’	-
BMA64-1A	‡‡transcr_20548	F 5’ CCGGACAGTTTGCTTCATGG 3’<br>R 5’ CGCGATAACTCTCCTACCCC 3’	WT = 1,983; Mut = 567
BY4742	Transcr_10027; gDNA pMEL16	‡F 5’ ACTATAGCCTTCAACAGCAAGTTTTAGAGCTAGAAATAGC 3’	-
BY4742	**transcr_10027	F 5’<br>GAATTGAACCCCGATCTGGCACGCGACAAGCGCCCATTCTGACCATTAAACTAT<br>CACGGAGGGTTCTGCTCAGATTTCGTTAAATACCCGGACCATCGTAGATGATTGT<br>TTTTACTCTCC 3’<br>R 5’<br>GGAGAGTAAAAACAATCATCTACGATGGTCCGGGTATTTAACGAAATCTGAGCAG<br>AACCCTCCGTGATAGTTTAATGGTCAGAATGGGCGCTTGTCGCGTGCCAGATCG<br>GGGTTCAATTC 3’	
BY4742	‡‡transcr_10027	F 5’ TTATCCCGATTTGTACCCAGG 3’<br>R 5’ AACAATGATGGTTCGTTCCGG 3’	WT = 2,170; Mut = 455
BMA64-1A	IXR1; gDNA pMEL16	‡F’ 5’ GTACTTGTCTCAGCTCCCACGTTTTAGAGCTAGAAATAGC 3’	-

BMA64-1A	**IXR1	F’ 5’ ATCCCCTTCTTCTATCCATTCTGTGATATACGTACGACGCTAACAGTACCCACAACTGCACAAATAAACAACTTTAGTTTTCCACTGTAACATTATCCCACGCAAACAACGAGAATAAGG 3’<br>R 5’ CCTTATTCTCGTTGTTTGCGTGGGATAATGTTACAGTGGAAAACTAAAGTTGTTTATTTGTGCAGTTGTGGGTACTGTTAGCGTCGTACGTATATCACAGAATGGATAGAAGAAGGGGAT 3’	-
BMA64-1A	‡‡IXR1	F 5’ CTCCCCTTGGTGAGAGAACG 3’<br>R 5’ TTTTGGCTTCATCACGCTCG 3’	WT = 2,479; Mut = 686
BMA64-1A	CTA1; gDNA pMEL16	‡F’ 5’ TTGAAAAATTTAGATGCATA<u>GTTTTAGAGCTAGAAATAGC</u> 3’	
BMA64-1A	**CTA1	F 5’ TTTAAAAGGTAAGTTAAATAAATATAATAGTACTTACAAATAAATTTGGAACCCTAGAAGAACGCTCAAGTAACAAATGAGTGGCGTTGTTTCCACGACAATTATTTATGATAGTGTGTA 3’<br>R 5’ TACACACTATCATAAATAATTGTCGTGGAAACAACGCCACTCATTTGTTACTTGAGCGTTCTTCTAGGGTTCCAAATTTATTTGTAAGTACTATTATATTTATTTAACTTACCTTTTAAA 3’	-
BMA64-1A	‡‡CTA1	F 5’ GCTCTGTCAAACGTCCTTGC 3’<br>R 5’ GGTTCCCTACTCAGTGCTGC 3’	WT = 2,191; Mut = 645
pMEL16	M13	F 5’ TGTAAAACGACGGCCAGT 3’<br>R 5’ CAGGAAACAGCTATGAC 3’	-

Supplementary Table 19: Primers used in this paper.
:** oligos used for Sanger sequencing; **‡:** 5’ phosphorilated oligos used to modify the pMEL16 plasmid; **‡‡:** check the presence of knock-out; *:** repair DNA; **Underlined:** represents complementary region to the pMEL16 plasmid; **WT:** wild-type; **Mut:** mutant.

Pipeline	ICAP	AG	S+C	Avg. Len.	P (%)	PA (%)	Score
Velvet/Oases	467816	0.18	82010	1792	99.30	99.46	9.43
Trinity	127202	0.26	33581	1161.5	100	99.62	9.19
IDBA	277106	0.07	18999	2144	100	78.65	8.64
rnaSPAdes	973409	0.48	464859	850.5	25.15	99.72	6.31

Supplementary Table 20: Overview of *de novo* re-assembling of S288C’s lncRNAs by using CAP3.
The ICAP, AG, S+C, P, PA, and Score are depicted at **Equation** 7. **Avg. len.:** the average sequence length of CAP3 reassembling.

**Family**	**Name**	**BMA64-1A**	**BY4742**	**X2180-1A**	**S288C**	**SEY6210**	**BY4741**
LTR/Copia	TY1A	1	1	1	1	1	1
	TY1B	1	1	1	1	1	1
	TY1C	1	1	1	1	1	1
	TY4	48	59	50	50	51	50
	TY5	13	14	12	13	11	12
LTR/Gypsy	TY3	70	55	48	52	51	48
	TY3-1p_I-int	1	1	-	1	1	1
	TY3-1p_LTR	15	14	14	14	14	14
LTR/Retrotransposon	TY	413	273	253	270	251	273
	TY2_I-int	62	4	2	14	1	5
	TY2_LTR	179	126	111	95	121	116
Y	YPRIME	54	4	-	19	-	4
**TOTAL**	**-**	**858**	**553**	**493**	**531**	**504**	**526**

Supplementary Table 21: Number of TEs here annotated.

Methods

1. Experimental designing and analysis overview

The general workflow is presented in the Figure 1 of the main text.

2. The ethanol tolerance experiments

Thirteen *S. cerevisiae* strains were obtained from Euroscarf (European *Saccharomyces cerevisiae* Archive for Functional Analysis) and NRBP (National Bioresources Project) (**Supplementary Table 1**).

Each strain was grown in YPD (2% of peptone, 1% of yeast extract, and 2% of glucose) at 30°C for 16 h, and shaken at 200 RPM. Then, 1mL of culture was grown in 20mL of YPD at 30°C shaken at 170 RPM to log phase (~14 h) and stored with glycerol at -80°C until use.

To define the highest EtOH tolerance level for each strain, 1 mL of stock was diluted in YPD to an OD_{600} of 0.2. Then, 100 µL of cells were harvested by centrifugation at 2,000 RPM at 4°C for 5 min. Pellet was resuspended using pre-prepared YPD with different concentrations of EtOH or physiological solution (the treatment and control condition, respectively) in plate-wells. Plates were immediately incubated at 30°C for 1h and shaken at 120 RPM; the EtOH or physiological solution ranged from 2 to 32% (v/v). The content of each plate-well was plated on YPD and incubated at 30°C for 24 h. Visual inspection allowed to determine the highest EtOH tolerance level. Experiments were reproduced in biological triplicates.

The EtOH tolerance results (including treatment and control) of 8 haploid strains were plotted in a matrix, in which “0” and “1” mean the absence and presence of cell growth, respectively. By using the Simple K-means algorithm (k=2) in Weka (Hall et al. 2009) we classified each strain into HT or LT phenotype. A total of 3 LTs (S288C, SEY6210, and BY4741) and 3 HTs (BMA64-1A, X2180-1A, and BY4742) were selected for further analysis (**Supplementary Table 1**).

The 6 selected strains were scaled grown to appropriate volumes according to different experiments, albeit we conserved the proportions, concentrations and experimental conditions of the first EtOH stress experiment; the scaling fits the previous results of plating. The samples for transcriptome and proteomes were frozen at -80°C for biomolecule extractions while for other experiments fresh samples were used. After data integration, the generated hypothesis were tested using mutants generated by CRISPR-Cas9 approach.

3. Cell biology analysis

3.1. Growth curves

The growth curves of selected strains (**Supplementary Table 1**) were investigated in 7 different experiments. For the 1st to 5th experiments, cells in log phase were diluted to OD_{600} of ~0.3-0.6 and the population growth was assessed using the Epoch TM2 plate reader (BioTek®), measuring the OD_{600} at 30°C for 24 h, and shaking each 5 or 10 min. The data were evaluated by the Gene 5 software. For the 6th experiment, population growth starting with OD_{600} of 0.4 was measured along 4 hours using the Asys Expert Plus Microplate Reader (Biochrom) with an absorbance filter of 620nm, and the pH of medium was assessed by the pH meter PG2000 (Gehaka). The initial cells of the six first experiments were collected from stocks and reactivated overnight in YPD at 30°C and 200 RPM. For the 7th experiments, mutants and wild-type stocks (see topic **6. Mutants generation**) were grown overnight in YPD plus 0.2 mg/mL of G418 (G418 was not added for the wild-type) and cells were diluted in YPD to an OD_{600} of 0.3, followed by incubation at 30°C until reaching OD_{600} of 1.0. Then, 400 µL of cells were harvested by centrifugation (2,000 RPM for 2 min) before the experiments described below.

The 1st and 2nd growth experiments were performed in YPD without any stressor. However, the initial cells of the 2nd experiment consisted of 1mL of cells treated with the highest EtOH stress for 1h (the experimental condition of this paper described in the topic **2. The ethanol tolerance experiments**). The cells were harvested by centrifugation at 2,500 RPM for 5 min and the pellet was resuspended in 1mL of YPD. The goal of 1st and 2nd experiments was to assess the population behavior in the best conditions and the growth recovery rate (the population rebound) after the severe stress, respectively. The 3rd experiment was performed in YPD with the highest EtOH tolerance supported for each strain to evaluate the severity of these EtOH concentrations.

The 4th experiment was performed in the same medium as the 3rd experiment, but enriched with 0.1 mM of spermidine (Sigma-Aldrich®, S2626). The goal of the 4th experiment was to assess the impact of spermidine under the most harmful conditions. The 5th experiment had the same conditions of the 3rd experiment and it was used to compare with the 4th data.

The goal of 6th experiment was to assess the cytosol acidification. This experiment was performed only for BMA64-1A and S288C under control condition (30% and 20% of

physiological solution, respectively), and ~1/4, ~1/2, and ~3/4 of EtOH (v/v) from the highest EtOH level supported for these strains; it means 0%, 5%, 10%, and 15% of EtOH for S288C; and 0%, 8%, 16%, and 23% for BMA64-1A. OD_{600} was corrected by the average of OD_{600} from YPD (OD_{600} of cells – (average of OD_{600} pure YPD triplicates)). Then, this OD was normalized by the first time point (**Equation 1**). The relationship between pH and OD_{600} was established according to the first part of **Equation 2** (see PH_t). Then, the pH relative to the first time point was established **Equation 2** (see RPH_t). The normalization by the first time-point for both OD_{600} and pH parameters allowed us to compare each point to the initial condition.

$$COD_t = OD_t - \overline{YOD}, t = \{1,2,\dots,9\}\ and;\ ROD_t = \frac{COD_t}{COD_1}, t = \{1,2,\dots,9\}$$ **Equation 1**

where *COD* is the OD_{600} corrected by the YPD ($\overline{YOD}$) for each *t* (time points 0 min, 30, min, 60 min, 90 min, 120 min, 150 min, 180 min, 210 min, and 240 min). *ROD* is the corrected OD_{600} relative to the first time point.

$$PHOD_t = \frac{PH_t}{COD_t}, t = \{1,2,\dots,9\}\ and;\ RPH_t = \frac{PH_t}{PH_1}, t = \{1,2,\dots,9\}$$ **Equation 2**

where *PH* is the raw pH and *COD* is the one obtained from **Equation 1**. Then, *PHOD* is the relationship between pH and OD_{600} within each time point. *RPH* is the pH relative to the first time point.

The goal of the 7th experiment was to evaluate the population rebound of mutant *vs*. wild-type strains after treatment with an extremely harmful environment. Log-phase pellet cells were diluted in 1,600 µL of pre-prepared solutions of YPD with different percentages of EtOH (32.5%, 35%, 37.5%, 40% and 42.5% of EtOH (v/v)), giving an OD_{600} ~0.25. The content was transferred into 10 mL tubes and immediately incubated at 30°C, and centrifuged at 135 RPM for 1h. After, 1 mL of cells (OD_{600} of ~0.25) were harvested by centrifugation at 2,000 RPM for 2 min and the pellet was diluted in 1 mL of YPD. Then, 200 uL of cells were transferred into each well plate in quadruplicates.

The population growth curves of log stage under time function were modeled for the analysis (for the 6th experiment, it was used the normalized data) to assess whether the growth curve (*K*) under each EtOH stress level statistically diverges from control or wild-type cells, according to each experiment. Then, we used the first-order differential equation

proposed by Pierre Francois Verhulst (**Equation 3**) and the analytical solution shown in **Equation 4**.

$$\frac{dP}{dt} = kP\left(1 - \frac{P}{M}\right)$$ **Equation 3**

$$P(t) = \frac{M}{1 + \left(\frac{1}{P(0)} - \frac{1}{M}\right)Me^{-kt}}$$ **Equation 4**

where *P* is the population through the time *t*, *K* is the growth rate, and *M* represents the maximum number of individuals supported by the environment (the charge support). The model was resolved using MatLab with the confidence interval of 95%. For the 6th and 7th experiments, we calculated *K* for each replicated and converted to log (*K*).

Pearson correlation coefficient (two-tailed) analysis was performed to compare the highest EtOH tolerance *vs. K* parameter for the 1st and 2nd experiments. The Pearson correlation (correlation matrix way, assuming a Gaussian distribution, and using two-tailed T-test with 95% of confidence interval) was also performed to evaluate the relationship between the growth curves (log (*K*)) and medium pH of the 6th experiment. Moreover, for each strain in the 6th experiment, we used ordinary one-way ANOVA (Dunnett test, and no matching or pairing) to compare the mean of log *k* from population in medium with each EtOH percentage vs the one from a population without any EtOH stress. The log (*K*) of mutants *vs.* wild-types of the 7th experiment were compared by two-way ANOVA (Geisser-Greenhouse correction, mixed-effects model, Dunnett or Sídák test, and swap direct comparisons).

3.2. Succinate dehydrogenase assay, flow cytometry analysis, RNA and glycerol yield measurements, glucose influx, and western blot

The next experiments were performed for the 6 selected strains (**Supplementary Table 1**), in both control and treatment conditions (the highest EtOH level tolerated for each strain).

3.2.1. Succinate dehydrogenase (SDH) activity assay

The cells under control conditions and treated conditions were harvested by centrifugation at 1,600xg at 4°C for 3 min. The pellet was washed twice using 2 mL of 1X PBS (137 mM NaCl, 10 mM Phosphate, 2.7 mM KCl, and at pH of 7.4); in the second washing, OD_{600} was measured from 10 µL of cells for later normalization. Then, 75 µL of sodium succinate (4M) and 75 µL of Nitro Blue Tetrazolium (10 mg/mL) were added and

incubated at 37°C under shaking for 30 min; samples were always maintained at ~4°C preceding incubation. 20 µL of 37% formalin was added and samples were chill for 30 minutes to block the labeling reaction. Cells were harvested by centrifugation at 1,600xg for 3 min. The pellet was washed twice using 1 mL of 1X PBS and centrifuged under the same conditions aforementioned. Then, 75 µL of DMSO was added and incubated at 37°C for 1 h. Samples were centrifuged and the supernatant was used to measure the absorbance at 260 nM using the reduced Nitro Blue Tetrazolium. Absorbance values were normalized before comparisons to the negative control and initial OD_{600} obtained for each sample.

3.2.2. Flow cytometry to measure DNA damage, ROS, and cellular viability

The percentage of cells with significant DNA damage, intracellular reactive oxygen species accumulation (ROS), and the number of viable cells in the selected strains was measured using flow cytometry assay. The samples after specific preparations (see below) were immediately measured by the BD FACSCalibur™ analyzer and the data were analyzed using the BD CellQuestPro software (BD Biosciences).

For the DNA damage and ROS assays, cells were harvested by centrifugation at 1,600xg at 4°C for 3 minutes. The pellet was diluted in 10 mL of 70% ethanol, incubated for 10 min, and precipitated by centrifugation for 3 min at 1,600xg. The pellet was washed twice in 10 mL of 50 mM sodium citrate, and centrifuged for 3 min at 1,600xg. The pellet was resuspended in 1 mL of 50 mM sodium citrate, transferred to 1.5mL tapered tube, centrifuged for 3 min at 1,600xg, and the supernatant was discarded. It was added 100 µL of lysis buffer (50 mM Tris, 10 mM EDTA, 1 M sorbitol and 1 U/µL of Lyticase), followed by homogenization and incubation for 10 min at 37°C. After the incubation, 500 µL of 1X PBS was added, centrifuged at 2,500xg for 3 min. The pellet was permeabilized using 100 µL of BD-Cytofix/Cytoperm-Plus-Permeabilization-Buffer (BD Pharmingen, USA) and incubated at 37°C for 30 min. Subsequently, 500 µL of 1X PBS was added, homogenized and centrifuged at 2,500xg for 3 min, followed by a washing with 1 mL of 1X PBS. The pellet was resuspended in 300 µL of 1X PBS, and 100 µL of sample was collected as an autofluorescent negative controls. Then, 5 µL of Alexa Fluor® 647 Mouse Anti-H2AX (BD Pharmingen, USA) (for the DNA damage assay), or 1 µL of CellROX™ Green Reagent (Thermo, USA) (for ROS assay), was added to the remaining 200 µL. Samples were incubated for 30 min at 37°C for ROS assays, or for 20 min at room temperature for DNA damage assay. The samples were centrifuged at 2,500xg for 3 min and the supernatant was discarded. The pellet was resuspended and washed three times in 500 µL 1X PBS, and the

pellet was resuspended in 400 µL 1X PBS. Samples were immediately measured by the flow cytometry platform. For ROS assay, the percentage of cells accumulating ROS were compared by using the T-test and fold-changes.

For cell viability assay, the number of viable cells (AnnexinV- and PI-, hereafter referred to as LL), cells under initial apoptosis (AnnexinV+ and PI-, hereafter referred to as LR), and cells in advanced death (Annexin- and PI+, and Annexin+ and PI+, hereafter referred to as UL and UR, respectively), were measured using the kit Annexin V FITC Apoptosis Detection (BD Pharmingen, USA). 1 mL of fresh cells were washed in cold 1X PBS and diluted to 1 x 10^6 cells. Cells were harvested by centrifugation at 1,600xg, at 4°C for 4 min, the supernatant was discarded, and the pellet was resuspended in 200 µL of 1X binding buffer. The sample was split into two flow cytometer-tubes, one with 500 µL of 1X binding buffer (the negative fluorescent control) and another one with 5 µL of FITC Annexin V and 5 µL of propidium iodate. Samples were incubated for 15 min in dark chamber and 400 µL of 1X binding buffer was added, and immediately measured by the flow cytometry platform. The Chi-squared independent test was applied on the percentages of LL, LR, UL and UR comparing treatment *vs* control considering a P-value <0.01 as significant. The relationship between the averages of LL and UR considering each phenotype (HT and LT) and conditions (treated and control) was accessed by supervised learning of Weka 3 (J48 decision tree algorithm, and pruning) (Hall et al. 2009); the LL and UR were selected for this analysis because they presented the highest variances comparing treatment *vs* control.

3.2.3. RNA yield evaluation

A total of 100 uL of control and treated (1h) cells with an OD_{600} of 1 were fixed in 100 uL methanol and acetic acid solution (3:1) and the contents were placed onto slides. Cells were stained with 5 uL of acridine orange (1 mg/mL), 5 uL of DAPI and covered with a coverslip. Images were acquired using the camera Olympus DP71 coupled to a BX61 Olympus microscope using the Texas Red (to assess the brightness of nucleus and cytoplasm) and DAPI filters (to track the whole cell).

Images were analyzed using the CellProfiler software (Jones et al. 2008) to measure the brightness (RNA yield) in nucleus, cytoplasm and the whole cell. The values for each strain were inter-slides normalized, followed by control and treatments normalization; in both cases it was used the mean normalization ratio. The normalized values were converted to a Z-score and extreme outliers of each strain were excluded using the Iterative Grubb's

method with an alpha of 5%. Two-way ANOVA test with Fisher's LSD test and swap direction comparisons were applied comparing control *vs* treatment for each strain.

3.2.4. Glucose uptake and glycerol yield

The concentration of D-glucose and glycerol in the medium were obtained at time point 0 and after 1h of treatment and control conditions to assess whether the high EtOH stress influence glucose influx. High performance liquid chromatography (Shimadzu, Kyoto, Japan) was performed to measure the extracellular compounds using an Aminex HPX-87H column at 65°C (Bio-RAD). A refractive index detector (Shimadzu, Kyoto, Japan) was used to measure the concentrations. The mobile phase was 0.005 N H_2SO_4 at a flow rate of 0.55 ml/min. The obtained values were corrected for the glucose in the medium without cells and a replicates average was calculated. The difference between 1h *vs* 0h was calculated within control and treatment, which means that higher values indicate lesser glucose intake or glycerol yield.

3.2.5. Western blot

BMA64-1A and S288C strains were overnight grown in YPD at 30°C, and 200 RPM. Cultures were diluted to OD_{600} of 0.3 in 20 mL of YPD and grown in the same condition mentioned for 1h under treatment or control condition (**Supplementary Table 1**). The content was harvested at 1000 RPM for 1 min and the supernatant was discarded. Proteins were extracted using standard protocol (Zhang et al. 2011) with minor modifications: after LiAc and NaOH treatment, transfer the content to 2 mL tube, centrifuge at 3000 RPM for 1 min, discard the supernatant, and storage at -80°C until use.

The samples were homogenized in the extraction buffer RIPA (Pierce, Rockford, IL, EUA) with protease inhibitor. Samples were harvested at 4000 RPM at 4°C for 20 min to remove the soluble material. Proteins were quantified using the Bradford method (Bradford 1976). A total of 70 µg of proteins of each sample was resolved on 8% denaturing polyacrylamide gel (SDS-PAGE). Protein was transblotted onto a nitrocellulose membrane (Millipore, USA), blocked in 5% non-fat milk. Then, membranes were incubated with the specific primary antibodies anti-DCP1a (sc-100706; Santa Cruz, 1:1000), anti-PABP (sc-166027, Santa Cruz, 1:1000), and anti-eIF4e (sc-9976, Santa Cruz, 1:1000) to assess the mRNA degradation machinery and P-bodies, mRNA holding by stress granules (SGs), and translation stalling by SGs, respectively. After several washes in TBS-T, the blots were incubated with a horseradish peroxidase HRP-conjugated secondary antibodies at room

temperature for 1 hour. Membranes were developed using the Chemiluminescent HRP antibody detection ECL reagent (Amersham, USA). The ECL signals were captured using a CCD camera (ImageQuant LAS 4000 mini®; GE Healthcare™). The integrated optical densities (IODs) of the targeted protein bands were measured using ImageJ software (National Institutes of Health, USA).

The expression levels were normalized to β-actin 42 kDa and the normalized results were expressed in fold change as the mean±SD. The quantification of each replicate was normalized dividing by the average of control within each condition and strain. Then, ordinary one-way ANOVA (Tukey test, and swapping directions for comparisons) was to assess the significance between control vs treatment and between strains.

4. OMICs

The next experiments were performed for the 6 selected strains (**Supplementary Table 1**), in both control and treatment conditions (the highest EtOH level tolerated for each strain); see experimental design in the Topic 2.

4.1. Raw OMICs data obtainment

Total RNA, proteins and metabolites were extracted from cultures under appropriated scaling; fresh samples were used for the metabolites extraction.

The transcriptome and proteomics were obtained for 36 samples (6 strains x 2 (treatment and control) x 3 replicates). The metabolome was obtained for 60 samples (6 strains x 2 (treatment and control) x 5 replicates) plus some technical replicates randomly chosen. The time-course transcriptome was obtained for 18 samples (2 strains x 2 (treatment and control) x 2 times (3 h and 6 h) x 2 replicates + 2 replicates).

The RNA-Seq was performed by the LcScience (Texas, USA) Company using the Illumina HiSeq 4000 (100 nt, paired-end reads and insert size of 24-324 bp); the company ensured the absence of small RNAs (<200 nts). The genome was sequenced by the GenOne (Rio de Janeiro, Brazil) using the Illumina HiSeq 2500 (250 nt, paired-end reads, 1 Gb of throughput and insert size of ~500 bp).

The LC-MS/MS shotgun proteomics were obtained by using the ESIToF mass spectra (MicroQ-ToF III, Bruker Daltonics) coupled to Prominence UFLC (Shimadzu Corporation) chromatography. The sample solubilization, injection, and spectra acquiring

was performed as previously published (Almeida et al. 2019), being the peptides caring for 80 min, and the spectra acquiring with mass ranged 2,000-3,000 Da the only exceptions.

The GC-MS/MS for metabolomics were performed using the gas chromatograph 7890A (Agilent Technologies, Santa Clara, USA) coupled with a Comb-xt Autosampler (Leap Technologies, Carrboro, USA). The injector temperature was 280°C, the septum purge flow rate was 20 mL min-1 and the purge was turned on after 60 sec. The gas helium flow rate through the column was 1 mL min-1, the column temperature was held at 80°C for 2 min, then increased by 15°C min to 305°C, and held there for 10 min. The column effluent was introduced into the ion source of a GC×GC/TOFMS (Pegasus 4D, Leco Corp., St. Joseph, USA) equipped with two columns of fused silica, a first dimension (Agilent DB-5) having a length of 20 m (0.18 mm internal diameter x 0.18 μm film) and the second column (RXT-17) of size 0.96 m (0.10 mm internal diameter x 0.10 μm film). The transfer line and the ion source temperatures were 280°C and 250°C, respectively. Ions were generated by a 70-eV electron beam at an ionization current of 2.0 mA, and 10 spectra s-1 were recorded in the mass range m/z 45–800.

4.2. Biomolecule extraction

For the total RNA extraction, 1.5 mL of frozen cell cultures were washed with physiological solution and the extraction was performed using Trizol after cell wal digestion using Lyticase. The RNA quality was checked by electrophoresis in 1% of agarose gel and Bioanalyzer, and the quantification was estimated by Nanodrop and Qubit. The samples were treated with TURBO DNase (ThermoFisher) and stored at -80°C until sequencing. Before sequencing, it was performed rRNAs depletion.

The BMA64-1A strain did not have its genome sequenced. Then, the DNA was extracted using Phenol-Chloroform and the quality and quantity were estimated using the same equipment mentioned.

The total proteins were extracted according to the protocol 7 previously published by our group, a protocol specific for MS/MS shotgun proteomics in yeast (Almeida et al. 2019). The extraction quality was checked by using SDS-PAGE. The extracted proteins were digested in 50 mM of ammonium bicarbonate, starting with reduction using 10 mM dithiothreitol and alkylation using 40 mM of iodoacetamide. The proteins were digested using trypsin at a concentration of 1:50 (w:w enzyme:substrate) in 50 mM ammonium bicarbonate buffer at pH 7.8. The digestion was performed at 37°C for 16h and blocked by addition of formic acid at a final concentration of 1% (v/v). The samples were desalinated and purified

using Sep-Pack C18 cartridges (Waters, USA). Lyophilization was performed using Gamma 2-16 LSC (Christ, USA) and samples were immediately frozen at -20ºC until the injection into liquid chromatograph-tandem mass spectrometry (LC-MS/MS) equipment. In all steps LoBind (Eppendorf) tubes were used.

The total metabolite extraction was performed according to Kim *et al.* (Kim et al. 2013). First, 120 mL of fresh cells from EtOH tolerant experiments were immediately split into 5 Falcon tubes with 50 mL with physiological solution. The cells were harvested by centrifugation at 3,000 RPM, and the pellet was resuspended with 10 mL of physiological solution. Cold methanol at-40°C was added into each tube, gently homogenized 3 times, and stand for in quencher (methanol at -40ºC) for 5 min. The cells were harvested by centrifugation at 3,000 RPM at -40˚C, and ~0.2 g of pellet was transferred to microtubes followed by resuspension using 1 mL of methanol and water (50:50, v/v). The next steps were performed three times, starting with samples on ice for 4 min, shacking for 1 min, and finally standing for 30 min at -80˚C. Cells were harvested by centrifugation at 15,000xg for 15 min at 4ºC, and the sample was filtered using a PVDF filter (0.22 mm) followed by lyophilization in vials. The derivatization was performed according to Hoffman *et al*. (Hoffman et al. 2010) using 30 µL of methoxyamine hydrochloride (15 mg mL-1) in pyridine for 16 h at room temperature. The trimethylsilylated was performed by adding 30 µL of N-methyl-N-(trimethylsilyl) trifluoroacetamide (MSTFA) containing 1% trimethylchlorosilane (TMCS), and the mixture stood for 1 h at room temperature. After silylation, 30 µL of heptane was added. Stable isotope reference compounds (1 mg mL-1 each of (13C3)-myristic acid, (13C4)-palmitic acid and (2H4)-succinic acid) were added into samples prior to derivatization, and used as an external standard for quality control. Derivatized samples were analyzed according to Gullberg *et al*. (Gullberg et al. 2004). Blank control samples and a series of n-alkanes (C12–C40), which allowed retention indices to be calculated (Schauer et al. 2005), were also used. Finally, 1 µL of each derivatized sample was injected split-less into the gas chromatography-tandem mass spectrometry (GC-MS/MS) equipment.

5. Bioinformatics

5.1. Genome assembling and annotation of BMA64-1A

The paired-end reads of the BMA64-1A genome were cleaned with Trimmomatic v.0.36 (clip 3:30:10, leading 3, trailing 3, sliding windows 4:15, minimum length 75) and independently submitted to assembling using AbySS v.2.0.2 (Jackman et al. 2017), IDBA

v.1 (Peng et al. 2010), MIRA v.4.0.2, SPAdes v.3.10.1 (Bankevich et al. 2012) and Velvet v.1.2.10 (Zerbino and Birney 2008), varying parameters and assembling strategies (detailed in results section). Moreover, for an individual chromosome assembling, the reads were mapped with Hisat2 v. 2.1.0 (Kim et al. 2015) (default) against the reference genome (S288C version R64-2-1), and the mapped reads were assembled using IDBA v.1 (Peng et al. 2010) (default).

The genomic assembling metrics of each assembling were obtained with QUAST v. 4.5 (Gurevich et al. 2013) (default). QUAST values were normalized generating a score from 0 to 1. Since QUAST generates many metrics for assembling and reference, the score mentioned was calculated for each assembling considering all QUAST-normalized metrics (**Equation 5**); the assembling with score value ≈0 was considered the most similar to the reference genome, and we assumed as the final assembling:

$$d = \sum_{i=1}^{n} \sqrt{(metric - metric_i)^2}$$ **Equation 5**

where *metric* and $metric_i$ are the metric of the reference genome, and of a given assembling, respectively. To obtain *d*, all *n* metrics are considered.

The S288C transcripts from SGD were mapped over the best assembling using GMAP (Wu and Watanabe 2005) (-n1 --min-trimmed-coverage=0.70 --min-identity=0.90), and a *de-novo* annotation was performed with MAKER v.2.32 (Cantarel et al. 2008). A manual curation comparing the two annotations was performed following the rules: 1- annotations present in both evidences were considered correct; 2- regions with disagreements were visually inspected in the reference genome and adjustments were manually performed. Finally, we adjusted the annotations using the results from AGAPE (Song et al. 2015). The protein sequences were translated from annotated regions for further analysis.

<u>5.2. Proteomics analysis</u>

The conversion from mass/charge to peptides was performed by using the Mascot Distiller v2.3.2.0 (Matrix Science) (protein database of *S. cerevisiae* with a total of 6,973 proteins, fixed carbamidomethylation to 57.0215 Da monoisotopic mass, methionine oxidation with a variation of 15,9949 Da monoisotopic mass, 0.1 Da error for ms and ms/ms, score >40 and ion score >30). The quantification of protein abundances was performed by using the software Scaffold Q+ (Proteome Software) based on the spectral counting and

assuming 2 and 88% as the minimum number of peptides and protein coverage, respectively. The differential abundances were calculated comparing treatment *vs* control within phenotypes.

5.3. Metabolomics analysis

ChromaTOF v. 4.51 (Leco Corp., St. Joseph, USA) was used to perform the baseline correction and to export all MS files to NetCDF format. Peak detection, retention time alignment, and library matching were performed using the TargetSearch package (mass range = 45,800, library of MDN35 column from Gold Database, RI_dev = 5, 2.5, 1.25, Top masses = 20, exclude masses = 147-149) (Cuadros-Inostroza et al. 2009). Metabolites with mass count ≥3 were selected, followed by compound identification by comparing their retention indexes (± -2s) and spectra (similarity ≥600) against compounds of Golm-Metabolome-Database (http://gmd.mpimp-golm.mpg.de/download/) (Kopka et al. 2005). If a given metabolite mass score = 0 in ≥3 replicates, it was scored as 0 throughout the replicates. After, each metabolite intensity was normalized (**Equation 6**):

$$Mi = \frac{\left(\frac{M0_i}{\sum TICs}\right)}{Mg_i}$$ **Equation 6**

where *Mi* is the final intensity of metabolite *i*, $M0_i$ is the initial measurement of metabolite *i*, *TICs* is the total ion counting of the sample which the *Mi* is present; Mg_i is the weight in grams of the sample *i*.

After normalization, metabolites were evaluated concerning their relative abundances comparing treatment *vs.* control for each strain using the peak intensity in the MetaboAnalyst v.4.0 (default) (Chong and Xia 2018). Heatmaps (Pearson clustering), principal component analysis (PCA) and partial least squares discriminant analysis (PLS-DA) were generated to check the clustering of controls and treatments. The T-test and PLS-DA was used to calculate the differentially abundant metabolites (DAM) (q-value ≤0.05) of each strain based on the intensities, comparing treatments *vs* control. Moreover, the Log_2 fold-change (L2FC) was used to define the up and down abundant metabolites.

Sets were used to evaluate the DAMs: 1st, the metabolites present in at least two HTs or two LTs were compared to seek metabolites shared by both phenotypes as well as the phenotype-exclusives ones; 2nd, the phenotype-exclusive DAMs present in more than one strain were split into down or up abundant sets; 3rd, the DAMs present in both HT and LT

groups were split into up and down abundant. The metabolite IDs from those 3 strategies were converted to KEGG ID and their L2FC were mapped with transcriptome data onto KEGG pathways (further described).

5.4. Re-editing the annotations before differential gene expression analysis

Repetitive elements and tRNAs were only previously annotated for the S288C strain. Hence, new GFF files for each strain were here created harboring repetitive elements, tRNAs and lncRNAs. We used RepeatMasker (Tarailo-Graovac and Chen 2009) to annotate the repetitive elements, tRNAscan (Lowe and Eddy 1997) to annotate tRNAs, and an *in house* pipeline to identify lncRNAs. The new GFF files were used in the differential gene expression analysis (DGE) are available here https://figshare.com/s/e75fe1be9af623988be1.

5.4.1. Transposable elements and tRNA identification

RepeatMasker (-e crossmatch, -s, –lib repeat_library.fasta, –pa 10, –a, and -gff) was used to find the repetitive content and we used a Perl script from RepeatMasker (*buildSummary.pl*) to summarize the annotations and finding repeat copy numbers. The Ty elements of *S. cerevisiae* were downloaded from RepBase Update 22.09 (20171013) and sequences of Ty3 element from *Saccharomyces paradoxus* (hereafter referred to as TY3-1p_I and TY3-1p_LTR) were also incorporated in the dataset since they are also found in the *S. cerevisiae* genome (Carr et al. 2012). Thus, a repeat library was stablished as input for RepeatMasker (*repeat_library.fasta*).

The repeat landscapes were also created using the alignment file output (.align) from RepeatMasker. The Kimura 2-parameter was calculated using the perl script *calcDivergenceFromAlign.pl* (-s <strain.divsum>, -a <strain.calcDiv> <strain.aln>), a perl script from RepeatMasker; this metric represents the divergence of each alignment against the consensus sequence and it is used to estimate of the relative time of insertion waves of a repeat copy on the genome.

Before performing the DGE, the annotations of TEs were manually curated for all strains. In this case, if two TEs are closer to each other in the genome (1nt), and presented the same name and orientation, the annotation was merged as a single annotation. The “Low-complexity”, “simple repeats” and “rRNA/Fungi” were not annotated.

To annotate the nuclear and mitochondrial tRNAs of all strains (except for the S288C), it was used the tRNAscan-SE (-G, -C, -H). The sequences labeled as

"pseudogenes", "undetermined and suppressors",and "nuclear tRNAs <70 nts size" (the length previously established (Schimmel 2017)) were not included in the annotation.

5.4.2. The in house pipeline to identify the lncRNAs and comparative analysis

The paired-end reads (throughput was ~40 million reads *per* sample) for all RNA-Seq libraries were trimmed using Trimmomatic v.0.36 (Bolger et al. 2014) (clip 2:25:10, headcrop 2, leading 3, trailing 3, sliding windows 4:30, minimum length 95) (**Supplementary Figure 17B**), and used to identify the lncRNAs and to perform the DGE as well (further described).

Here we developed a *pipeline* to assemble lncRNAs. Overall, the filtered reads were mapped over coding sequences of many different species. After, the non-mapped paired-reads were assembled using different algorithms, and a score was calculated to rank the best assemblies. The transcript redundancies within strains were excluded, and the remaining transcripts were mapped over the genome of each strain to exclude spurious assembling. The mapped transcripts that may be coding sequences or mobile elements were excluded, and the remaining sequences were checked again concerning their potential coding (**Supplementary Figure 17**). The detailed pipeline is described below.

A dataset with millions of sequences was creating in order to include: 1- coding sequences (CDS), and proteomes of eukaryotes, and bacteria; 2- genomes; 3- microRNAs precursors; 4- ncRNA families present in Rfam database (Burge et al. 2013), being lncRNAs (Rfam accession number 01884) the only exception; 5- mobile elements (**Supplementary Figure 17A**, **Supplementary Data 14**). The RNA-Seq filtered reads of each strain were independently aligned on nucleotide sequences of the database mentioned using Hisat2 (Kim et al. 2015) (default) (**Supplementary Figure 17A-B**). Then, we assembled the non-aligned read-pairs selected by Pairfq script (The MIT License) (**Supplementary Figure 17C, D**) since the ones are reads without similarity with coding, ncRNAs (excepting lncRNAs), mobile elements, and mitochondrial and contaminants genomes; reads without a member of the pair were excluded of this assembling.

The "Single Assembler Multiple Parameters" strategy (He et al. 2015) was used for the *de novo* assembling mentioned (**Supplementary Figure 17D**); it was performed parameter adjustments for each step using the S288C reads, and then, we applied the best parameter set to independently assembly the reads of all other strains. First, Velvet/Oases (Zerbino and Birney 2008; Schulz et al. 2012), Trinity (Haas et al. 2013), IDBA-tran (Peng et al. 2012) and rnaSPAdes (Bankevich et al. 2012) were independently tested to determine which one was the best assembler for our dataset. For Velvet/Oases, rnaSPAdes and IDBA-

tran parameters, the kmers ranged from 19 to 81, and for Velvet/Oases and rnaSPAdes we had set-up an automatic coverage cutoff and no scaffolding assembling. For Trinity assembler, we set-up kmers ranging from 19 to 31. These softwares were set-up to assemble only transcripts ≥200 nts, when this option was available.

All assembled transcriptomes within each assembler were merged in a single file, sequences with ≥10% of "Ns" (non-identified nucleotides) were removed, followed by a generation of 1 transcriptome per assembler; this re-assembling was performed using CAP3 (Huang 1999) (parameters maximum gap size 5, maximum overlap percentage size 20, clipping stop point 1, percentage of identity cut point 80, and the value of reverse orientation 1) (**Supplementary Figure 17E**). The singlets and contigs from CAP3 were merged, and the filtered reads (the ones used in the first assembling) were mapped over this file using Bowtie2 (default) (Langmead and Salzberg 2012). A score was created (**Equation 7**) to evaluate the final assembling, and the highest value indicates the best transcriptome (**Supplementary Figure 17F; Supplementary Data 13**). The score of **Equation 7** allowed ranking Trinity as the best assembler. As mentioned, after these adjustments of *de novo* assembling pipeline using the S288C reads, the same settings were applied to independently assembling the lncRNAs of all other strains.

$$\{Score = (1 - AG)3 + P \times 3 + \left(\frac{PA \times 4}{100}\right);\ AG = \frac{S+C}{ICAP};\ P = \frac{S \geq 200nts + C \geq 200nts}{S+C}\} \quad \textbf{Equation 7}$$

where *AG* (the assembling gain) is a rate of how many sequences the CAP3 used to assemble. *P* is the percentage of CAP3 assembled sequences ≥200 nts, and *PA* is the percentage of reads aligned using Bowtie2 over CAP3 assembling. *S* and *C* are the number of sequences in the "singlets" and "contigs" CAP3's output, respectively. *ICAP* is the number of sequences with ≤10% of "Ns" (assembled by IDBA, rnaSPAdes, Trinity or Velvet/Oases) used as CAP3 input. *PA* and *P* range from 0 to 100 while *AG* range from 0 to 1. The *AG* rate ≈0 is considered a better value since it expresses that a higher number of input sequences was re-assembled by CAP3. *P* and *PA* ≈1 are considered better values expressing a higher number of large transcripts was assembled by CAP3 and a higher number of reads was used by the first assemblers (the ones before CAP3), respectively.

The set of non-redundant transcripts were found using CD-HIT (Li and Godzik 2006) (identity ≥98%, percentage of alignment ≥99%, -n9, -r1, and coverage ≥99%) (**Supplementary Figure 17G**). These non-redundant sequences of each strain were mapped over its own genomes by using GMAP (Wu and Watanabe 2005) (coverage ≥90%, identity ≥99%, -k15, -B5, p3, no close indels, and no-chimeras) (**Supplementary Figure 17H**). Then, the mapped transcripts were re-checked concerning their potential to be coding,

ncRNAs (except lncRNAs), repetitive elements, or contaminant genomes (viruses, bacteria and mitochondria) using the same datasets listed in **Supplementary Data 12**. For this purpose, the mapped transcripts were: 1- aligned against the proteomes using Blastx (E-value = 0.00001 and 1 alignment *per* query); 2- aligned against the ncRNAs using dcmegablast (E-value = 10E-5, and word-size = 11); 3- the simple repeats and transposable elements were searched by RepeatMasker (crossmatch, similarity = 10, -s, -gccalc, skipping bacterial insertion, and word length = 4,000) (Smit and Hubley); 4- and the transcripts were aligned against bacteria, viruses and mitochondrial genomes using GMAP (coverage ≥50%, identity ≥50%, -k15, -B5, p3, no close indels, and no-chimeras). The data distribution of all these results were analyzed to establish cutoffs to filter out undesirable sequences (**Supplementary Figure 17I**; **Supplementary Figure 18; Supplementary Data 13**).

To verify the lack of coding regions on selected sequence, the filtered transcripts were protein translated (≥10 aa) using the Getorf (Rice et al. 2000) (**Supplementary Figure 17J**) and transcripts/proteins were evaluated using Hmmer (Mistry et al. 2013) (using the Pfam version 31.0 database (Finn et al. 2014)), Tcode (Rice et al. 2000), Portrait (Arrial et al. 2009) and CPC (Kong et al. 2007) (**Supplementary Figure 17K**); Tcode, Portrait and CPC in default mode. The transcripts with motifs fitting Hmm models by using Hmmer and according to cutoffs at **Supplementary Figure 19** and **Supplementary Data 13**, were assumed as potentially coding, whereas the other sequences that fit coding sequences using at least two out of three other programs (Tcode, Portrait or CPC) (according to cutoffs at **Supplementary Figure 19** and **Supplementary Data 13**) were defined as coding sequences. Hence, sequences not found neither by Hmmer nor by two other programs were assumed as putative lncRNAs (**Supplementary Figure 17; Supplementary Data 5**).

The putative lncRNAs were classified according to GENCODE v7: 1- 'lincRNA' is a transcript from an intergenic loci; 2- 'intronic' lies in an intron without exon-overlapping; 3- 'sense' lncRNA surrounds coding gene; 4- 'antisense' is transcribed from the opposite strand of a coding gene and overlaps any exons/introns; 5- lncRNAs out of this classification were labeled as “others” (**Supplementary Figure 20**).

Using the Blastn stand-alone (E-value <1E-5, and query coverage per subject >50%), we queried the lncRNAs here found against 3,898 lncRNAs, CUTs, SUTs, XUTs, and other ncRNAs previously found (Zappulla and Cech 2004; Martens et al. 2005; Hongay et al. 2006; Berretta et al. 2008; Luke et al. 2008; Houseley et al. 2008; Neil et al. 2009; Xu et al. 2009; Camblong et al. 2009; Bumgarner et al. 2009; Huang et al. 2010; Gelfand et al. 2011; van Dijk et al. 2011; Geisler et al. 2012; van Werven et al. 2012; Schulz et al. 2013; Nadal-

Ribelles et al. 2014; Huber et al. 2016; Yu et al. 2016; Kyriakou et al. 2016; Parker et al. 2017, 2018; Till et al. 2018): the ncRNA database can be found here https://figshare.com/s/9689d0046c824d3e1f74.

Moreover, micropeptides-coding lncRNAs might translate short amino acids chains (from 10-99 amino acids). The potential to code micropeptides was assessed using proteomics data of MS/MS (further described). For this purpose, SpectraST (default) implemented in the Trans-Proteomic (TPP) pipeline (Seattle Proteome Center (SPC)) was used, which peptides were analyzed by Peptide Prophet (probability ≤0.05, minimum number of peptides = 7), and further filtered by probability ≥0.6 and the number of peptides ≥2.

We analyzed the structural and functional conservation of yeast's lncRNAs based on lncRNAs-protein interaction (LNCPI) networks (further described). We hypothesize that lncRNAs which bind to the same proteins are structurally conserved (intra or inter-strains). For the intra-strain comparison, proteins targeted by several lncRNAs (hereafter called target-proteins) in both BMA64-1A (HT), and S288C (LT) were selected (**Supplementary Table 18**). Within-strains similarities for the lncRNAs which bind to those proteins were assessed using CD-HIT (Li and Godzik 2006) (identity cutoff of ≥50%). Then, the two most similar lncRNAs per protein were selected for each strain and their secondary structure were modeled and compared in an intra-strain way. For the inter-strain comparison, we compared the structures of lncRNAs present in different strains. First, the conservational level of lncRNAs among strains were assessed using CD-HIT (Li and Godzik 2006) using S288C's lncRNAs as queries. The identity cutoff of ≥80% or ≥90% were chosen, thereby, a lncRNA of a given strain clustering with a lncRNA of S288C were assigned as orthologues. Then, orthologues to the S288C transcr_22854, transcr_24032, and transcr_20180 were selected; these 3 lncRNAs of S288C presented one orthologue in 5, 4, and 3 other strains, respectively (further described). The structures of orthologues were modeled and compared. For the structural analysis, we used CROSSalign (Delli Ponti et al. 2018) (default parameters), and the CROSS Global Score (CGS); the structural distance score (SDS, which values >0.095 are considered different structures) were evaluated.

The validation of some lncRNAs differentially expressed was performed by using qPCR with SYBR Green 2-step (**Supplementary Table 19**), and the ACT1 (YFL039C) as the housekeeping gene. The total RNA was extracted from cells of each 6 selected strains under treated and control conditions (the same parameters used to perform the RNA-Seq depicted in "**2. The ethanol tolerance experiments**") by SV Total RNA Isolation System

(Promega). S288c presents a huge lncRNA at the chromosome IX (the transcr_28768, which has 2,739 bp and it is localized from 336,491 to 339,229) which was validated concerning its size by amplifying this transcript from cDNA followed by Sanger sequencing (**Supplementary Table 19**).

5.5. Differential gene expression, GO enrichment and KEGG pathways mapping

The RNA-Seq filtered reads of each strain were mapped over its own genomes using Hisat2 v.2.10 (Kim et al. 2015) ("--no-softclip"). The reads counting per transcript were obtained using Bedtools multiBamCov v.2.26.0 (Quinlan and Hall 2010), the DGE treatment *vs* control was provided by DESeq2 (Love et al. 2014), and the transcripts (genes, lncRNAs, or TEs) with false discovery rate (FDR) < 0.01 were considered differentially expressed. Gene ontology (GO) enrichment analysis was performed using g:Profiler (Reimand et al. 2016). The enriched terms shared between phenotypes, or phenotype-exclusive were summarized by REVIGO (Supek et al. 2011). The obtainment of DGEs were performed for each strain using the strain-specific GFF annotation mentioned in "**5.4. Re-editing the annotations before differential gene expression analysis**". Moreover, the average of rLog normalized expression in control and treatment of differentially expressed TEs of HTs and LTs, were tested concerning normality by Shapiro-Wilk test. Further, the Wilcoxon test was applied to verify the significance of difference within HT/LT controls and within HT/LT treatments.

The Pathview package (Luo and Brouwer 2013) was used to plot differentially expressed transcripts (DEGs) on KEGG pathways by two approaches: 1st, the DEGs exclusive of each phenotype (HT or LT) were independently plotted, as well as the DEGs shared between HTs and LTs; 2nd, the fold-changes (FCs) of all expressed genes (independent of their FDR) were plotted for each strains, independently. The abundance of selected metabolites (see section "**5.3. Metabolomics analysis**") were also plotted with the transcripts in the same way mentioned. L2FC was submitted to GAGE for enrichment analysis and pathways with FDR <0.01 were considered significantly enriched. Finally, lncRNAs were manually mapped onto KEGG maps after defining the lncRNA-protein interaction networks (LNCPR) for each strain (below described). The most significantly affected KEGG pathways were found considering a normalized rate of down and up-regulated genes (**Supplementary Data 6**).

5.6. Networks analysis

5.6.1. Modeling the KEGG pathway-based networks based on time-course data

We initially integrated 1,175 differentially expressed genes from transcriptomes, the differentially abundant metabolites from metabolomes, and lncRNA-protein networks. To find the essential systems affected by the EtOH, we modeled the selected pathways and integrated the time-course data for glycolysis and gluconeogenesis, TCA cycle, peroxisome, cell cycle, autophagy, MAPK, longevity, protein process in endoplasmic reticulum, RNA transport, ribosome biogenesis, mRNA surveillance, and RNA degradation pathways. All tools used in this section are implemented in Cytoscape (Shannon et al. 2003). The time-course data and these networks are available at https://figshare.com/s/537bb28192e48b8483c5.

To create the pathway integrated network (PINET), each essential pathway aforementioned was graph-modeled as a node hereafter referred to as “node-pathway”. The genes were also modeled as nodes and linked to their node-pathways. The genes present in two pathways links both node-pathways. For instance, the node-pathway “X” had the genes “A”, “B”, “C”, and “D”, whereas the node-pathway “Y” had the genes “C”, “E”, and “F”. Therefore, the PINET would consists to node-pathways “X” and “Y” linked by the node “C”. Moreover, node-pathway “X” would have the additional blunt-ended nodes “A”, “B”, and “D”, whereas the node-pathway “Y” would have the nodes “E”, and “F”. After, one network per phenotype (represented by BMA64-1A and S288C, respectively) were built by using the TiCoNE (Time Course Network Enricher) clustering method (Wiwie et al. 2019). TiCoNE mapped the time-course data of treatment conditions of both BMA64-1A and S288C over the PINET, which was assumed as a scaffold network. For both strains, the largest cluster composed new networks and the ones were compared using the Dynet (Goenawan et al. 2016). The TiCoNE set-up was 10% of objects with low standard variation were removed in the preprocessing step, discretization = 10, clustering by using CLARA method (k = 10), samples = 1, sample size = 762 (the number of nodes in the PINET), the mean time series was used to calculate the prototypes, and iterations were performed by “Until converge” option. Further, we compared the time-course profile (control and treatment) of each pathway’s genes between BMA64-1A and S288C. Based on the average and median of each time-point under the treatment condition, we defined 3 expression profiles: 1- “up and stable” when most of genes increase the expression from 1h to 2h and maintain the expression until 4h; 2- “down and stable” when most of genes decrease expression followed by the maintenance of expression; 3- “stable” when most of genes does not change the expression along the time-course.

The network propagation traces the flow of information throughout a network over time until convergence, overcoming problems to associate genes to phenotypes by using clustering or shortest-pathways approaches (Carlin et al. 2017; Cowen et al. 2017). The network propagation of PINET was achieved for each node-pathway using the diffusion algorithm (Carlin et al. 2017). For this purpose, each node-pathway of each phenotype was independently queried by the diffusion algorithm (Carlin et al. 2017). The highest "heat diffusion" (≥3rd quartile) and the number of edges (>1 edge) were used as a cutoff to select the most relevant nodes of a given network propagation.

We also modeled the DNA damage pathway using information from the literature and our time-course of BMA64-1A and S288C to seek the possible mechanism of DNA damage during the EtOH treatment.

5.6.2. Integrating lncRNAs into networks, statistical analysis of networks, and network dynamic modeling

It was predicted the lncRNAs-protein interaction (LNCPI), considering proteins ≥32 amino acids of each strain using the lncPRO tool (Lu et al. 2013) (default). The probability distributions of interactions follow a normal distribution (data not shown), then the ones ≥0.95 of probability were selected for further analysis. At the end, we had a total of 6 LNCPI.

An integrative network was built merging yeast's experimentally validated protein-protein physical interactions from Biogrid (Chatr-Aryamontri et al. 2013), and MINT (Licata et al. 2012), gene regulatory networks from YTRP database (Yang et al. 2014), and metabolic networks from REACTOME (Fabregat et al. 2014); redundancies between Biogrid and MINT were excluded as well as interactions present in the NEGATOME (Blohm et al. 2014). The integrated network was further merged with each LNCPI generating 6 undirected strain-specific networks (**Equation 8**).

$$\begin{cases} G_k = (V_k, E_k) = LNCPI_k \cup Uninet = A_k \\ G'_k = (V'_k, E'_k) \\ V'_k = \{v_i\} = \{gff_k\} \vee gff_k \subseteq GFF_k \\ E'_k = \{(v_i, v_j)\} \vee a_{ij} = 1 \end{cases} \qquad \textbf{Equation 8}$$

in which G_k is an adjacency matrix A_k (where a_{ij} an element of A_k) of a given strain (k). The G'_k is a subgraph from G_k. V'_k is a subset of nodes v_i from G'_k generated based on GFF annotation (gff_k). E'_k is a subset of edges of V'_k from G'_k. Hence, G'_k are strain-specific networks harboring their respective LNCPI as well.

Based on the expression data, it was created for each G'_k (the strain-specific networks) two networks representing the control and the treatment conditions (**Equation 9**), giving a total of 12 networks:

$$\begin{cases} G'_k = A_k \\ Y = \{y_i\} \vee y_i \subset V'_k, FC_{yi} < 0 \\ Z = \{z_i\} \vee z_i \subset V'_k, FC_{zi} > 0 \\ G_{kc} = (V'_{kc}, E'_{kc}) \vee G_{kc} \subset G'_k \\ V'_{kc} = \{v_i \not\subset Z\} \\ E'_{kc} = \{(v_i, v_j)\} \vee a_{ij} = 1 \\ G_{kt} = (V'_{kt}, E_{kt}) \vee G_{kt} \subset G'_k \\ V'_{kt} = \{v_i \not\subset Y\} \\ E'_{kt} = \{(v_i, v_j)\} \vee a_{ij} = 1 \end{cases}$$ **Equation 9**

where G'_k is the graph specific of a given strain (*k*) from **Equation 8**; G'_k is also an adjacency matrix A_k (a_{ij} an element of A_k). V'_{kc} and V'_{kt} are subsets of nodes v_i from G'_k reflecting nodes of the control and treatment states, respectively. E'_{kc} (edges of control state) and E'_{kt} (edges of treatment state) are subsets of edges of V'_{kc} (nodes of control state) and V'_{kt} (nodes of treatment state), respectively, presented in G'_k. *FC* are the L2FC of each gene significantly differentially expressed of strain *k*.

A single network *per* phenotype (HT and LT) was created for both treatment and control conditions (**Equation 10**).

$$\begin{cases} G_{HTC} = G_{X2180-1A\,C} \cap G_{BY4742\,C} \cap G_{BMA64-1A\,C} \\ G_{HTT} = G_{X2180-1A\,T} \cap G_{BY4742\,T} \cap G_{BMA64-1A\,T} \\ G_{LTC} = G_{SEY6210\,C} \cap G_{S288C\,C} \cap G_{BY4741\,C} \\ G_{LTT} = G_{SEY6210\,T} \cap G_{S288C\,T} \cap G_{BY4741\,T} \end{cases}$$ **Equation 10**

where *HT* is the HT phenotype, *LT* is the LT phenotype, *C* and *T* are the control and treatment states, respectively. The graphs described in the intersections are the variables G_{kc} of G_{kt} calculated by **Equation 9**. At the end, **Equation 8**, **Equation 9**, and **Equation 10** provided a total 16 graphs used in further analysis.

For each graph out 16 networks, it was calculated the normalized DDC function ∏(*k*,*k'*) (a normalized degree-degree correlation). DDC evaluates the likelihood of interaction between two nodes with a degree *k* and *k'* compared to a random network with the same degree distribution, but with uncorrelated degrees; it gives a "microscopic" characterization of networks and evaluates how different they are from a random data (Fernandes et al. 2010). The topological features eccentricity, degree, eigenvectors, betweenness, closeness, page rank, strength, hub score, coreness, subgraph centrality, burt-constraint, transitivity and average nearest neighbor degree were calculated for each node of all networks using the Igraph package (Csardi and Nepusz 2006). The normality test for the metrics mentioned

was performed using Shapiro-Wilk test, and the statistics relevance of differences between controls and treatments was performed using the Kruskal-Wallis test.

The guilt-by-association approach was used to hint lncRNA functions, where the GO terms of target-proteins were analyzed by using g:Convert (selecting *S. cerevisiae* and target namespace "GO") (Raudvere et al. 2019), and summarized by REVIGO (allowed similarity = 0.7) (Supek et al. 2011). The first analysis was performed for all lncRNA's target-proteins (with all redundant proteins) without discriminating neither strains nor differential expression (a total of 1,991 proteins, which gave 22,313 GOs from g:Convert). The second analysis used phenotype-exclusive lncRNA's DE target-proteins; the ones are protein targeted by lncRNAs within all HTs or all LTs. The third analysis was concerning the lncRNA's DE target-proteins considering all strains. The first and third analysis allowed us to assess the functional conservation of lncRNAs responsive to the EtOH stress.

For the lncRNA-propagation analysis, we selected the nodes related to DE up-regulated sequences (L2FC ≥1E-6) of each strain (the G'_k depicted in **Equation 8**). Unconnected nodes or nodes with self-loops were excluded. The network propagation using the diffusion algorithm (Carlin et al. 2017) was assessed starting from each DE lncRNAs. After, the lncRNAs and their first neighbors were selected, and the top 20 ranked nodes from diffusion information composed new subgraphs (the nodes with self-loops or unconnected ones were excluded again). The same processes were performed for lncRNAs and genes down-regulated (L2FC ≤-1E-6).

Our OMICs revealed that autophagy, P-bodies (PBs), RNA catabolic process, stress-granules (SG), proteasome storage granules (PSG), proteasome and regulatory parts, protein polyubiquitination and positive regulation of ubiquitination (PPPR), and protein deubiquitination and negative regulation of ubiquitination (PDNR) are extremely affected by the severe EtOH stress. We found lncRNAs binding to proteins of these structures and pathways mentioned, hence we analyzed the expression profile of these genes and lncRNAs. We also sought which structures and pathways mentioned diverge in terms of lncRNAs from expected by calculating their number of lncRNAs and applying the Student T-test (paired, two-tailed, and confidence level of 95%). The Pearson correlation coefficient (two-tailed, and confidence of interval of 95%) was used to compare the growth rate (*k* parameter described by **Equation 3**, and **Equation 4**) after the severe EtOH stress (the population rebound) and the percentage of the highest EtOH level per strain *vs* the percentage of lncRNAs connecting synergic pathways (structures or pathways that had similar outputs, e.g., proteasome and PPPR, which act on protein degradation). Genes

shared among PB, SG, and PSG were not considered to avoid oversampling. Similarly, proteasome genes were not recounted for PSG.

To better understand the role of lncRNA transcr_20548 of BMA64-1A (an EtOH responsive lncRNA with a direct influence on it), a subnetwork with this lncRNA was built. This subnetwork was conceived based on G'_k of BMA64-1A depicted by **Equation 8**, information from the literature, data from SGD database, and our time-course data. To assess how the genes of this subsystem impact the transcription of ADH2, we performed a dynamic analysis modeling using ordinary differential equation (ODE).

To set up the equations and parameters for modeling, a model simplification was required due to the lack of information in the literature concerning many rates. First, every protein (node) was considered active because of the lack of information concerning phosphorylation and dephosphorylation for some proteins. Hence, the equations reflect the likelihood of a protein to enhance or inhibit the synthesis of its target. Second, to avoid bias in the model, values of protein synthesis and degradation, and protein complex formation were standardized to ~0.1 min^{-1} and ~0.5 min^{-1}, respectively. We assumed the basal and induced/enhanced synthesis as lower and higher than the degradation rate, respectively. These parameters were defined according to a quantitative model of *S. cerevisiae* (Kraikivski et al. 2015). Third, the inhibition rate was set up as a degradation rate. Then, the model was solved by using COPASI (Hoops et al. 2006) stating the initial molecule values = 0 or 1. The 0 was assigned for genes that increased the expression along the time-course (e.g., the lncRNA, IXR1, HSF1 and PRM4), whereas the 1 was assigned for genes that decreased the expression. The model was iteratively updated each second maintaining the proportionality aforementioned. All equations and parameters are presented in the **Supplementary Table 16**, and **Supplementary Table 17**.

6. Mutants generation

Full deletion mutants BMA64-1A transcr_20548Δ, BMA64-1A IXR1Δ, BMA64-1A CTA1Δ, and SEY6210 transcr_3536Δ, and the partial deletion mutants BY4742 transcr_10027Δ (from -80 to 1636) were generated using CRISPR-Cas9: the plasmids pMEL16 (Addgene 107922) (expressing the gRNA, carrying the His selection marker) and p414-TEF1p-Cas9-CYC1t (Addgene 43802, hereafter referred to as P414) (expressing the Cas9) were used. The P414 was modified to have a KAN selective marker rather than TRP1 (donated by Dr. Arnold Driessen of University of Groningen, The Netherlands).

Oligos were ordered to modify pMEL16 according to each target gene. Furthermore, 120 nts F and R repair oligos were ordered to allow the full knock-out of selected genes by homologous directed repair (HDR) strategy (**Supplementary Table 19**). To insert the genomic target region into pMEL16, we performed PCR using 20 ng pMEL16 plasmids, 1 µL of Phusion (NEB M0530S), 1X of Phusion Buffer, 0.1 mM of each dNTP, 0.4 µM of pMEL16 oliB oligo, and 0.4 µM of F oligo for a final volume of 25 µL. The touch-down reaction was set up as: 98°C for 1 min, 5 cycles of 98°C for 30 sec, X°C for 30 sec, 72°C for 6 min, 10 cycles of 98°C for 30 sec, Y°C for 30 sec, 72°C for 6 min, 20 cycles of 98°C for 30 sec, Z°C for 30 sec, 72°C for 6 min, and finally 72°C for 6 min, and 4°C constant time. The X°C, Y°C and Z°C annealing temperatures are the average between the melting temperature of pMEL16 oliB and the F oligo of each gene, plus 9°C, 5°C and 2°C, respectively. The original bacterial plasmids were digested by DpnI (NEB R0176S) using 25 µL of PCR product, 1X CutSmart buffer, 1 µL of DpnI, followed by an incubation at 37°C for 30 min. The digestion was purified using the Wizard SV Gel and PCR Clean-Up System (Promega A9281). A total of 100-200 ng of purified products was ligated by T4 DNA ligase (Promega M1801), and 2 µL of ligated plasmids were inserted into 40 µL of TOPO competent cells (incubation on ice for 20 min, heating at 42°C for 50 min, and heat shock for 2 min on ice). After, 200 µL of LB medium were added, and incubated at 37°C for 1h to recover the cell wall. Cells were transferred to LB plates with 0.05 mg/mL of ampicillin and incubated overnight. Colony PCR was performed to seek modified pMEL16 using M13 oligos (**Supplementary Table 19**), followed by incubation at 37°C for 1 h with ClaI enzyme (Bsu16I, Thermo Fisher IVGN0306). Positive colonies show a single band of 778 bp for the modified pMEL16, negative colonies have two bands (341 bp and 437 bp). Colonies with modified pMEL16 were grown overnight in LB with 0.05 mg/mL of ampicillin, followed by the plasmid extraction using the QuickLyse Miniprep system (Qiagem 27405).

Yeast cells grown overnight were diluted in YPD to an OD_{600} of 0.3 followed by incubation at 30°C and 200 RPM until OD_{600} of 1.0. Competent cells were obtained using the Yeast Transformation Kit (Sigma YEAST-1KT).

A solution with 10 µL of salmon testes DNA, 600 µL of plate buffer, 1 µg of P414 plasmid, 1 µg of modified and purified pMEL16, and 5 µL of double strand repair DNA were gently mixed with 100 µL of competent cells. The tubes were incubated at 30°C for 30 min, and 10% of DMSO was added and gently mixed. The tubes were immediately incubated at 42°C for 15 min, quickly transferred into ice to chill for 2 min. The salmon testes DNA and plate buffer came from the Yeast Transformation Kit (Sigma YEAST-1KT). The double

strand repair DNA was obtained mixing into a single tube 50 µM of F and R repair DNA oligos (see **Supplementary Table 19**) of each target gene. The repair DNA mixture was incubated at 95°C for 10 min followed by a slow cooling on the bench until it reached the room temperature.

Cells were harvested by centrifugation at 2,000 RPM for 30 sec and supernatant was discarded. The pellet was gently diluted in 250 µL of 1.92 mg/mL of drop-out medium His^- (Yeast Synthetic Drop-out Medium Supplement without Histidine, Sigma Y1751) supplemented with 2% of glucose, and 1.9 mg/mL of Yeast Nitrogen Base without Amino Acids and Ammonium Sulphate. The tubes were incubated at 30°C, and 200 RPM for 2 h to recover the cell wall. A total of 10 µL of cells were plated onto YPD medium (the positive control) and the rest of content was plated onto drop-out medium His^- (the same one already mentioned) added with 2% of bacto agar and 0.2 mg/mL of G418. Plates were incubated at 30°C for two or three days.

Mutant colonies were screened by standard colony PCR. Picked colonies were diluted in 50 µL of sterile water and PCR was performed using 1 µL of diluted cells and the oligos flanking the gene under evaluation (**Supplementary Table 19**); the amplicon size was checked in 2% of agarose gel electrophoresis. Putative mutants were sequenced by the Sanger method using the same set of primers mentioned. Mutants were grown overnight in liquid YPD with 0.2 mg/mL of G418, and 700 µL of cells with 15% of glycerol were stored at -80°C.

The ability of mutants and wild-type (BMA64-1A) to rebound after an extremely high EtOH stress was assessed as described in the 7th experiment reported in the topic **3.1. Growth curves**.

The spot test assay was performed to assess the highest EtOH level for each mutant. In this case, late log-phase cells were diluted to an OD_{600} of 0.4 and centrifuged at 2,000 RPM for 2 min and the supernatant was discarded. Then, YPD medium with different EtOH concentrations were used to dissolve the pellet and cells were transferred into 50 mL tubes in a final volume of 2mL. The tubes were incubated at 30°C, 200 RPM for 1h. Then, 10 µL of content was diluted in 90 µL of sterile water (1:10), and from this 4 additional 1:10 serial dilutions were performed; the content of each tube was mixed before being transferred to the next dilution. Finally, 5 µL of each tube was spotted in YPD plates and incubated at 30°C until colonies arose.

Results

1. Defining phenotypes, and cell growth analysis

The lack of colonies on plates after the highest EtOH concentration tested and the slopes of growth under the most severe condition that still allowed a growth showed the severeness of EtOH concentration here used (**Supplementary Figure 1C**).

The analysis of spermidine supplementation in YPD with EtOH is presented in the **Supplementary Figure 1D-F**.

2. Genome and lncRNA assembly, and annotation update

BMA64-1A genome sequencing had ~6,410,152 filtered reads ~250 nts in length were used to achieve 196 assemblies. The best assembly has 11,880,801 bp distributed onto 683 scaffolds, with 134.88 X of genomic coverage, and lacking only 36 coding genes compared to the S288C. The complete view of genome assembling is present in the **Supplementary Data 10 and Supplementary Table 2**.

The pipeline using Trinity followed by a re-assembling using CAP3 generated the most feasible results to assemble the lncRNAs: although the assembling score of Velvet/Oases slightly outperforms that of Trinity (**Supplementary Table 20**; **Supplementary Data 10**), the later was chosen because of its highest computational performance.

Most lncRNAs have small size ranging from ~200-400 nts (**Supplementary Figure 7A**). The RT-PCR and the Sanger sequencing of one of the longest lncRNA found (transcr_28768 of S288C, with 2,739 nts) proven the one is not an algorithm artifact (**Supplementary Figure 7D**): this lncRNAs was chosen because its size and prediction to bind to bulky proteins. The identified lncRNAs are near or within coding and intergenic regions usually in the antisense orientation (such as expected (Yamashita et al. 2016)) (**Supplementary Table 7**).

A small fraction of filtered lncRNAs present small ORFs starting with methionine. However, peptide sequences from small lncRNA-ORFs starting with methionine did not match peptides from our proteomics and did not have similarities to protein sequences or domains.

The lncRNA transcr_22854, and transcr_24032 of S288C have orthologues in five and four strains, respectively. Only 7 lncRNAs of S288C have an orthologue with a similarity ≥90% (**Supplementary Figure 7B-C**).

The TE annotation showed a diversity of TY elements including LTR/Copia, LTR/Gypsy, and LTR/Retrotransposon. These families are evenly distributed among strains, although BMA64-1A shows the highest number of copies (**Supplementary Table 21**). All strains present signs of TE mobilization along their evolution, mainly the LTR/Retrotransposons. All strains presented a recent insertion wave of each repeat family and another smaller, older wave. The older wave is more prominent in S288C (LTR/Retrotransposon). Despite X2180-1A and SEY6210 strains presenting more peaks of insertion, their TE percentages in the genome are similar to other strains (**Supplementary Figure 21**).

Comparing the SGD tRNA annotation for S288C SGD against tRNA searching here performed, we found a total of 255 tRNAs perfectly annotated by the tRNAscan-SE, being only the coordinated chrIV 1150842..1150941 and the chrXII 784354..784453 tRNAs not recovered by tRNAscan-SE. Taken together, only 48 tRNAs were discrepantly annotated when comparing the S288C SGD and tRNAscan-SE annotation.

3. Quantitative analysis of transcriptome, proteome, metabolome and networks

3.1. Differential expression: transcriptome

The gene expression analysis was performed using updated annotation, which harbor lncRNAs, TEs, and tRNAs as well (**Supplementary Data 1**). The PCA analysis showed differences in variances between control and treatment (**Supplementary Figure 22**).

Approximately 50.31% and 47.76% of the DEGs were upregulated in HTs and LTs, respectively (**Supplementary Table 4**). The up and downregulated gene ratio variation was normal (HTs had W = 0.9939 and p-value = 0.8507, while LTs had W = 0.99914, and p-value = 0.9441), and significantly differences considering HTs and LTs strains (t = 3.3179, p-value = 0.0442). Altogether, the data indicates that HTs tended to have more upregulated genes than LTs.

Interestingly, lncRNAs tended to be upregulated in EtOH stressed cells: the expression of lncRNAs were presented in the **Supplementary Figure 6A**, and **Supplementary Table 8**. The qPCR of some lncRNAs had a fit with RNA-Seq data (**Supplementary Figure 6B**).

Most TEs were upregulated in both phenotypes under stress. Although LTs presented significantly higher TE expression than HTs, the latter had a higher percentage of

upregulated TEs (87.66% vs. 61.51%) (**Supplementary Figure 23; Supplementary Data 14**).

The number of significant differentially expressed coding genes of HTs and LTs is depicted in **Supplementary Figure 3A-B**). Both phenotypes were not clearly clustered by expression (see heatmaps) (**Supplementary Figure 3C**).

3.2. Differential abundances: Proteomics

The differential abundant proteins (DAPs) found by proteomics were described in the **Supplementary Table 14**, and **Supplementary Figure 4**.

3.3. Differential abundances: Metabolomics

The PCA and clustering showed divergences in abundance between controls and treatment in all strains, as expected (**Supplementary Figure 24A-C**). The number of differentially abundant metabolites (DAM) per strain range from 101 (SEY6210) to 182 (BY4742) metabolites, without any bias to up or down DAM comparing all samples (**Supplementary Data 2**).

A total of 232 non-redundant DAMs were identified considering both phenotypes (**Supplementary Data 2, 9; Supplementary Figure 5A**). We considered a DAM as present in both phenotypes if it is present in at least one HT and one LT (**Supplementary Data 4**). A total of 16 and 9 DAMs are present only in HTs and LTs, respectively (**Supplementary Figure 5A**). Interestingly, the citrate cycle metabolism (a pathway also ranked as important according to transcriptome GO enrichment analysis) presents DAMs up-abundant in HTs and LTs (**Supplementary Data 4**).

3.4. Topological metrics of integrated networks

The 16 integrated networks (6 strains plus 2 phenotypes for control and treatment conditions) fit a power-law degree distribution (**Supplementary Figure 9A**), a scale-free network, and adjusted to the Barabási-Albert model. Topological properties analyzed suggested that these networks fits reliable biological networks (Newman 2002; Junker and Schreiber 2008; Takahashi et al. 2012; Erciyes 2015).

The networks are disassortative indicating the hubs are surrounded by low-degree nodes making the networks resilient to random perturbations. Despite the diameter being slightly higher (ranging from $d_m \approx 5$ to $d_m \approx 7$) than the average path length, the later

suggested adjustments to the small-world model effect. Overall, Diameter, path-length, and betweenness increased in most of the emulated treated networks, whereas density, transitivity, number of connections and eigenvector decreased (**Supplementary Table 10-11**).

The emulated HT and LT treated networks lost highly connected hubs and increasing the number of intermediary hubs; this effect was more pronounced in emulated LT networks. Finally, the normalized degree-degree correlation showed that stress increased the probability of connections among nodes with a lower degree (**Supplementary Figure 9; Supplementary Figure 25**).

Interestingly, the premature termination in the degree (*K*) distribution tail in LT network under treatment compared to the treated HT evidenced a pronounced loss of hubs in LT (**Supplementary Figure 9B**). According to the normalized DDC function analysis, the treatment networks also have higher probabilities of connection among nodes with lower degree (**Supplementary Figure 25**).

4. Qualitative analysis (functional description) of transcriptome, proteome, metabolome and networks

4.1. DEGs previously reported as responsive to other stressors

We selected the DEGs which are up or down regulated within phenotypes: genes up-regulated in almost all strains, except in one LT, were, we defined as up-regulated in HTs, and vice-versa. Many genes responsive to many fermentation stressors (Auesukaree 2017) were DEGs in our data. For instance, we found HTs' up-regulated DEGs only as: 1- genes responsive to the oxidative stress such as the SOD family genes (e.g., CTA1), glutathione peroxidases (e.g., GPX1), and the GSH1; 2- heat shock proteins (HPSs) that recover and degrade proteins, also matching our proteomics; 3- genes TPS1/2 (produce trehalose, which is important to maintain the protein stability), and the SS3/4 (HSP70 family). The only HTs' down-regulated are related to the osmotic stress response that activate the mitogen-activated protein kinase (MAPK) pathways (e.g., PBS2, MSB2, SSK22, GPD2, and STE20) (**Supplementary Table 15**).

4.2. Putative function of lncRNAs and structural analysis

The filtered LNCPI networks (interaction probability ≥95%) (**5.6.2. Integrating lncRNAs into networks, statistical analysis of networks, and network dynamic modeling**) harbor most of lncRNAs identified (**Supplementary Table 9**).

The LNCPIs are reliable, fitting the number of edges (ncRNA-protein interactions) for yeast (Panni et al. 2017) and the number of target proteins (Chujo et al. 2016). Functions of lncRNAs assigned by guilt-by-association strategy are described in the **Supplementary Figure 8; Supplementary Data 5**. The enrichment of the term "RNA polymerase assembling" suggests that lncRNA target-proteins are working on transcription by RNA polymerases (RNAP). Analyzing the expression of genes related to RNAP II (88 proteins), I (10 proteins) and III (5 proteins) showed that most of the genes related to RNAPII are up-regulated in almost all strains (BMA64-1A is the exception). In fact, 78 target genes are DE (half up-regulated) in more than 3 strains and only 10 genes are up-regulated only in half of strains (**Supplementary Figure 8A**). Although the enriched term "negative regulation of ncRNA transcription associated with protein coding genes TSS/TES" (**Supplementary Figure 8A**) suggests the presence of a negative regulation of lncRNAs adjacent to *cis* coding genes, we did not observe expression differences of these lncRNAs comparing treatment *vs* control for all strains, neither comparing the time-course of S288C and BMA64-1A.

The lncRNAs which target the same proteins within S288C and within BMA64-1A have low similarities (from 50.87% to 63.76%) and also a lack of secondary structure conservation; the only exception is transcr_22263 vs trancr_20495 of BMA64-1A (the ones target the YLR105C) with a structural distance score (SDS) of 0.055. Conversely, the inter-strain comparisons (comparisons between secondary structure of orthologs) showed a secondary structure conservation between most orthologs.

The four categories of functional process of EtOH-responsive lncRNAs found by the lncRNA-propagation analysis from each DE lncRNA is depicted in the **Supplementary Figure 26A**.

Gln3p is bound to transcr_6448 in BMA64-1A, depicted in the down-regulated sub-network. Gln3p is a transcriptional activator of genes subject to nitrogen catabolite repression (Courchesne and Magasanik 1988; Beck and Hall 1999). Despite there is a strong negative correlation under treatment condition between the expression of transcr_6448 and GLN3, the GLN3 expression slightly increases under treatment along the time-course, while the transcr_6448 expression abruptly decreases followed by a slight

increases (**Supplementary Figure 26C**). Taking into account the lncRNA-protein network selected for S288C and its time-course data, we sough whether the time-course expression profile of metabolism related genes were related to that of lncRNA transcr_18666. We observed a strong negative correlation between transcr_18666 and ATH1 in EtOH-stressed S288C, with an expression strong expression of ATH1 gene, suggesting a high demanding of Ath1p (**Supplementary Figure 26B**). Ath1p degrades trehalose (Jules et al. 2004).

Finally, the information of transcriptome, and metabolome per strain were integrated KEGG maps; lncRNAs (**Supplementary Data 5**) were manually integrated on these maps. In almost all cases the lncRNA expression is inversely related to its target-protein (**Supplementary Figure 16**.

4.3. Narrowing down the pathways affected by the EtOH stress

4.3.1. Overview of GO enrichment analysis

The **Supplementary Table 5** depicts the GO enriched terms using g:Profiler (Reimand et al. 2016) enriched within HTs, with LTs and shared between both phenotypes. HT and LTs shared many differentially expressed genes and functions, mainly in which upregulated genes were related to metabolism, whereas most downregulated were related to RNA processing.

The enrichment analysis of KEGG pathways using GAGE was performed for all strains being the BY4741 the exception (it did not have enrichment with FDR <0.01) (**Supplementary Table 6**).

4.3.2. General metabolic pathways affected by the EtOH

Alcohol metabolism: ACS1 (YAL054C) is up-regulated in almost all strains. ACC1 (YNR016C) is down-regulated in almost all strains, and only BY4742 does not have one lncRNA bound to Acc1p (**Supplementary Data 1; Supplementary Figure 16**).

TCA cycle, and related pathways (ethanol buffering model): the gene GDH3 (YAL062W), ALD4 (YOR374W), ETR1 (YBR026C), YAT1 (YAR035W), YAT2 (YER024W), CAT2 (YML042W), HFD1 (YMR110C), and peroxisomal genes PEX1 (YKL197C), PEX3 (YDR329C), PEX6 (YNL329C), PXA1 (YPL147W), PXA2 (YKL188C), and FAA2 (YER015W) are up-regulated in all or almost all strains. Interestingly, Yat2p interacts with lncRNAs in BY4742, and SEY6210 (**Supplementary Data 1; Supplementary Data 5**). The metabolite oxaloacetate (also present in 8 other pathways) is up-abundant in most of LTs and down-abundant in most of HTs (**Supplementary Data 4, 10, and 12**). The metabolite

fumarate is down-abundant only in HTs (except the X2180-1A) (**Supplementary Data 4 and 10; Supplementary Figure 16**).

Fatty acid/lipid metabolism: the gene YDC1 (YPL087W), YPC1 (YBR183W), PBN1 (YCL052C), and FAA1 (YER015W) are up-regulated in all or almost all strains. The genes ERG1 (YGR175C, down-regulated in almost all strains) and ERG9 (YHR190W, up and down-regulated in two HTs and two LTs, respectively). The gene SFA1 (YDL168W) is down-regulated only in LTs. The gene EKI1 (YDR147W), and AUR1 (YKL004W) are down-regulated in all strains (**Supplementary Data 1; Supplementary Figure 16B**). Sphingosine is down-abundant in all strains, while the metabolite sphinganine is up-abundant only in HTs. Squalene is up-abundant in HTs and down-abundant in LTs (**Supplementary Data 4, 8, 10 and 12; Supplementary Figure 16**).

Terpenoid backbone biosynthesis: COQ1 (YBR003W) and HMG2 (YLR450W) were up-regulated in all strains, while ERG8 (YMR220W) is down-regulated. STE24 (YJR117W) is up-regulated only in HTs (**Supplementary Data 1 and 12**).

Nitrogen/alanine/arginine metabolism: the threonine and serotonin are up-abundant in at least two LTs and down-abundant in two HTs (**Supplementary Data 4 and 10**).

4.3.3. Basal pathways affected by EtOH

The basal pathways affected by EtOH include the cell cycle, TCA cycle, glycolysis and gluconeogenesis, autophagy, MAPK, longevity, protein process in endoplasmic reticulum (PPER), RNA transport, ribosome biogenesis, mRNA surveillance, and RNA degradation. Most of DEGs of these pathways are down-regulated being longevity, PPER, autophagy, TCA cycle and glycolysis and gluconeogenesis the exceptions (**Supplementary Data 6**. We highlight that most of up-regulated genes on these KEGG pathways are related to degradation systems, such as MPD1 (YOR288C), SHP1 (YBL058W), YET3 (YDL072C), ERAD components, HSP26/42 (YBR072W/YDR171W), HSP82 (YPL240C), SSA3/4 (YBL075C/YER103W), FES1 (YBR101C), SSB2 (YNL209W), non-sense mediated decay genes (DCS1 (YLR270W), and DCS2 (YOR173W)), mRNA surveillance (TPD3 (YAL016W)), chaperones, and heat shock proteins (HSPs) (the HSPs are also up-abundant in our proteomes) (see protein processing in endoplasmic reticulum, RNA degradation, and mRNA surveillance pathways, and proteomes). We also highlight the importance of genes ROS-related and antioxidant systems such as CTA1 (YDR256C), CTT1 (YGR088W), and SOD1 (YJR104C), which are up-regulated in almost all strains, the peroxisomal protein PEX12, which is down-regulated only in LTs, and the lack of downregulation for most of

genes related to longevity (e.g., GPR1, RAS2, CYR1, TPK1, RIM15, GIS1, and SOD2) (**Supplementary Data 1 and 12**).

The pathway integrated network (PINET) (**5.6.1. Modeling the KEGG pathway-based networks based on time-course data**) was structured in four communities: 1) MAPK and cell cycle; 2) TCA and Gly/gluc; 3) longevity, autophagy, and PPER; 4) mRNA surveillance, RNA degradation, RNA biogenesis and transport, and ribosome biogenesis (**Supplementary Figure 10A**). The clustering analyses using the time-course data of treatment condition for both BMA64-1A and S288C showed that EtOH induced intense rewiring in the 12 essential pathways analyzed in both phenotypes (**Supplementary Figure 10B**).

Each essential pathway had one out of 3 expression landscapes: 1) “up and stable” when most of genes increase the expression from 1h to 2h and maintain the expression until 4h; 2) “down and stable” when most of genes decrease expression followed by the maintenance of expression; 3) “stable” when most of genes does not change the expression along the time-course. The expression profile using the time-course data of all genes linked to each node-pathway without a steady-state landscape were compared for BMA64-1A and S288C networks (HT and LT models, respectively) (**Supplementary Figure 11A**), reveling the presence of “up and stable”, “stable” and “down and stable” (**Supplementary Figure 11B-D, and G**).

The comparisons between the expression landscapes (**Supplementary Figure 11B-D**) *vs.* network propagation (this network is not phenotype-specific) from each node-pathway is reported in **Supplementary Figure 11E-F and Supplementary Figure 12**. The general profile of expression along the time for all pathways analyzed and for some selected genes is presented in the (**Supplementary Figure 11G-H**). The population rebound of BMA64-1A CTA1Δ after extreme severe EtOH stresses are higher than the BMA64-1A wild-type (from 35% to 40% of EtOH), whereas in 42.5% the wild-type outperforms the mutant; however only the differences at 37.5% of EtOH was statistically significant (**Supplementary Figure 11I**).

4.3.4. Diauxic shift mechanism mediates the ethanol buffering

We modeled an EtOH buffering mechanism based on the diauxic shift pathway. Most genes in this model are up-regulated and impact the metabolite abundances. We highlight that two HTs and two LTs were properly clustered based on the expression of genes from this model (**Supplementary Figure 27**). We highlight the upregulation of the following genes

essential for growth on nonfermentable carbon sources and diauxic shift-responsive genes: (NQM1 (YGR043C), CAT8 (YMR280C), ADR1 (YDR216W), INO4 (YOL108C), GUT1/2 (YHL032C/YIL155C), and RSF1 (YMR030W)) (Dasgupta et al. 2002; Klein et al. 2017), genes essential to metabolize the acetyl-CoA in the cytosol and peroxisomes (ALD4 (YOR374W), FAA1 (YOR317W), FAA2 (YER015W), HFD1 (YMR110C), ACS1 (YAL054C), PXA1 (YPL147W), PXA2 (YKL188C), POX1 (YGL205W), FOX2 (YKR009C), and POT1 (YIL160C)), carnitine acetyltransferases (CAT2 (YML042W), YAT1 (YAR035W), YAT2 (YER024W)), and TCA genes (CIT2 (YCR005C), GDH3 (YAL062W), GDH2 (YDL215C), KGD1 (YIL125W), and KGD2 (YDR148C)).

There are different expression profiles, patterns and edges comparing the BMA64-1A and S288C subnetworks, mainly concerning ADH2 (**Supplementary Figure 28B-C**). Some genes of BMA64-1A subnetwork share similar transcriptional profiles, but different profiles in S288C (**Supplementary Figure 28A-C**).

4.3.5. Acidification assay, and membraneless structures affected by the EtOH stress

Only the highest and mid EtOH stress prompt the steady-state on pH and growth in BMA64-1A and S288C. Overall, the lowest EtOH concentrations reach the higher growth rates and consequently the higher external acidification. There is a negative correlation (Pearson two-tailed) between pH and *K* parameter within each EtOH level and strain for all EtOH stress levels (**Supplementary Table 12**).

The membraneless structures and pathways extremely affected by the EtOH stress is reported in the **Supplementary Data 6, and 15**. LncRNAs bind to proteins of these structures and pathways (**Supplementary Figure 26)**. Overall, few genes are down-regulated for most of pathways, BMA64-1A presents the highest percentage of up-regulated and not DE genes, PBs-related genes were usually down-regulated, and HTs have significant lesser down-regulated genes related to RNA catabolic process than LTs (**Supplementary Figure 14A**).

We highlight that essential PB and SG genes (*e.g.*, CDC33/eIF4E, eIF4A/TIF1, DCP1, DCP2, PAB1, PUB1/TIA1, SUP45, and HBS1), mRNA decay and surveillance (*e.g.*, LSM1, DCP1, DCP2, RRP40, RRP43, RRP45, RRP46, RRP41/SKI6, EDC3, NUC1, CAF40, and MPP6), ribosome biogenesis and transport (*e.g.*, CKA2, CKB2, RRP7, UTP4, UTP8, GAR1, EMG1, and NOP4, among others) (Eulalio et al. 2007; Standart and Weil 2018; Falcone and Mazzoni 2018; van Leeuwen and Rabouille 2019) were not downregulated in BMA64-1A, unlike other strains (**Supplementary Data 1**).

The lncRNAs in LNCPI (see **5.6.2. Integrating lncRNAs into networks, statistical analysis of networks, and network dynamic modeling**) bind proteins of membraneless structures and pathways (**Supplementary Data 5; Supplementary Figure 14D**). Comparing the number of lncRNAs observed *vs* expected per structure, only PBs, proteasome, and PDNR deviates the number of lncRNAs from expected; PBs have lesser lncRNAs than expected, unlike proteasome and PDNR (**Supplementary Figure 14B-C; Supplementary Data 9**).

The percentage of lncRNAs connecting synergistic pathways (within "storage" or "degradation") was positively related to the population rebound after stress relief and negatively related to the highest EtOH levels analyzed (**Supplementary Data 9; Supplementary Figure 14E**). The EtOH tolerance reduced in BY4742 transcr_10027Δ and the lethality of SEY6210 transcr_3536Δ observed (**Supplementary Figure 14F-H**).

5. Cell biology analysis

5.1. Cell viability, ROS, SDH, DNA damage assays, RNA yield, glucose influx, and western blot

The cell viability was evaluated to determine the cellular surveillance of each strain under the severe EtOH stress (**Supplementary Data 8; Supplementary Figure 2A-D**). The chi-square independence test comparing treatment *vs* control considering all LL, LR, UL and UR quadrants, showed significant differences in cell viability for almost all strains (**Supplementary Table 3**).

The differences of SDH activity comparing treatment *vs* control are significant for almost all HTs and not significant for LTs (**Supplementary Table 3**). Although both phenotypes under treatment had an increase of SDH activity, the one is more active in HTs (see rate T/C in **Supplementary Table 13**).

HTs under treatment presented a significant higher fraction of cells with ROS (**Supplementary Table 13; Supplementary Data 8; Supplementary Figure 2E-F**).

Both phenotypes under treatment increased the number of cells with DNA damage, although LTs presented a higher fraction of cells with damage (**Supplementary Table 13; Supplementary Data 8**).

Analysis of time-course expression profile of genes related to mismatch, base, and nucleotide excision repair pathways (KEGG sce03440, sce03410, and sce03420,

respectively) supports the DNA damage measurements: it is observed a higher down-regulation of these pathway in LTs (**Supplementary Figure 29**).

There are two mechanisms that simultaneously control the DNA damage checkpoint leading and maintaining the cell cycle arrest until repair, the Rad53p and Chk1p branches. The RAD53 (YPL153C) is down-regulated in most strains (**Supplementary Data 1**), hence, the ones might have the RAD53-arrest inactivated. Additionally, only LTs presented a normal expression of PDS1 (YDR113C), indicating cell cycle arrest by PDS1-blocking. Finally, only X2180-1A and BY4742 have both mechanisms collapsed (**Supplementary Figure 15**).

The nuclear export gene LOS1 (YKL205W), and GSP2 (YOR185C) were down and up-regulated in most strains. Moreover, RNA synthesis and degradation (**Supplementary Data 6; Supplementary Figure 10**) are pathways affected by the severe EtOH stress. Then, to assess whether the severe EtOH stress is impacting the RNA yield, we performed acridine orange staining. Only the X2180-1A increased the RNA yield in the whole cells under treatment (no statistic significant). Conversely, under treatment, only the BMA64-1A RNA yield is significantly reduced in the whole cell, and for the cytosol and nucleus yield in X2180-1A, according to the acridine orange assay (**Supplementary Figure 13**).

Concerning the western blot, comparing both strains, we observed a higher abundance of these three proteins in BMA64-1A than S288C under EtOH stress, albeit only the Pab1p difference is statistically significant (p-value 0.031).

Discussion

The most relevant pathways affected by EtOH stress were selected based on the DEGs, DAMs, DAPs, GO analysis, KEGG mapping, and lncRNA functional assignments. Our findings were divided into four sections. The most affected pathways are autophagy (KEGG sce04138), cell cycle (KEGG sce04111), citrate cycle (TCA cycle) (KEGG sce00020), fatty acid metabolism (KEGG sce01212), glutathione metabolism (KEGG sce00480), glycerophospholipids (KEGG sce00564), glycine/serine/threonine metabolism (KEGG sce00260), glycolysis/gluconeogenesis pathway (KEGG sce00010), glycosylphosphatidylinositol (GPI)-anchor biosynthesis (KEGG sce00563), longevity (KEGG sce04213), MAPK signaling pathway (KEGG 04011), mRNA surveillance (KEGG sce03015), nitrogen/alanine/arginine metabolisms (KEGG sce00220, sce00250, and sce00910), peroxisome (KEGG sce04146), proteasome (KEGG sce03050), protein

processing in endoplasmic reticulum (KEGG sce04141), ribosome and ribosome biogenesis (KEGG sce03010, and sce03008), RNA degradation (KEGG sce03018), RNA transport (KEGG sce03013), sphingolipid (KEGG sce00600), and terpenoid backbone biosynthesis (KEGG sce00900).

Considering the full set of analysis, focus on the mentioned pathways, our discussion was separated into five sections.

The first section reports EtOH stress-responsive lncRNAs. The key points are: 1- lncRNAs act on systems in a strain-specific manner to overcome EtOH stress; 2- EtOH stress-responsive lncRNAs can be categorized as "life essential", "membrane-dependent", "metabolic", or "degradation" processes; 3- in stressed cells, the Nrp1p migration into SG inhibits trapping transcr_20548 of BMA64-1A, allowing this lncRNA to repress ADH2.

The second section covers the systems signaling analysis from EtOH stress throughout basal systems. The key points are: 1- under EtOH stress, cells favor to maintain basal pathways active in order to preserve and prepare themselves for stress relief; 2- system signals are primarily transmitted from MAPK to longevity, peroxisomes, and autophagy pathways; 3- the first EtOH stress-responsive systems to exhibit phenotype-specific expression profiles are CTA1, longevity, and peroxisomes; 4- the accumulation of CTA1 and ROS may be essential to initiate the EtOH stress phenotype-specific profile; 5- under EtOH stress, spermidine is in balance with the SGF29 gene; 6- inhibition of sphingolipids, ceramides, and inositol-phosphorylceramide pathways may be additional hurdles for cell surveillance during EtOH stress; 7- SUI2 seems to bridge the network systems signaling from autophagy to RNA and ribosome biological pathways; 8- the TCA cycle is quickly reached by the systems signal, activating this systems in both phenotypes.

The third section reports the role of membraneless organelles, storage and degradation pathways in EtOH tolerance. The key points are: 1- EtOH stress creates a suitable environment for assembling of membraneless structures; 2- under EtOH stress, cells use degradation systems such as membraneless organelles to withstand the harm caused by DNA damage, and HT seems to take advantage of these processes.

The fourth section, we scrutinize our EtOH buffering model. The key point is that the diauxic shift drives an EtOH buffering mechanism, promoting an energy burst to hinder damage from EtOH stress; HTs seem to take advantage of this process.

1. EtOH stress responsive lncRNAs are functionally diverse and likely involved in EtOH tolerance

We highlight that the action of EtOH stress-responsive lncRNAs were conjected based on how their target-proteins act on cells. Moreover, we intended to present hypotheses concerning the action of lncRNAs in EtOH tolerance of yeast to basis further studies rather than present decisive and robust roles.

First, the lncRNA-protein networks revealed that many lncRNAs found here are related to transcription regulation and alcohol catabolic process. Second, the yeast lncRNAs can be classified into two groups: 1- transcriptional regulators that act through histone modification, which is subdivided into transcriptional interference (block transcription of adjacent mRNAs), gene looping (activate the expression of adjacent genes), *cis* repressor, and *trans* repressor; 2- non-transcriptional functionals lncRNAs, which is subdivides into RNP scaffold (*e.g.*, telomerase) and telomere regulators (Niederer et al. 2017; Till et al. 2018). The guilt-by-association and systems signaling throughout LNCPI (the lncRNA propagation analysis) supported the lncRNA functions. Despite strain specificities, we could classify lncRNAs into broader categories. We suggest that EtOH stress-responsive lncRNAs act as bait, backbones, or adapters in a strain-specific manner to overcome stress challenges.

The general gene upregulation of lncRNA target proteins related to "transcription by RNA polymerase II", suggested that EtOH stress may induce the expression of RNAP-related genes. Interestingly, mutated Rbp7p (RNAPII subunit) enhanced EtOH tolerance, affecting the glycolysis, fermentation, and oxidative stress response genes (Qiu and Jiang 2017), pathways also prevalent in our analyses. We hypothesize that lncRNA-RNAPII interactions might be acting as signaling complexes to counterbalance the transcription and degradation rate of mRNAs, which could explain the preferential mRNA down-regulation observed in LTs (see **Supplementary Table 4**); indeed, lncRNAs can contribute to a balance of transcription and degradation rate (Timmers and Tora 2018). Additionally, most RNA catabolism process-related genes are not dampened in all strains here analysed, and fundamental genes of RNA degradation (*e.g.*, DCS1, DCS2, and DCP2, according to Parker (Parker 2012)), were not down-regulated.

Strain-specificities concerning lncRNA functions was recurrent here. Functional diversity of lncRNA was observed for the plant *Gossypium hirsutum*. LncRNAs responding to salt stress in this species *cis* or *trans* regulate genes of a wide range of biological

processes evidencing that lncRNAs work on more than one stress-related mechanism (Deng et al. 2018).

The down-regulated lncRNA transcr_6448 of BMA64-1A had one target-protein coded by GLN3 gene (**Supplementary Figure 26A**). The time-course data showed that transcr_6448 of BMA64-1A might also help to induce its highest EtOH tolerance. This lncRNA seems to repress GLN3 under EtOH stress through a negative feedback loop. GLN3 deletion boosts branched-chain alcohol tolerance or may lead to a delay in cell cycle progression (Kuroda et al. 2019). We hypothesize that the GLN3 regulators GCN4 and YAP6 may work synergistically in this process since all of these genes are downregulated only in BMA64-1A. The finding mentioned fits our hypothesis since BMA64-1A is the only strain with the down- regulation of GLN3 and presents the highest EtOH tolerance here analyzed.

The down-regulated lncRNA transcr_63478 of BY4742 work on signaling, cell wall, intracellular transport, transcription, replication and ribosome biogenesis (**Supplementary Figure 26A**). The lncRNA mentioned targets the Cin8p (YEL061C), which is responsible for the mitotic spindle assembly and chromosome segregation (Roof et al. 1992). The lack of CIN8 leads to a delay in the cell cycle progression (Straight et al. 1998; Mittal et al. 2020). Remarkably, CIN8 is down-regulated in all HT strains, whereas it is not DE in most of LTs, indicating this gene may be related to the low rebound after stress relief in HTs. Additionally, this lncRNA also binds to Def1p (YKL054C), a RNAPII degradation factor (further discussed) (Woudstra et al. 2002).

It is known that EtOH affects the yeast's cell wall components (Aguilar-Uscanga and Francois 2003). The down-regulated lncRNAs transcr_8290 of X2180-1A work on membranes and cell wall, and transcription. In this case, we showed that lncRNA seems to act as a scaffolder of protein complexes related to membrane and cell wall, binding to the ATPases Dnf1p (YER166W), Neo1p (YIL048W), Dsr2p (YAL026C), Dnf3 (YMR162C), chitin synthases Chs3p (YBR023C), Chs1p (YNL192W), and to the membrane protein Pdr5p (YOR153W). The type 4 P-type ATPases are responsible to transport phospholipids through a bilayer membrane (Paulusma and Oude Elferink 2005), chitin synthases are essential to the synthesis of the chitin for the cell wall (Ziman et al. 1996), and membrane protein responsible to multi-drug transport act as a drug responsive system (Golin et al. 2007). Interestingly, the expression of PDR5 (YOR153W) decreases during diauxic growth or when nutrients are depleted (Mamnun et al. 2004). Interestingly, we observed that PDR5 is down-regulated in all strains, which seems to be a response of glucose depletion observed,

reinforcing our diauxic-shift model as an ethanol buffering mechanism (further discussed) (**Supplementary Figure 26A**).

LncRNAs down-regulated of S288C seems to act as scaffolder of protein complexes responsible for ribosomal biogenesis (**Supplementary Figure 26A**). The lncRNAs transcr_18820, transcr_21244, and transcr_6225 bind to Ecm16p (YMR128W), and Rea1p/Mdn1p (YLR106C), which are small nucleolar ribonucleoprotein (snoRNP), and ribosome biogenesis proteins, respectively (Shiratori et al. 1999; Colley et al. 2000; Nissan 2002). Additionally, transcr_19266 interacts with Rpa190p (YOR341W), which is part of RNA Polymerase I (RNAPI) (Kuhn et al. 2007). Despite our transcriptome and proteome showing that ribosomal biogenesis, and ribosomal substrates (*e.g.*, tRNAs and elongation factor) were deeply negatively affected in almost all strains analyzed, we could not infer any positive or negative role of these lncRNAs on ribosomal biogenesis of S288C under the severe EtOH stress.

The up-regulated lncRNAs of BY4741 work on "membrane dependent process" (**Supplementary Figure 26A**). The flippases Drs2p (YAL026C), and Dnf2p (YDR093W) bind to transcr_3338 and transcr_2916, respectively. These flipases are P-type ATPases that concentrate phosphatidylserine and phosphatidylethanolamine on the cytosolic leaflet, contributing to endocytosis, intracellular transport, and cell polarity (Chen et al. 1999; Hua et al. 2002; Pomorski et al. 2003; Iwamoto et al. 2004). Transcr_2916 also binds to the two low-affinity phosphate transporters Pho90p (YJL198W) and Pho87p (YCR037C); the overexpression of these genes lead to an abnormal cell cycle progression and a reduction of vegetative growth rate (Stevenson et al. 2001; Sopko et al. 2006; Yoshikawa et al. 2011). BY4741 is the only strain with up-regulation of these PHOs genes and, remarkably, this strain has the slower rebound among LTs after the severe EtOH stress. Furthermore, the ubiquitin-specific proteasome (further discussed) Ubp13p (YBL067C) (Amerik et al. 2000) binds to the transcr_1287. All genes mentioned are up-regulated only in BY4741, indicating a possible positive impact of lncRNAs on these genes and on the general process directly dependent on the membrane's physiology.

The up-regulated lncRNAs of SEY6210 seem to act mainly on "membrane dependent processess" in the cell modeling processes (such as the ones during the cell cycle). In this case, the interaction between transcr_8157 and transcr_9136 with proteins Bni1p (YNL271C), Sla1p (YBL007C), and Hbt1p (YDL223C), indicates these lncRNAs may work on cell cortex modeling system, albeit the mentioned genes have different roles (**Supplementary Figure 26A**). Sla1p is a cytoskeletal binding protein associated with

endocytosis, or binds to proteins to regulate actin dynamics (Pruyne and Bretscher 2000; Howard et al. 2002). Bni1p and Hbt1p are responsible for cell polarization (Lee et al. 1999; Dittmar 2002; Pruyne et al. 2002, 2004; Tcheperegine et al. 2005; Guarente 2010). Although BNI1 over-expression could have deleterious effects (Evangelista et al. 1997), the up-regulation observed in SEY6210 (the only strain with this gene up-regulated) does not seem to harm cells. Additionally, the proteins Yat2p (YER024W), and Azf1p (YOR113W), which are related to mitochondria and diauxic shift, respectively (the importance of this system and organelle will be further discussed) (Swiegers et al. 2001; Zampar et al. 2013) interacts with transcr_9136.

The up-regulated transcr_18666 of S288C binds to Gpb1p, which is responsible to promote ubiquitin-dependent proteolysis (the essentiality of degradation processes will be further discussed). Furthermore, this lncRNA also seems to work on work on trehalose metabolism. Our metabolome showed an unexpected trehalose accumulation in S288C (**Supplementary Data 8**) since the time-course data showed that ATH1 was increasing under stress. Ath1p degrades trehalose (Jules et al. 2004), while trehalose accumulation induces cell viability (Mansure et al. 1994). Trehalose is abundant in LTs (**Supplementary Data 4**), and ATH1 was highly expressed in S288C when transcr_18666 decreased (**Supplementary Figure 26B-C; Supplementary Data 1**). The Ath1p interacts with transcr_18666 (**Supplementary Figure 26A**). Interestingly, S288C has the lowest EtOH tolerance. Altogether, our data indicates that the lncRNA transcr_18666 of S288C may be dampening the Ath1p activity resulting in the observed trehalose accumulation. Interestingly, trehalose accumulation induces cell viability (Mansure et al. 1994), as observed in S288C.

The up-regulated lncRNAs of X2180-1A may be contributing to the cellular surveillance mediated by “degradation process” and “membrane dependent process”. The interaction between transcr_3746, and transcr_6988 with Ubr2p (YLR024C), indicates the earlier work on protein degradation. Ubr2p is an ubiquitin-protein ligase required for the ubiquitination and degradation of Rpn4p (YDL020C) (Ju et al. 2008), exerting a negative control over 26S proteasome (Owsianik et al. 2002) (the essentiality of proteasome in EtOH will be further discussed). Moreover, Tao3p (YIL129C) (related to cell morphogenesis, and proliferation (Du and Novick 2002)) and Cyr1p (YJL005W) (works on signal transduction which is required for cAMP production (Kataoka et al. 1985)) bind to transcr_3746. The cAMP is involved in cell cycle progression, sporulation, cell growth, stress response and longevity (Casperson et al. 1985). Hence, transcr_3746 may be also related to cell longevity, growth, and proliferation (**Supplementary Figure 26A**).

The up-regulated lncRNAs of BY4742 work on cell signaling, cell division and degradation (**Supplementary Figure 26A**). Transcr_10883, transcr_10027 and transcr_9158 binds to the Pik1p (YNL267W), a kinase able to rapidly restore the nutrients supply in cells under nutrient deprivation (Demmel et al. 2008). Pik1p works on the phosphatidylinositol signaling system (KEGG sce04070) by converting the phosphatidylinositol to phosphatidylinositol 4-phosphate (Garcia-Bustos et al. 1994), a pathway that we suggest to act on the EtOH tolerance (see topic **5.**). Thus, since Inositol 1-phosphate (which also come from phosphatidylinositol) is up abundant in LT strains (**Supplementary Data 4**), the lncRNAs may play a role dampening the Inositol 1-phosphate synthesis in BY4742 (a HT phenotype).

Additionally, the up-regulated lncRNAs transcr_10883, transcr_10027, and transcr_9158 of BY4742 also seem to work on cell surveillance along the stationary-phase here observed, signaling, homeostasis, and oxidative stress response (as a response of ROS accumulation observed), by a conjoint action with PBs (**Supplementary Figure 26A**). Indeed, stationary-phase, cytosol acidification, and cell wall, oxidative and EtOH stress induces/enhances PBs formation (Buchan et al. 2008; Kato et al. 2011; Ramachandran et al. 2011; Shah et al. 2013; Wang et al. 2018; van Leeuwen and Rabouille 2019; García et al. 2019). PBs are required for surveillance along the stationary phase in yeast (Ramachandran et al. 2011), as well as signaling molecules hijacking, albeit it is not clear if they have effects on signal transduction (Zhang and Herman 2020). These lncRNAs binds to Ecm5p, Rim15p, Tel1p, Bph1p, Mtc5p, among other. Mtc5p is related to cell signaling (Panchaud et al. 2013), and Rim15p is involved in signal transduction to enter into the stationary phase (Reinders et al. 1998). Bph1p is related to cell wall formation and response to pH (Rieger et al. 1997; Shiflett et al. 2004), and Ecm5p works on oxidative stress response (Baker et al. 2013). Tel1p represses PBs formation (Tkach et al. 2012), suggesting that transcr_10027 is related to the cell cycle re-entry after stress relief. Altogether, these data, the increasing of cells accumulating ROS under the EtOH treatment, and the stationary-phase observed along the severe EtOH stress, evidences that transcr_10883, transcr_10027 and transcr_9158 might be working on PBs for a long-term survival during stationary-phase.

The role of lncRNA over RNAPII regulation was also modeled here. The lncRNA transcr_20548 of BMA64-1A works on the "positive regulation of transcription from RNA polymerase II promoter in response to EtOH", a child GO term of "negative regulation of

protein kinase activity by protein phosphorylation"; the later was also enriched by guilt-by-association analysis.

2. EtOH causes extensive rewiring of life-essential pathways: longevity, peroxisome, and CTA1 are master key regulators of EtOH tolerance phenotypes

The data from BMA64-1A strain suggest that the positive effect of spermidine on growth relies on a certain level of SGF29 expression and likely some level of H3 lysine acetylation. The spermidine increases EtOH production and tolerance to some fermentation inhibitors (Kim and Auh 2021). The role of spermidine relies on SGF29 protein scarcity/inactivation: Sgf29p acetylates the H3 lysine (Bian et al. 2011). Spermidine hypoacetylates the same residue, increasing the lifespan (Eisenberg et al. 2009). In fact, spermidine overload increased the growth of all strains during long-term EtOH exposure, except for BMA64-1A, the only strain presenting downregulation of SGF29. Therefore, the data from strain BMA64-1A suggest that the positive effect of spermidine on growth relies on a certain level of SGF29 expression and likely some level of H3 lysine acetylation. Finally, this report is the first report to document the action of spermidine in EtOH tolerance.

The systems underwent intensive rewiring under the severe EtOH stress modifying the topological features (**Supplementary Table 10; Supplementary Table 11; Supplementary Figure 9; Supplementary Figure 25; Supplementary Figure 10; Supplementary Figure 11**).

The activation of only basal systems by delaying to late response pathways via creating longer path is evidenced by the increased diameter, path length, and betweenness, and reductions in density and transitivity in emulated treated networks. Indeed, the low transitivity indicates that the hub neighborhoods are sparsely connected (Mason and Verwoerd 2007), suggesting longer paths. Longer paths favor network modularity (Xu et al. 2011), leading to a signal delay from the regulators to the regulatory responses (Klein et al. 2012). Moreover, systems under stress require that only basal modules remain active, a dynamism represented by the betweenness (Doncheva et al. 2012). We showed that EtOH stress reduced the alternative routes, which was demonstrated by the decrease in the degree, eigenvector, and connection of highly connected hubs (increasing the connections of mid connected hubs). These features indicate that less important nodes lose connections with more relevant nodes (Lohmann et al. 2010). The increase in BC and the decrease in density (such as observed here) indicated a reduction in alternative route availability (Burt

2004; Buskens and van de Rijt 2008). Therefore, we suggest that longer paths, system delays, and tightening of alternative routes prompt (or reflect) lower flexibility in the active pathways under EtOH stress.

To better understand the modification mentioned, we evaluated subsystems. Again, EtOH stress leads to extensive rewiring, even in the MAPK, longevity, autophagy, TCA, RNA-related pathways, RNA and protein degradation systems, Gly/gluc, ribosome biogenesis, cell cycle, and protein process in endoplasmic reticulum (see **Supplementary Figure 10B**).

Although peroxisome is associated to ROS accumulation, early aged cells increased the number of peroxisomes but reduced the ROS yield, likely catabolized by CTA1 and CTT1 catalases (Deb et al. 2022). CTA1 plays a role in EtOH tolerance (Du and Takagi 2007) and lifespan (Weinberger et al. 2010), also evidenced by analyzing the BMA64-1A CTA1Δ. Therefore, the early ROS accumulation of EtOH-stressed cells observed here (1h of stress) is unexpected because we observe an intense expression of CTA1 in the same period.

Additionally, the expression of SCH9, CTT1, and PEX12 evidenced that ROS accumulation observed is an early EtOH stress response. The SCH9 downregulation in all HTs may be related to their expected higher oxidative stress resistance since the ones presented high ROS accumulation: the lack of SCH9 increases oxidative stress resistance (Teixeira et al. 2014). Such as here observed, oxidative stress and carbon starvation trigger the expression of the longevity-related gene CTT1, which cope with oxidative stress (Herrero et al. 2008; Auesukaree 2017). Peroxisome biogenesis (a relevant source of ROS) requires PEX12 expression (Chang et al. 1997; Auesukaree 2017), a gene also required for EtOH resistance (Teixeira et al. 2009). Therefore, we suggest that the reduction in PEX12 observed in LTs may be related to their low EtOH tolerance and ROS accumulation.

The complex called glucose induced degradation deficiency (GID) produces glucose from non-carbohydrate carbon during glycolysis. During stationary phase with glucose depletion, peroxisomes may influence the gluconeogenesis by binding two GID's proteins in the presence of non-fermentation carbon such as EtOH (Yifrach et al. 2022). Time-course expression profiles of peroxisome and Gly/Glu are similar in both BMA64-1A and S288C, and glucose level is reduced in most analyzed strains. Therefore, we speculate that extreme EtOH stress may triggers the GID related-mechanism mentioned independent of EtOH stress-phenotype.

Altogether, here we had shown that longevity, peroxisomes, CTA1, and SUI2 are master key regulators of EtOH tolerance.

3. Membraneless organelles, storage, and degradation systems are related to EtOH stress: lncRNAs also act on these systems

The Dcp1p and Pab1p are present in PBs and SGs, respectively (Eulalio et al. 2007; van Leeuwen and Rabouille 2019). 5'-3' mRNA decay relies on decapping mainly by the Dcp1p and Dcp2p (Parker 2012). Although the eIF4E binds to the 5'-cap and Pab1p to enhance translation initiation (Dever et al. 2016), the decapping and decay are independent of the Pab1p presence (Parker 2012).

PSGs are proteasome reservoirs that protect cells from stress by preventing the degradation of other cytosolic proteins during stress, promoting a delay in the reentry into the proliferative state, and genotoxic resistance (Peters et al. 2013; Saunier et al. 2013).

Ufd4p (YKL010C) is an ubiquitin-protein ligase related to 26S proteasome (Hochstrasser 1996), which interacts to transcr_7869 (up-regulated) of BY4742 (**Supplementary Figure 26A**). A mechanism of lncRNA ubiquitin inhibition was observed in hepatocellular carcinoma cells (Ni et al. 2017), then, we suggest that transcr_7869 may be acting as a ubiquitin-bait blocking the ubiquitin ligase avoiding the protein degradation; it could contribute to protein post-stress recovery, which can be faster than re-starting the whole translation mechanism, contributing to EtOH tolerance in this strain. Interestingly, UFD4 is up-regulating only in BY4742, reinforcing the putative influence of transcr_7869 on this gene.

Finally, we suggest that the cell cycle reentry observed is SG-dependent and mediated by the upregulation of HSP104 (YLL026W) and SSE2 (YBR169C). These genes encode proteins to degrade yeast SGs, allowing the reactivation of proteins and arrested RNAs, inducing cell cycle reentry (Kroschwald et al. 2015).

4. The EtOH stress-buffering model

Below is the citation and descriptions of genes and metabolites related to EtOH-buffering model (**Table 3** in the main text).

- ADR1, CAT8, GUT1, GUT2, INO4, NQM1 and RSF1: (Dasgupta et al. 2002; Klein et al. 2017).

- ALD4, ACS1, FAA1, FAA2, FOX2, HFD1, PXA1, PXA2, POX1, and POT1: (Black 1951; Shani and Valle 1996; Hiltunen et al. 2003; Nakahara et al. 2012; Chen et al. 2012; de Jong et al. 2014); SGD database;
- CIT2, GDH3, GDH2, KGD1, KGD2, and PDC1: SGD database; KEGG sce00010; (Xiao et al. 2022);
- ADH3, ADH5, and SFA1: KEGG sce00010; YeastPathway EC Number 1.1.1.1; (Xiao et al. 2022);
- LSC1, LSC2, FUM1, MLS1, MDH1, MDH2, GDH3, GDH2, KGD1, KGD2, MDH3, PCK1, PYC1, PDA1, LAT1, oxaloacetate, fumarate, and malate: (Repetto and Tzagoloff 1989; Miller and Magasanik 1990; DeLuna et al. 2001; Reinders et al. 2007; Martínez-Reyes and Chandel 2020; SGD database 2021);
- ETR1: (Miinalainen et al. 2003) (KEGG sce00062).

The genes involved in the conversion from acetaldehyde or aldehyde to acetyl-CoA in cytosol, and peroxisomes are up-regulated in most strains. Ald4p converts acetaldehyde to acetate (Black 1951), and Hfd1p synthesizes fatty acids from aldehydes (Nakahara et al. 2012). The catabolism of acetaldehyde to acetyl-CoA by Acs1p (de Jong et al. 2014) may be occurring in the cytosol; this enzyme is located in cytosol, mitochondria, peroxisome, and nucleus (Chen et al. 2012). Indeed, ACS complex avoids EtOH damage in the yeasts (de Jong et al. 2014). The fatty acids are delivered into peroxisome by Pxa1p, and Pxa2p (Shani and Valle 1996), and once, a complex pathway involving Pox1p, Fox2p, and Pot1p produce acetyl-CoA (Hiltunen et al. 2003).

Cat2p converts the acetyl-CoA in acetyl-carnitine in peroxisomes (van Roermund et al. 1999), further transported to the mitochondria by CATs Yat1p and Yat2p (Schmalix and Bandlow 1993). YAT2 and CIT2 double mutant does not grow on nonfermentable carbon source media (Swiegers et al. 2001).

Oxaloacetate can take different pathways within mitochondria. First, it can be converted to phosphoenol-pyruvate by Pck1p and then to pyruvate. Then, pyruvate can be converted back to oxaloacetate by Pyc1p; or the pyruvate can be converted by Pdb1p and Pda1p to 2-hydroxyethyl-ThPP and s-aceltyldihydrolipoamido-E. The latter one can be transformed in acetyl-CoA again by Lat1p. Gdh3p synthesizes glutamate from alpha-ketoglutarate (2-oxoglutarate) while Gdh2p makes the reversible reactions (DeLuna et al. 2001). The last reaction mentioned synthesizes NADH (Miller and Magasanik 1990), such as the further catabolism of alpha-ketoglutarate in succinyl-CoA by Kgd1p and Kgd2p

(Repetto and Tzagoloff 1989; Reinders et al. 2007), which is an essential molecule to pump H^+ by the mitochondrial respiratory chain. The fumarate is further converted to malate (Martínez-Reyes and Chandel 2020). Finally, the null GDH3 mutant reduced the vegetative growth under EtOH stress, and this gene is induced under EtOH stress (DeLuna et al. 2001; SGD database 2021) (**Supplementary Table 13**).

5. Other interesting genes and mechanisms likely affected by EtOH stress

EtOH promptly affects the plasma membrane of yeast deregulating cellular homeostasis (Kane 2016). Lipidomics in EtOH-stressed yeasts revealed differences in lipid saturation rather than composition (Lairón-Peris et al. 2021). The metabolic maps integrating our transcriptome and metabolome data suggest that lipid metabolites may be related to the negative effect of EtOH on cell surveillance or longevity. Previous findings revealed that the inhibition of sphingolipids, ceramides, and IPC synthesis and the lack of the AUR1 gene negatively affect cell division, growth, lifespan and/or viability (Wu et al. 1995; Giaever et al. 2002; Epstein et al. 2012; Katsuki et al. 2018). The overexpression of ERG9 (synthesizes squalene) reduces yeast growth (Yoshikawa et al. 2011), and the accumulation of squalene also reduces growth and cell viability (Yoshikawa et al. 2011; Garaiová et al. 2014; Valachovic et al. 2016; Csáky et al. 2020). EtOH-stressed cells of both phenotypes analyzed here displayed a reduced sphingosine yield and expression of many sphingolipid, ceramide and inositol-phosphorylceramide (IPC) synthesis-related genes (mainly AUR1), which might be an additional hurdle for cell surveillance during severe EtOH stress. The higher cell death rate observed in HT strains than in LT strains is likely related to their exposure to a higher EtOH level. However, only HT strains accumulate ERG9, squalene and sphinganine, which might be responsible for exacerbating the negative effects on cell viability in this phenotype.

Stress can activate TEs expression (*e.g.*, Long Terminal Repeat (LTR) retrotransposons), inducing mutations but helping the organism adaptation (McClintock 1984). For instance, in yeast, the transcription of the LTR Ty1 is induced by a shortage of adenylic nucleotides (González et al. 2010). Interestingly, yeasts after EtOH pretreatment can acquire better EtOH tolerance (Lewis et al. 2010). The EtOH stress here evaluated was severe for both phenotypes inducing TEs expression. Altogether, this TE induction may be related to the higher DNA damage observed under stress, and also indicate that strains may be endeavoring to adapt to this stress. It rejects the hypothesis that fast rebound

counterbalances the high cell death rate after severe EtOH stress musing a high EtOH tolerance phenotype.

Terpenoid backbone biosynthesis: COQ1 (YBR003W) (null mutant reduce the EtOH resistance) and HMG2 (YLR450W) were up-regulated in all strains, while ERG8 (YMR220W) is down-regulated (null mutant cause abnormal mitochondrial morphology). The STE24 (YJR117W) is up-regulated only in HTs (increase the competitive fitness under EtOH) (**Supplementary Data 1 and 12**; SGD phenotype descriptions).

Serotonin stimulates the yeast growth (Malikina et al. 2010), and threonine is related to the EtOH stress tolerance (Takagi et al. 2005). Since both metabolites are down in HTs and up-abundant in LTs (**Supplementary Data 10**), we associate the serotonin to the higher population rebound in LTs, which is enhanced by the EtOH tolerance from threonine boosting caused by this stress.

Supplementary References

Aguilar-Uscanga B, Francois JM (2003) A study of the yeast cell wall composition and structure in response to growth conditions and mode of cultivation. Lett Appl Microbiol 37:268–274. https://doi.org/10.1046/j.1472-765X.2003.01394.x

Almeida LF de, Moraes LN de, Santos LD dos, Valente GT (2019) Development and comparative analysis of yeast protein extraction protocols for mass spectrometry. Anal Biochem 567:90–95. https://doi.org/10.1016/j.ab.2018.10.028

Amerik AY, Li SJ, Hochstrasser M (2000) Analysis of the deubiquitinating enzymes of the yeast Saccharomyces cerevisiae. Biol Chem 381:981–92. https://doi.org/10.1515/BC.2000.121

Arrial RT, Togawa RC, Brigido M de M (2009) Screening non-coding RNAs in transcriptomes from neglected species using PORTRAIT: case study of the pathogenic fungus Paracoccidioides brasiliensis. BMC Bioinformatics 10:239. https://doi.org/10.1186/1471-2105-10-239

Auesukaree C (2017) Molecular mechanisms of the yeast adaptive response and tolerance to stresses encountered during ethanol fermentation. J Biosci Bioeng 124:133–142. https://doi.org/10.1016/j.jbiosc.2017.03.009

Baker LA, Ueberheide BM, Dewell S, et al (2013) The yeast Snt2 protein coordinates the transcriptional response to hydrogen peroxide-mediated oxidative stress. Mol Cell Biol 33:3735–48. https://doi.org/10.1128/MCB.00025-13

Bankevich A, Nurk S, Antipov D, et al (2012) SPAdes: A New Genome Assembly Algorithm and Its Applications to Single-Cell Sequencing. J Comput Biol 19:455–477. https://doi.org/10.1089/cmb.2012.0021

Beck T, Hall MN (1999) The TOR signalling pathway controls nuclear localization of nutrient-regulated transcription factors. Nature 402:689–692. https://doi.org/10.1038/45287

Berretta J, Pinskaya M, Morillon A (2008) A cryptic unstable transcript mediates transcriptional trans -silencing of the Ty1 retrotransposon in S. cerevisiae. Genes Dev 22:615–626. https://doi.org/10.1101/gad.458008

Bian C, Xu C, Ruan J, et al (2011) Sgf29 binds histone H3K4me2/3 and is required for SAGA complex recruitment and histone H3 acetylation. EMBO J 30:2829–42. https://doi.org/10.1038/emboj.2011.193

Black S (1951) Yeast aldehyde dehydrogenase. Arch Biochem Biophys 34:86–97. https://doi.org/10.1016/S0003-9861(51)80013-4

Blohm P, Frishman G, Smialowski P, et al (2014) Negatome 2.0: a database of non-interacting proteins derived by literature mining, manual annotation and protein structure analysis. Nucleic Acids Res 42:D396–D400. https://doi.org/10.1093/nar/gkt1079

Bolger AM, Lohse M, Usadel B (2014) Trimmomatic: a flexible trimmer for Illumina sequence data. Bioinformatics 30:2114–20. https://doi.org/10.1093/bioinformatics/btu170

Bradford M (1976) A Rapid and Sensitive Method for the Quantitation of Microgram Quantities of Protein Utilizing the Principle of Protein-Dye Binding. Anal Biochem 72:248–254. https://doi.org/10.1006/abio.1976.9999

Buchan JR, Muhlrad D, Parker R (2008) P bodies promote stress granule assembly in Saccharomyces cerevisiae. J Cell Biol 183:441–455. https://doi.org/10.1083/jcb.200807043

Bumgarner SL, Dowell RD, Grisafi P, et al (2009) Toggle involving cis -interfering noncoding RNAs controls variegated gene expression in yeast. Proc Natl Acad Sci 106:18321–18326. https://doi.org/10.1073/pnas.0909641106

Burge SW, Daub J, Eberhardt R, et al (2013) Rfam 11.0: 10 years of RNA families. Nucleic Acids Res 41:D226-32. https://doi.org/10.1093/nar/gks1005

Burt RS (2004) Structural Holes and Good Ideas. Am J Sociol 110:349–399. https://doi.org/10.1086/421787

Buskens V, van de Rijt A (2008) Dynamics of Networks if Everyone Strives for Structural Holes. Am J Sociol 114:371–407. https://doi.org/10.1086/590674

Camblong J, Beyrouthy N, Guffanti E, et al (2009) Trans -acting antisense RNAs mediate transcriptional gene cosuppression in S. cerevisiae. Genes Dev 23:1534–1545. https://doi.org/10.1101/gad.522509

Cantarel BL, Korf I, Robb SMC, et al (2008) MAKER: An easy-to-use annotation pipeline designed for emerging model organism genomes. Genome Res 18:188–196. https://doi.org/10.1101/gr.6743907

Carlin DE, Demchak B, Pratt D, et al (2017) Network propagation in the cytoscape cyberinfrastructure. PLoS Comput Biol 13:1–9. https://doi.org/10.1371/journal.pcbi.1005598

Carr M, Bensasson D, Bergman CM (2012) Evolutionary Genomics of Transposable Elements in Saccharomyces cerevisiae. PLoS One

7:e50978. https://doi.org/10.1371/journal.pone.0050978
Casperson GF, Walker N, Bourne HR (1985) Isolation of the gene encoding adenylate cyclase in Saccharomyces cerevisiae. Proc Natl Acad Sci 82:5060–5063. https://doi.org/10.1073/pnas.82.15.5060
Chang CC, Lee WH, Moser H, et al (1997) Isolation of the human PEX12 gene, mutated in group 3 of the peroxisome biogenesis disorders. Nat Genet 15:385–8. https://doi.org/10.1038/ng0497-385
Chatr-Aryamontri A, Breitkreutz BJ, Heinicke S, et al (2013) The BioGRID interaction database: 2013 Update. Nucleic Acids Res 41:470–478. https://doi.org/10.1093/nar/gks1158
Chen CY, Ingram MF, Rosal PH, Graham TR (1999) Role for Drs2p, a P-type ATPase and potential aminophospholipid translocase, in yeast late Golgi function. J Cell Biol 147:1223–36. https://doi.org/10.1083/jcb.147.6.1223
Chen Y, Siewers V, Nielsen J (2012) Profiling of Cytosolic and Peroxisomal Acetyl-CoA Metabolism in Saccharomyces cerevisiae. PLoS One 7:e42475. https://doi.org/10.1371/journal.pone.0042475
Chong J, Xia J (2018) MetaboAnalystR: an R package for flexible and reproducible analysis of metabolomics data. Bioinformatics 34:4313–4314. https://doi.org/10.1093/bioinformatics/bty528
Chujo T, Yamazaki T, Hirose T (2016) Architectural RNAs (arcRNAs): A class of long noncoding RNAs that function as the scaffold of nuclear bodies. Biochim Biophys Acta - Gene Regul Mech 1859:139–146. https://doi.org/10.1016/j.bbagrm.2015.05.007
Colley A, Beggs JD, Tollervey D, Lafontaine DLJ (2000) Dhr1p, a Putative DEAH-Box RNA Helicase, Is Associated with the Box C+D snoRNP U3. Mol Cell Biol 20:7238–7246. https://doi.org/10.1128/mcb.20.19.7238-7246.2000
Courchesne WE, Magasanik B (1988) Regulation of nitrogen assimilation in Saccharomyces cerevisiae: roles of the URE2 and GLN3 genes. J Bacteriol 170:708–713. https://doi.org/10.1128/JB.170.2.708-713.1988
Cowen L, Ideker T, Raphael BJ, Sharan R (2017) Network propagation: A universal amplifier of genetic associations. Nat Rev Genet 18:551–562. https://doi.org/10.1038/nrg.2017.38
Csáky Z, Garaiová M, Kodedová M, et al (2020) Squalene lipotoxicity in a lipid droplet-less yeast mutant is linked to plasma membrane dysfunction. Yeast 37:45–62. https://doi.org/10.1002/yea.3454
Csardi G, Nepusz T (2006) The igraph software package for complex network research. InterJournal Complex Sy:1695
Cuadros-Inostroza Á, Caldana C, Redestig H, et al (2009) TargetSearch - a Bioconductor package for the efficient preprocessing of GC-MS metabolite profiling data. BMC Bioinformatics 10:428. https://doi.org/10.1186/1471-2105-10-428
Dasgupta A, Darst RP, Martin KJ, et al (2002) Mot1 activates and represses transcription by direct, ATPase-dependent mechanisms. Proc Natl Acad Sci U S A 99:2666–71. https://doi.org/10.1073/pnas.052397899
de Jong BW, Shi S, Siewers V, Nielsen J (2014) Improved production of fatty acid ethyl esters in Saccharomyces cerevisiae through up-regulation of the ethanol degradation pathway and expression of the heterologous phosphoketolase pathway. Microb Cell Fact 13:39. https://doi.org/10.1186/1475-2859-13-39
Deb R, Ghose S, Nagotu S (2022) Increased peroxisome proliferation is associated with early yeast replicative ageing. Curr Genet 68:207–225. https://doi.org/10.1007/s00294-022-01233-3
Delli Ponti R, Armaos A, Marti S, Tartaglia GG (2018) A Method for RNA Structure Prediction Shows Evidence for Structure in lncRNAs. Front Mol Biosci 5:. https://doi.org/10.3389/fmolb.2018.00111
DeLuna A, Avendano A, Riego L, Gonzalez A (2001) NADP-glutamate dehydrogenase isoenzymes of Saccharomyces cerevisiae. Purification, kinetic properties, and physiological roles. J Biol Chem 276:43775–83. https://doi.org/10.1074/jbc.M107986200
Demmel L, Beck M, Klose C, et al (2008) Nucleocytoplasmic Shuttling of the Golgi Phosphatidylinositol 4-Kinase Pik1 Is Regulated by 14-3-3 Proteins and Coordinates Golgi Function with Cell Growth. Mol Biol Cell 19:1046–1061. https://doi.org/10.1091/mbc.e07-02-0134
Deng F, Zhang X, Wang W, et al (2018) Identification of Gossypium hirsutum long non-coding RNAs (lncRNAs) under salt stress. BMC Plant Biol 18:23. https://doi.org/10.1186/s12870-018-1238-0
Dever TE, Kinzy TG, Pavitt GD (2016) Mechanism and Regulation of Protein Synthesis in Saccharomyces cerevisiae. Genetics 203:65–107. https://doi.org/10.1534/genetics.115.186221
Dittmar GAG (2002) Role of a Ubiquitin-Like Modification in Polarized Morphogenesis. Science (80-) 295:2442–2446. https://doi.org/10.1126/science.1069989
Doncheva NT, Assenov Y, Domingues FS, Albrecht M (2012) Topological analysis and interactive visualization of biological networks and protein structures. Nat Protoc 7:670–685. https://doi.org/10.1038/nprot.2012.004
Du L-L, Novick P (2002) Pag1p, a Novel Protein Associated with Protein Kinase Cbk1p, Is Required for Cell Morphogenesis and Proliferation in Saccharomyces cerevisiae. Mol Biol Cell 13:503–514. https://doi.org/10.1091/mbc.01-07-0365
Du X, Takagi H (2007) N-Acetyltransferase Mpr1 confers ethanol tolerance on Saccharomyces cerevisiae by reducing reactive oxygen species. Appl Microbiol Biotechnol 75:1343–1351. https://doi.org/10.1007/s00253-007-0940-x
Eisenberg T, Knauer H, Schauer A, et al (2009) Induction of autophagy by spermidine promotes longevity. Nat Cell Biol 11:1305–1314. https://doi.org/10.1038/ncb1975
Epstein S, Castillon GA, Qin Y, Riezman H (2012) An essential function of sphingolipids in yeast cell division. Mol Microbiol 84:1018–1032. https://doi.org/10.1111/j.1365-2958.2012.08087.x
Erciyes K (2015) Analysis of Biological Networks. In: Distributed and Sequential Algorithms for Bioinformatics, 23rd edn. Springer, Cham, pp 213–240
Eulalio A, Behm-Ansmant I, Izaurralde E (2007) P bodies: at the crossroads of post-transcriptional pathways. Nat Rev Mol Cell Biol 8:9–22. https://doi.org/10.1038/nrm2080
Evangelista M, Blundell K, Longtine MS, et al (1997) Bni1p, a yeast formin linking cdc42p and the actin cytoskeleton during polarized morphogenesis. Science 276:118–22. https://doi.org/10.1126/science.276.5309.118
Fabregat A, Jupe S, Matthews L, et al (2014) The Reactome Pathway Knowledgebase. Nucleic Acids Res 44:D481–D487. https://doi.org/10.1093/nar/gkt1102
Falcone C, Mazzoni C (2018) RNA stability and metabolism in regulated cell death, aging and diseases. FEMS Yeast Res 18:. https://doi.org/10.1093/femsyr/foy050
Fernandes LP, Annibale A, Kleinjung J, et al (2010) Protein Networks Reveal Detection Bias and Species Consistency When Analysed by Information-Theoretic Methods. PLoS One 5:e12083. https://doi.org/10.1371/journal.pone.0012083
Finn RD, Bateman A, Clements J, et al (2014) Pfam: The protein families database. Nucleic Acids Res 42:222–230. https://doi.org/10.1093/nar/gkt1223
Garaiová M, Zambojová V, Šimová Z, et al (2014) Squalene epoxidase as a target for manipulation of squalene levels in the yeast Saccharomyces cerevisiae. FEMS Yeast Res 14:310–323. https://doi.org/10.1111/1567-1364.12107
Garcia-Bustos JF, Marini F, Stevenson I, et al (1994) PIK1, an essential phosphatidylinositol 4-kinase associated with the yeast nucleus. EMBO J 13:2352–61
García R, Pulido V, Orellana-Muñoz S, et al (2019) Signalling through the yeast MAPK Cell Wall Integrity pathway controls P-body assembly upon cell wall stress. Sci Rep 9:3186. https://doi.org/10.1038/s41598-019-40112-9
Geisler S, Lojek L, Khalil AM, et al (2012) Decapping of Long Noncoding RNAs Regulates Inducible Genes. Mol Cell 45:279–291.

https://doi.org/10.1016/J.MOLCEL.2011.11.025
Gelfand B, Mead J, Bruning A, et al (2011) Regulated Antisense Transcription Controls Expression of Cell-Type-Specific Genes in Yeast. Mol Cell Biol 31:1701–1709. https://doi.org/10.1128/MCB.01071-10
Giaever G, Chu AM, Ni L, et al (2002) Functional profiling of the Saccharomyces cerevisiae genome. Nature 418:387–91. https://doi.org/10.1038/nature00935
Goenawan IH, Bryan K, Lynn DJ (2016) DyNet: Visualization and analysis of dynamic molecular interaction networks. Bioinformatics 32:2713–2715. https://doi.org/10.1093/bioinformatics/btw187
Golin J, Ambudkar S V., May L (2007) The yeast Pdr5p multidrug transporter: How does it recognize so many substrates? Biochem Biophys Res Commun 356:1–5. https://doi.org/10.1016/j.bbrc.2007.02.011
González J, Karasov TL, Messer PW, Petrov DA (2010) Genome-wide patterns of adaptation to temperate environments associated with transposable elements in Drosophila. PLoS Genet 6:33–35. https://doi.org/10.1371/journal.pgen.1000905
Guarente L (2010) Forever young. Cell 140:176–8. https://doi.org/10.1016/j.cell.2010.01.015
Gullberg J, Jonsson P, Nordström A, et al (2004) Design of experiments: an efficient strategy to identify factors influencing extraction and derivatization of Arabidopsis thaliana samples in metabolomic studies with gas chromatography/mass spectrometry. Anal Biochem 331:283–295. https://doi.org/10.1016/j.ab.2004.04.037
Gurevich A, Saveliev V, Vyahhi N, Tesler G (2013) QUAST: quality assessment tool for genome assemblies. Bioinformatics 29:1072–1075. https://doi.org/10.1093/bioinformatics/btt086
Haas BJ, Papanicolaou A, Yassour M, et al (2013) De novo transcript sequence reconstruction from RNA-seq using the Trinity platform for reference generation and analysis. Nat Protoc 8:1494–1512. https://doi.org/10.1038/nprot.2013.084
Hall M, Frank E, Holmes G, et al (2009) The WEKA data mining software. Elsevier
He B, Zhao S, Chen Y, et al (2015) Optimal assembly strategies of transcriptome related to ploidies of eukaryotic organisms. BMC Genomics 16:1–10. https://doi.org/10.1186/s12864-014-1192-7
Herrero E, Ros J, Bellí G, Cabiscol E (2008) Redox control and oxidative stress in yeast cells. Biochim Biophys Acta - Gen Subj 1780:1217–1235. https://doi.org/10.1016/j.bbagen.2007.12.004
Hiltunen JK, Mursula AM, Rottensteiner H, et al (2003) The biochemistry of peroxisomal β-oxidation in the yeast Saccharomyces cerevisiae. FEMS Microbiol Rev 27:35–64. https://doi.org/10.1016/S0168-6445(03)00017-2
Hochstrasser M (1996) Ubiquitin-dependent protein degradation. Annu Rev Genet 30:405–39. https://doi.org/10.1146/annurev.genet.30.1.405
Hoffman DE, Jonsson P, Bylesjö M, et al (2010) Changes in diurnal patterns within the Populus transcriptome and metabolome in response to photoperiod variation. Plant Cell Environ 33:no-no. https://doi.org/10.1111/j.1365-3040.2010.02148.x
Hongay CF, Grisafi PL, Galitski T, Fink GR (2006) Antisense Transcription Controls Cell Fate in Saccharomyces cerevisiae. Cell 127:735–745. https://doi.org/10.1016/j.cell.2006.09.038
Hoops S, Sahle S, Gauges R, et al (2006) COPASI - A COmplex PAthway SImulator. Bioinformatics 22:3067–3074. https://doi.org/10.1093/bioinformatics/btl485
Houseley J, Rubbi L, Grunstein M, et al (2008) A ncRNA Modulates Histone Modification and mRNA Induction in the Yeast GAL Gene Cluster. Mol Cell 32:685–695. https://doi.org/10.1016/J.MOLCEL.2008.09.027
Howard JP, Hutton JL, Olson JM, Payne GS (2002) Sla1p serves as the targeting signal recognition factor for NPFX(1,2)D-mediated endocytosis. J Cell Biol 157:315–26. https://doi.org/10.1083/jcb.200110027
Hua Z, Fatheddin P, Graham TR (2002) An essential subfamily of Drs2p-related P-type ATPases is required for protein trafficking between Golgi complex and endosomal/vacuolar system. Mol Biol Cell 13:3162–77. https://doi.org/10.1091/mbc.e02-03-0172
Huang X (1999) CAP3: A DNA Sequence Assembly Program. Genome Res 9:868–877. https://doi.org/10.1101/gr.9.9.868
Huang Y-C, Chen H-T, Teng S-C (2010) Intragenic transcription of a noncoding RNA modulates expression of ASP3 in budding yeast. RNA 16:2085–2093. https://doi.org/10.1261/rna.2177410
Huber F, Bunina D, Gupta I, et al (2016) Protein Abundance Control by Non-coding Antisense Transcription. Cell Rep 15:2625–2636. https://doi.org/10.1016/j.celrep.2016.05.043
Iwamoto K, Kobayashi S, Fukuda R, et al (2004) Local exposure of phosphatidylethanolamine on the yeast plasma membrane is implicated in cell polarity. Genes Cells 9:891–903. https://doi.org/10.1111/j.1365-2443.2004.00782.x
Jackman SD, Vandervalk BP, Mohamadi H, et al (2017) ABySS 2.0: resource-efficient assembly of large genomes using a Bloom filter. Genome Res 27:768–777. https://doi.org/10.1101/gr.214346.116
Jones TR, Kang IH, Wheeler DB, et al (2008) CellProfiler Analyst: data exploration and analysis software for complex image-based screens. BMC Bioinformatics 9:482. https://doi.org/10.1186/1471-2105-9-482
Ju D, Wang X, Xu H, Xie Y (2008) Genome-Wide Analysis Identifies MYND-Domain Protein Mub1 as an Essential Factor for Rpn4 Ubiquitylation. Mol Cell Biol 28:1404–1412. https://doi.org/10.1128/MCB.01787-07
Jules M, Guillou V, François J, Parrou J-L (2004) Two distinct pathways for trehalose assimilation in the yeast Saccharomyces cerevisiae. Appl Environ Microbiol 70:2771–8. https://doi.org/10.1128/aem.70.5.2771-2778.2004
Junker BH, Schreiber F (2008) Analysis of Biological Networks. Wiley
Kane PM (2016) Proton Transport and pH Control in Fungi. In: Ramos J, Sychrová H, Kschischo M (eds) Yeast Membrane Transport. Advances in Experimental Medicine and Biology. Springer, Cham, pp 33–68
Kataoka T, Broek D, Wigler M (1985) DNA sequence and characterization of the S. cerevisiae gene encoding adenylate cyclase. Cell 43:493–505. https://doi.org/10.1016/0092-8674(85)90179-5
Kato K, Yamamoto Y, Izawa S (2011) Severe ethanol stress induces assembly of stress granules in Saccharomyces cerevisiae. Yeast 28:339–347. https://doi.org/10.1002/yea.1842
Katsuki Y, Yamaguchi Y, Tani M (2018) Overexpression of PDR16 confers resistance to complex sphingolipid biosynthesis inhibitor aureobasidin A in yeast Saccharomyces cerevisiae. FEMS Microbiol Lett 365:. https://doi.org/10.1093/femsle/fnx255
Kim D, Langmead B, Salzberg SL (2015) HISAT: a fast spliced aligner with low memory requirements. Nat Methods 12:357–360. https://doi.org/10.1038/nmeth.3317
Kim S-K, Auh J-H (2021) Evaluating the Engineered Saccharomyces cerevisiae With High Spermidine Contents for Increased Tolerance to Lactic, Succinic, and Malic Acids and Increased Xylose Fermentation. Biotechnol Bioprocess Eng 26:47–54. https://doi.org/10.1007/s12257-020-0020-y
Kim S, Lee DY, Wohlgemuth G, et al (2013) Evaluation and optimization of metabolome sample preparation methods for Saccharomyces cerevisiae. Anal Chem 85:2169–2176. https://doi.org/10.1021/ac302881e
Klein C, Marino A, Sagot M-F, et al (2012) Structural and dynamical analysis of biological networks. Brief Funct Genomics 11:420–433. https://doi.org/10.1093/bfgp/els030
Klein M, Swinnen S, Thevelein JM, Nevoigt E (2017) Glycerol metabolism and transport in yeast and fungi: established knowledge and ambiguities. Environ Microbiol 19:878–893. https://doi.org/10.1111/1462-2920.13617
Kong L, Zhang Y, Ye Z-Q, et al (2007) CPC: assess the protein-coding potential of transcripts using sequence features and support vector machine. Nucleic Acids Res 35:W345–W349. https://doi.org/10.1093/nar/gkm391

Kopka J, Schauer N, Krueger S, et al (2005) GMD@CSB.DB: the Golm Metabolome Database. Bioinformatics 21:1635–1638. https://doi.org/10.1093/bioinformatics/bti236
Kraikivski P, Chen KC, Laomettachit T, et al (2015) From START to FINISH: Computational analysis of cell cycle control in budding yeast. npj Syst Biol Appl 1:1–9. https://doi.org/10.1038/npjsba.2015.16
Kroschwald S, Maharana S, Mateju D, et al (2015) Promiscuous interactions and protein disaggregases determine the material state of stress-inducible RNP granules. Elife 4:. https://doi.org/10.7554/eLife.06807
Kuhn C-D, Geiger SR, Baumli S, et al (2007) Functional Architecture of RNA Polymerase I. Cell 131:1260–1272. https://doi.org/10.1016/j.cell.2007.10.051
Kuroda K, Hammer SK, Watanabe Y, et al (2019) Critical Roles of the Pentose Phosphate Pathway and GLN3 in Isobutanol-Specific Tolerance in Yeast. Cell Syst 9:534-547.e5. https://doi.org/10.1016/j.cels.2019.10.006
Kyriakou D, Stavrou E, Demosthenous P, et al (2016) Functional characterisation of long intergenic non-coding RNAs through genetic interaction profiling in Saccharomyces cerevisiae. BMC Biol 14:106. https://doi.org/10.1186/s12915-016-0325-7
Lairón-Peris M, Routledge SJ, Linney JA, et al (2021) Lipid Composition Analysis Reveals Mechanisms of Ethanol Tolerance in the Model Yeast Saccharomyces cerevisiae. Appl Environ Microbiol 87:. https://doi.org/10.1128/AEM.00440-21
Langmead B, Salzberg SL (2012) Fast gapped-read alignment with Bowtie 2. Nat Methods 9:357–9. https://doi.org/10.1038/nmeth.1923
Lee L, Klee SK, Evangelista M, et al (1999) Control of mitotic spindle position by the Saccharomyces cerevisiae formin Bni1p. J Cell Biol 144:947–61. https://doi.org/10.1083/jcb.144.5.947
Lewis JA, Elkon IM, McGee MA, et al (2010) Exploiting natural variation in Saccharomyces cerevisiae to identify genes for increased ethanol resistance. Genetics 186:1197–1205. https://doi.org/10.1534/genetics.110.121871
Li W, Godzik A (2006) Cd-hit: a fast program for clustering and comparing large sets of protein or nucleotide sequences. Bioinformatics 22:1658–9. https://doi.org/10.1093/bioinformatics/btl158
Licata L, Briganti L, Peluso D, et al (2012) MINT, the molecular interaction database: 2012 update. Nucleic Acids Res 40:D857–D861. https://doi.org/10.1093/nar/gkr930
Lohmann G, Margulies DS, Horstmann A, et al (2010) Eigenvector centrality mapping for analyzing connectivity patterns in fMRI data of the human brain. PLoS One 5:. https://doi.org/10.1371/journal.pone.0010232
Love MI, Huber W, Anders S (2014) Moderated estimation of fold change and dispersion for RNA-seq data with DESeq2
Lowe TM, Eddy SR (1997) tRNAscan-SE: a program for improved detection of transfer RNA genes in genomic sequence. Nucleic Acids Res 25:955–64. https://doi.org/10.1093/nar/25.5.0955
Lu Q, Ren S, Lu M, et al (2013) Computational prediction of associations between long non-coding RNAs and proteins. BMC Genomics 14:651. https://doi.org/10.1186/1471-2164-14-651
Luke B, Panza A, Redon S, et al (2008) The Rat1p 5′ to 3′ Exonuclease Degrades Telomeric Repeat-Containing RNA and Promotes Telomere Elongation in Saccharomyces cerevisiae. Mol Cell 32:465–477. https://doi.org/10.1016/j.molcel.2008.10.019
Luo W, Brouwer C (2013) Pathview: an R/Bioconductor package for pathway-based data integration and visualization. Bioinformatics 29:1830–1. https://doi.org/10.1093/bioinformatics/btt285
Malikina KD, Shishov VA, Chuvelev DI, et al (2010) [Regulatory role of monoamine neurotransmitters in Saccharomyces cerevisiae cells]. Prikl Biokhim Mikrobiol 46:672–7
Mamnun YM, Schüller C, Kuchler K (2004) Expression regulation of the yeast PDR5 ATP-binding cassette (ABC) transporter suggests a role in cellular detoxification during the exponential growth phase. FEBS Lett 559:111–117. https://doi.org/10.1016/S0014-5793(04)00046-8
Mansure JJ., Panek AD, Crowe LM, Crowe JH (1994) Trehalose inhibits ethanol effects on intact yeast cells and liposomes. Biochim Biophys Acta - Biomembr 1191:309–316. https://doi.org/10.1016/0005-2736(94)90181-3
Martens JA, Wu P-YJ, Winston F (2005) Regulation of an intergenic transcript controls adjacent gene transcription in Saccharomyces cerevisiae. Genes Dev 19:2695–2704. https://doi.org/10.1101/gad.1367605
Martínez-Reyes I, Chandel NS (2020) Mitochondrial TCA cycle metabolites control physiology and disease. Nat Commun 11:102. https://doi.org/10.1038/s41467-019-13668-3
Mason O, Verwoerd M (2007) Graph theory and networks in Biology. IET Syst Biol 1:89–119. https://doi.org/10.1049/iet-syb:20060038
McClintock B (1984) The significance of responses of the genome to challenge. Science (80-) 226:792–801. https://doi.org/10.1126/science.15739260
Miinalainen IJ, Chen Z-J, Torkko JM, et al (2003) Characterization of 2-enoyl thioester reductase from mammals. An ortholog of YBR026p/MRF1'p of the yeast mitochondrial fatty acid synthesis type II. J Biol Chem 278:20154–61. https://doi.org/10.1074/jbc.M302851200
Miller SM, Magasanik B (1990) Role of NAD-linked glutamate dehydrogenase in nitrogen metabolism in Saccharomyces cerevisiae. J Bacteriol 172:4927–4935. https://doi.org/10.1128/jb.172.9.4927-4935.1990
Mistry J, Finn RD, Eddy SR, et al (2013) Challenges in homology search: HMMER3 and convergent evolution of coiled-coil regions. Nucleic Acids Res 41:. https://doi.org/10.1093/nar/gkt263
Mittal P, Ghule K, Trakroo D, et al (2020) Meiosis-Specific Functions of Kinesin Motors in Cohesin Removal and Maintenance of Chromosome Integrity in Budding Yeast. Mol Cell Biol 40:. https://doi.org/10.1128/MCB.00386-19
Nadal-Ribelles M, Solé C, Xu Z, et al (2014) Control of Cdc28 CDK1 by a Stress-Induced lncRNA. Mol Cell 53:549–561. https://doi.org/10.1016/J.MOLCEL.2014.01.006
Nakahara K, Ohkuni A, Kitamura T, et al (2012) The Sjögren-Larsson Syndrome Gene Encodes a Hexadecenal Dehydrogenase of the Sphingosine 1-Phosphate Degradation Pathway. Mol Cell 46:461–471. https://doi.org/10.1016/j.molcel.2012.04.033
Neil H, Malabat C, D'Aubenton-Carafa Y, et al (2009) Widespread bidirectional promoters are the major source of cryptic transcripts in yeast. Nature 457:1038–1042. https://doi.org/10.1038/nature07747
Newman MEJ (2002) Random graphs as models of networks. In: Handbook of Graphs and Networks. pp 35–68
Ni W, Zhang Y, Zhan Z, et al (2017) A novel lncRNA uc.134 represses hepatocellular carcinoma progression by inhibiting CUL4A-mediated ubiquitination of LATS1. J Hematol Oncol 10:91. https://doi.org/10.1186/s13045-017-0449-4
Niederer RO, Hass EP, Zappulla DC (2017) Long Noncoding RNAs in the Yeast S. cerevisiae. In: Rao M (ed) Long Non Coding RNA Biology. Springer, Singapore, pp 119–132
Nissan TA (2002) 60S pre-ribosome formation viewed from assembly in the nucleolus until export to the cytoplasm. EMBO J 21:5539–5547. https://doi.org/10.1093/emboj/cdf547
Novačić A, Vučenović I, Primig M, Stuparević I (2020) Non-coding RNAs as cell wall regulators in Saccharomyces cerevisiae. Crit Rev Microbiol 46:15–25. https://doi.org/10.1080/1040841X.2020.1715340
Owsianik G, Balzi L, Ghislain M (2002) Control of 26S proteasome expression by transcription factors regulating multidrug resistance in Saccharomyces cerevisiae. Mol Microbiol 43:1295–1308. https://doi.org/10.1046/j.1365-2958.2002.02823.x
Panchaud N, Péli-Gulli M-P, De Virgilio C (2013) Amino acid deprivation inhibits TORC1 through a GTPase-activating protein complex for the Rag family GTPase Gtr1. Sci Signal 6:ra42. https://doi.org/10.1126/scisignal.2004112
Panni S, Prakash A, Bateman A, Orchard S (2017) The yeast noncoding RNA interaction network. RNA 23:1479–1492.

https://doi.org/10.1261/rna.060996.117
Parker R (2012) RNA degradation in Saccharomyces cerevisae. Genetics 191:671–702. https://doi.org/10.1534/genetics.111.137265
Parker S, Fraczek MG, Wu J, et al (2017) A resource for functional profiling of noncoding RNA in the yeast Saccharomyces cerevisiae. RNA 23:1166–1171. https://doi.org/10.1261/rna.061564.117
Parker S, Fraczek MG, Wu J, et al (2018) Large-scale profiling of noncoding RNA function in yeast. PLOS Genet 14:e1007253. https://doi.org/10.1371/journal.pgen.1007253
Paulusma CC, Oude Elferink RPJ (2005) The type 4 subfamily of P-type ATPases, putative aminophospholipid translocases with a role in human disease. Biochim Biophys Acta - Mol Basis Dis 1741:11–24. https://doi.org/10.1016/j.bbadis.2005.04.006
Peng Y, Leung HCM, Yiu SM, Chin FYL (2012) IDBA-UD: a de novo assembler for single-cell and metagenomic sequencing data with highly uneven depth. Bioinformatics 28:1420–1428. https://doi.org/10.1093/bioinformatics/bts174
Peng Y, Leung HCM, Yiu SM, Chin FYL (2010) IDBA – A Practical Iterative de Bruijn Graph De Novo Assembler. In: Lecture Notes in Computer Science (including subseries Lecture Notes in Artificial Intelligence and Lecture Notes in Bioinformatics). pp 426–440
Peters LZ, Hazan R, Breker M, et al (2013) Formation and dissociation of proteasome storage granules are regulated by cytosolic pH. J Cell Biol 201:663–671. https://doi.org/10.1083/jcb.201211146
Pomorski T, Lombardi R, Riezman H, et al (2003) Drs2p-related P-type ATPases Dnf1p and Dnf2p are required for phospholipid translocation across the yeast plasma membrane and serve a role in endocytosis. Mol Biol Cell 14:1240–54. https://doi.org/10.1091/mbc.e02-08-0501
Pruyne D, Bretscher A (2000) Polarization of cell growth in yeast. J Cell Sci 113 (Pt 4:571–85
Pruyne D, Evangelista M, Yang C, et al (2002) Role of formins in actin assembly: nucleation and barbed-end association. Science 297:612–5. https://doi.org/10.1126/science.1072309
Pruyne D, Gao L, Bi E, Bretscher A (2004) Stable and dynamic axes of polarity use distinct formin isoforms in budding yeast. Mol Biol Cell 15:4971–89. https://doi.org/10.1091/mbc.e04-04-0296
Qiu Z, Jiang R (2017) Improving Saccharomyces cerevisiae ethanol production and tolerance via RNA polymerase II subunit Rpb7. Biotechnol Biofuels 10:125. https://doi.org/10.1186/s13068-017-0806-0
Quinlan AR, Hall IM (2010) BEDTools: A flexible suite of utilities for comparing genomic features. Bioinformatics 26:841–842. https://doi.org/10.1093/bioinformatics/btq033
Ramachandran V, Shah KH, Herman PK (2011) The cAMP-Dependent Protein Kinase Signaling Pathway Is a Key Regulator of P Body Foci Formation. Mol Cell 43:973–981. https://doi.org/10.1016/j.molcel.2011.06.032
Raudvere U, Kolberg L, Kuzmin I, et al (2019) g:Profiler: a web server for functional enrichment analysis and conversions of gene lists (2019 update). Nucleic Acids Res 47:W191–W198. https://doi.org/10.1093/nar/gkz369
Reimand J, Arak T, Adler P, et al (2016) g:Profiler—a web server for functional interpretation of gene lists (2016 update). Nucleic Acids Res 44:W83–W89. https://doi.org/10.1093/nar/gkw199
Reinders A, Burckert N, Boller T, et al (1998) Saccharomyces cerevisiae cAMP-dependent protein kinase controls entry into stationary phase through the Rim15p protein kinase. Genes Dev 12:2943–2955. https://doi.org/10.1101/gad.12.18.2943
Reinders J, Wagner K, Zahedi RP, et al (2007) Profiling phosphoproteins of yeast mitochondria reveals a role of phosphorylation in assembly of the ATP synthase. Mol Cell Proteomics 6:1896–906. https://doi.org/10.1074/mcp.M700098-MCP200
Repetto B, Tzagoloff A (1989) Structure and regulation of KGD1, the structural gene for yeast alpha-ketoglutarate dehydrogenase. Mol Cell Biol 9:2695–705. https://doi.org/10.1128/mcb.9.6.2695-2705.1989
Rice P, Longden I, Bleasby A (2000) EMBOSS: The European Molecular Biology Open Software Suite. Trends Genet 16:276–277. https://doi.org/10.1016/S0168-9525(00)02024-2
Rieger KJ, Kaniak A, Coppée JY, et al (1997) Large-scale phenotypic analysis--the pilot project on yeast chromosome III. Yeast 13:1547–62. https://doi.org/10.1002/(SICI)1097-0061(199712)13:16<1547::AID-YEA230>3.0.CO;2-Y
Roof DM, Meluh PB, Rose MD (1992) Kinesin-related proteins required for assembly of the mitotic spindle. J Cell Biol 118:95–108. https://doi.org/10.1083/jcb.118.1.95
Saunier R, Esposito M, Dassa EP, Delahodde A (2013) Integrity of the Saccharomyces cerevisiae Rpn11 Protein Is Critical for Formation of Proteasome Storage Granules (PSG) and Survival in Stationary Phase. PLoS One 8:e70357. https://doi.org/10.1371/journal.pone.0070357
Schauer N, Steinhauser D, Strelkov S, et al (2005) GC-MS libraries for the rapid identification of metabolites in complex biological samples. FEBS Lett 579:1332–1337. https://doi.org/10.1016/j.febslet.2005.01.029
Schimmel P (2017) The emerging complexity of the tRNA world: mammalian tRNAs beyond protein synthesis. Nat Rev Mol Cell Biol 19:45–58. https://doi.org/10.1038/nrm.2017.77
Schmalix W, Bandlow W (1993) The ethanol-inducible YAT1 gene from yeast encodes a presumptive mitochondrial outer carnitine acetyltransferase. J Biol Chem 268:27428–39
Schulz D, Schwalb B, Kiesel A, et al (2013) Transcriptome Surveillance by Selective Termination of Noncoding RNA Synthesis. Cell 155:1075–1087. https://doi.org/10.1016/j.cell.2013.10.024
Schulz MH, Zerbino DR, Vingron M, Birney E (2012) Oases: robust de novo RNA-seq assembly across the dynamic range of expression levels. Bioinformatics 28:1086–1092. https://doi.org/10.1093/bioinformatics/bts094
Seattle Proteome Center (SPC) Trans-Proteomic (TPP) pipeline
SGD database (2021) GDH3 expression and reaction. https://pathway.yeastgenome.org/YEAST/new-image?type=PATHWAY&object=GLUNH3-PWY&detail-level=2&EXP-ONLY=NIL. Accessed 7 Jun 2021
Shah KH, Zhang B, Ramachandran V, Herman PK (2013) Processing Body and Stress Granule Assembly Occur by Independent and Differentially Regulated Pathways in Saccharomyces cerevisiae. Genetics 193:109–123. https://doi.org/10.1534/genetics.112.146993
Shani N, Valle D (1996) A Saccharomyces cerevisiae homolog of the human adrenoleukodystrophy transporter is a heterodimer of two half ATP-binding cassette transporters. Proc Natl Acad Sci U S A 93:11901–6. https://doi.org/10.1073/pnas.93.21.11901
Shannon P, Markiel A, Ozier O, et al (2003) Cytoscape: a software environment for integrated models of biomolecular interaction networks. Genome Res 13:2498–504. https://doi.org/10.1101/gr.1239303
Shiflett SL, Vaughn MB, Huynh D, et al (2004) Bph1p, the Saccharomyces cerevisiae Homologue of CHS1/Beige, Functions in Cell Wall Formation and Protein Sorting. Traffic 5:700–710. https://doi.org/10.1111/j.1600-0854.2004.00213.x
Shiratori A, Shibata T, Arisawa M, et al (1999) Systematic identification, classification, and characterization of the open reading frames which encode novel helicase-related proteins inSaccharomyces cerevisiae by gene disruption and Northern analysis. Yeast 15:219–253. https://doi.org/10.1002/(SICI)1097-0061(199902)15:3<219::AID-YEA349>3.0.CO;2-3
Smit A, Hubley R RepeatMasker
Song G, Dickins BJA, Demeter J, et al (2015) AGAPE (Automated Genome Analysis PipelinE) for Pan-Genome Analysis of Saccharomyces cerevisiae. PLoS One 10:e0120671. https://doi.org/10.1371/journal.pone.0120671
Sopko R, Huang D, Preston N, et al (2006) Mapping pathways and phenotypes by systematic gene overexpression. Mol Cell 21:319–30. https://doi.org/10.1016/j.molcel.2005.12.011

Standart N, Weil D (2018) P-Bodies: Cytosolic Droplets for Coordinated mRNA Storage. Trends Genet 34:612–626. https://doi.org/10.1016/j.tig.2018.05.005
Stevenson LF, Kennedy BK, Harlow E (2001) A large-scale overexpression screen in Saccharomyces cerevisiae identifies previously uncharacterized cell cycle genes. Proc Natl Acad Sci U S A 98:3946–51. https://doi.org/10.1073/pnas.051013498
Straight AF, Sedat JW, Murray AW (1998) Time-lapse microscopy reveals unique roles for kinesins during anaphase in budding yeast. J Cell Biol 143:687–94. https://doi.org/10.1083/jcb.143.3.687
Supek F, Bošnjak M, Škunca N, Šmuc T (2011) REVIGO Summarizes and Visualizes Long Lists of Gene Ontology Terms. PLoS One 6:e21800. https://doi.org/10.1371/journal.pone.0021800
Swiegers JH, Dippenaar N, Pretorius IS, Bauer FF (2001) Carnitine-dependent metabolic activities inSaccharomyces cerevisiae: three carnitine acetyltransferases are essential in a carnitine-dependent strain. Yeast 18:585–595. https://doi.org/10.1002/yea.712
Takagi H, Takaoka M, Kawaguchi A, Kubo Y (2005) Effect of <scp>l</scp> -Proline on Sake Brewing and Ethanol Stress in *Saccharomyces cerevisiae*. Appl Environ Microbiol 71:8656–8662. https://doi.org/10.1128/AEM.71.12.8656-8662.2005
Takahashi DY, Sato JR, Ferreira CE, Fujita A (2012) Discriminating Different Classes of Biological Networks by Analyzing the Graphs Spectra Distribution. PLoS One 7:. https://doi.org/10.1371/journal.pone.0049949
Tarailo-Graovac M, Chen N (2009) Using RepeatMasker to identify repetitive elements in genomic sequences. Curr Protoc Bioinformatics Chapter 4:Unit 4.10. https://doi.org/10.1002/0471250953.bi0410s25
Tcheperegine SE, Gao X-D, Bi E (2005) Regulation of cell polarity by interactions of Msb3 and Msb4 with Cdc42 and polarisome components. Mol Cell Biol 25:8567–80. https://doi.org/10.1128/MCB.25.19.8567-8580.2005
Teixeira MC, Raposo LR, Mira NP, et al (2009) Genome-wide identification of Saccharomyces cerevisiae genes required for maximal tolerance to ethanol. Appl Environ Microbiol 75:5761–5772. https://doi.org/10.1128/AEM.00845-09
Teixeira V, Medeiros T, Vilaca R, et al (2014) Reduced TORC1 signaling abolishes mitochondrial dysfunctions and shortened chronological lifespan of Isc1p-deficient cells. Microb Cell 1:21–36. https://doi.org/10.15698/mic2014.01.121
The MIT License Pairfq-Research Computing Center Wiki
Till P, Mach RL, Mach-Aigner AR (2018) A current view on long noncoding RNAs in yeast and filamentous fungi. Appl Microbiol Biotechnol 102:7319–7331. https://doi.org/10.1007/s00253-018-9187-y
Timmers HTM, Tora L (2018) Transcript Buffering: A Balancing Act between mRNA Synthesis and mRNA Degradation. Mol Cell 72:10–17. https://doi.org/10.1016/j.molcel.2018.08.023
Tkach JM, Yimit A, Lee AY, et al (2012) Dissecting DNA damage response pathways by analysing protein localization and abundance changes during DNA replication stress. Nat Cell Biol 14:966–976. https://doi.org/10.1038/ncb2549
Valachovic M, Garaiova M, Holic R, Hapala I (2016) Squalene is lipotoxic to yeast cells defective in lipid droplet biogenesis. Biochem Biophys Res Commun 469:1123–1128. https://doi.org/10.1016/j.bbrc.2015.12.050
van Dijk EL, Chen CL, D'Aubenton-Carafa Y, et al (2011) XUTs are a class of Xrn1-sensitive antisense regulatory non-coding RNA in yeast. Nature 475:114–117. https://doi.org/10.1038/nature10118
van Leeuwen W, Rabouille C (2019) Cellular stress leads to the formation of membraneless stress assemblies in eukaryotic cells. Traffic tra.12669. https://doi.org/10.1111/tra.12669
van Roermund CW, Hettema EH, van den Berg M, et al (1999) Molecular characterization of carnitine-dependent transport of acetyl-CoA from peroxisomes to mitochondria in Saccharomyces cerevisiae and identification of a plasma membrane carnitine transporter, Agp2p. EMBO J 18:5843–52. https://doi.org/10.1093/emboj/18.21.5843
van Werven FJ, Neuert G, Hendrick N, et al (2012) Transcription of Two Long Noncoding RNAs Mediates Mating-Type Control of Gametogenesis in Budding Yeast. Cell 150:1170–1181. https://doi.org/10.1016/j.cell.2012.06.049
Wang C, Schmich F, Srivatsa S, et al (2018) Context-dependent deposition and regulation of mRNAs in P-bodies. Elife 7:. https://doi.org/10.7554/eLife.29815
Weinberger M, Mesquita A, Caroll T, et al (2010) Growth signaling promotes chronological aging in budding yeast by inducing superoxide anions that inhibit quiescence. Aging (Albany NY) 2:709–26. https://doi.org/10.18632/aging.100215
Wiwie C, Kuznetsova I, Mostafa A, et al (2019) Time-Resolved Systems Medicine Reveals Viral Infection-Modulating Host Targets. Syst Med 2:1–9. https://doi.org/10.1089/sysm.2018.0013
Woudstra EC, Gilbert C, Fellows J, et al (2002) A Rad26-Def1 complex coordinates repair and RNA pol II proteolysis in response to DNA damage. Nature 415:929–33. https://doi.org/10.1038/415929a
Wu TD, Watanabe CK (2005) GMAP: a genomic mapping and alignment program for mRNA and EST sequences. Bioinformatics 21:1859–1875. https://doi.org/10.1093/bioinformatics/bti310
Wu W-I, McDonough VM, Nickels JT, et al (1995) Regulation of Lipid Biosynthesis in Saccharomyces cerevisiae by Fumonisin B 1. J Biol Chem 270:13171–13178. https://doi.org/10.1074/jbc.270.22.13171
Xiao T, Khan A, Shen Y, et al (2022) Glucose feeds the tricarboxylic acid cycle via excreted ethanol in fermenting yeast. Nat Chem Biol. https://doi.org/10.1038/s41589-022-01091-7
Xu K, Bezakova I, Bunimovich L, Yi S V. (2011) Path lengths in protein-protein interaction networks and biological complexity. Proteomics 11:1857–1867. https://doi.org/10.1002/pmic.201000684
Xu Z, Wei W, Gagneur J, et al (2009) Bidirectional promoters generate pervasive transcription in yeast. Nature 457:1033–1037. https://doi.org/10.1038/nature07728
Yamashita A, Shichino Y, Yamamoto M (2016) The long non-coding RNA world in yeasts. Biochim Biophys Acta - Gene Regul Mech 1859:147–154. https://doi.org/10.1016/j.bbagrm.2015.08.003
Yang T-H, Wang C-C, Wang Y-C, Wu W-S (2014) YTRP: a repository for yeast transcriptional regulatory pathways. Database (Oxford) 2014:bau014. https://doi.org/10.1093/database/bau014
Yifrach E, Holbrook-Smith D, Bürgi J, et al (2022) Systematic multi-level analysis of an organelle proteome reveals new peroxisomal functions. Mol Syst Biol 18:. https://doi.org/10.15252/msb.202211186
Yoshikawa K, Tanaka T, Ida Y, et al (2011) Comprehensive phenotypic analysis of single-gene deletion and overexpression strains of Saccharomyces cerevisiae. Yeast 28:349–61. https://doi.org/10.1002/yea.1843
Yu Y, Yarrington RM, Chuong EB, et al (2016) Disruption of promoter memory by synthesis of a long noncoding RNA. Proc Natl Acad Sci 113:9575–9580. https://doi.org/10.1073/pnas.1601793113
Zampar GG, Kümmel A, Ewald J, et al (2013) Temporal system-level organization of the switch from glycolytic to gluconeogenic operation in yeast. Mol Syst Biol 9:651. https://doi.org/10.1038/msb.2013.11
Zappulla DC, Cech TR (2004) Yeast telomerase RNA: A flexible scaffold for protein subunits. Proc Natl Acad Sci 101:10024–10029. https://doi.org/10.1073/pnas.0403641101
Zerbino DR, Birney E (2008) Velvet: Algorithms for de novo short read assembly using de Bruijn graphs. Genome Res 18:821–829. https://doi.org/10.1101/gr.074492.107
Zhang B, Herman PK (2020) It is all about the process(ing): P-body granules and the regulation of signal transduction. Curr Genet 66:73–77. https://doi.org/10.1007/s00294-019-01016-3
Zhang T, Lei J, Yang H, et al (2011) An improved method for whole protein extraction from yeast Saccharomyces cerevisiae. Yeast

28:795–798. https://doi.org/10.1002/yea.1905
Ziman M, Chuang JS, Schekman RW (1996) Chs1p and Chs3p, two proteins involved in chitin synthesis, populate a compartment of the Saccharomyces cerevisiae endocytic pathway. Mol Biol Cell 7:1909–1919. https://doi.org/10.1091/mbc.7.12.1909
